# Patient-reported outcome instruments used in immune-checkpoint inhibitor clinical trials in oncology: a systematic review

**DOI:** 10.1186/s41687-020-00210-z

**Published:** 2020-07-16

**Authors:** Sara Colomer-Lahiguera, Denise Bryant-Lukosius, Sarah Rietkoetter, Lorraine Martelli, Karin Ribi, Donna Fitzpatrick-Lewis, Diana Sherifali, Angela Orcurto, Rosalyn Juergens, Manuela Eicher

**Affiliations:** 1 Institute of Higher Education and Research in Healthcare (IUFRS), Faculty of Biology and Medicine, University of Lausanne, Lausanne, Switzerland; 2 Department of Oncology, Lausanne University Hospital (CHUV), Lausanne, Switzerland; 3 School of Nursing, Faculty of Health Sciences, McMaster University, Hamilton, ON Canada; 4 Juravinski Hospital and Cancer Centre at Hamilton Health Sciences, Hamilton, ON Canada; 5 Department of Oncology, Faculty of Health Sciences, McMaster University, Hamilton, ON Canada; 6 Trillium Health Partners, Mississauga, ON Canada; 7 International Breast Cancer Study Group (IBCSG), Coordinating Center, Bern, Switzerland; 8 McMaster Evidence and Review Synthesis Team, Faculty of Health Sciences, McMaster University, Hamilton, ON Canada; 9 Population Health Research Institute, Hamilton Health Sciences, McMaster Evidence and Review Synthesis Team, School of Nursing, Faculty of Health Sciences, McMaster University, Hamilton, ON Canada

**Keywords:** Adverse events, Clinical trial, Immune-checkpoint inhibitors, Oncology, Patient-reported outcomes, Symptoms

## Abstract

**Context:**

Immune-checkpoint inhibitors (ICI) have shown significant benefits for overall survival across various cancer types. Patient-reported outcomes (PROs) are assessed in clinical trials as a measure of efficacy. However, it remains unclear to what extent current PRO instruments capture symptoms specific to ICI toxicities. We conducted a systematic review to identify the use and content validity of PRO instruments in ICI clinical trials in oncology.

**Methods:**

Literature was retrieved from PubMed, Embase, PsycINFO, Medline and CINAHL databases. Articles presenting ICI clinical trials’ PRO results, clinical trial study protocols, and conference abstracts stating the use of PRO measures were assessed. We evaluated the validity of identified instruments by comparing their symptom-related content with the adverse events reported in each ICI clinical trial.

**Results:**

From database inception until January 2020, we identified 191 ICI clinical trials stating the use of PRO measures of which 26 published PRO results. The cancer-specific EORTC QLQ-C30 and the generic EQ-5D questionnaires were the most widely used instruments, often in combination with disease-specific PROs. Instruments used to report PRO symptom-related toxicities covered 45% of the most frequently reported AEs, whereas 23% of AEs were partially covered and 29% were not covered at all. Of non-covered AEs, 59% referred to the dermatologic system. Partially covered AEs related to endocrine and specific types of pain.

**Conclusion:**

Despite the high frequency of symptom-related toxicities related to ICI, these events are only partially covered (or not addressed) by current PRO instruments, even when combined. Further research is needed to develop new strategies to tailor PRO instruments to specific ICI toxicities.

## Introduction

Immune-checkpoint inhibitors (ICIs) have shown significant clinical benefit by improving treatment response and survival rates for different cancer patient populations [1]. ICIs are associated with specific toxicities related to inflammatory or autoimmune responses - known as immune-related adverse events (irAEs) that affect different organs and systems [2]. These irAEs result from broad and unspecific immune over-activation [3]. In most cases, toxicities are reversible with corticosteroid or additional immunosuppressant treatment but some rare and potentially life-threatening irAEs have been reported [3]. Early recognition of irAEs and timely management is essential to reduce morbidity and mortality [4].

In clinical trials (CTs), the Common Terminology Criteria for Adverse Events (CTCAE) is the most widely used method to report toxicities [5]. Of these, 10% focus on symptomatic adverse events (AEs) amenable to patient self-report [6]. Thus, clinicians are often required to evaluate subjective patient experiences – resulting in potential underreporting of AEs [7, 8].

Patient-reported outcomes (PROs) are defined as “any report of the status of a patient’s health condition that comes directly from the patient, without interpretation of the patient’s response by a clinician or anyone else” [9]. Several studies demonstrate that compared to clinician report, PROs are more concordant with patient overall health status and result in earlier detection of symptom occurrence and severity [10, 11]. Thus, patient-reported symptom assessment that incorporates items relevant to ICIs may facilitate early AE detection and may provide complementary data to support decision-making.

The overall aim of the study was to identify and categorize the types and combination of PRO instruments used in CTs involving cancer patients receiving ICI therapy (ICI-CTs), and to assess their frequency of use. To further examine the content validity of the PRO instruments, we identified and compared the most frequent AEs reported in ICI-CTs with published PRO results.

## Methods

### Literature search

This systematic review was conducted according to the Preferred Reporting Items for Systematic Reviews and Meta-Analysis (PRISMA) statement [12] and the protocol was prospectively registered with PROSPERO (registration number: CRD42018090912). A literature search was performed in PubMed, Embase, PsycINFO, Medline and CINAHL databases using search terms adapted to the respective database. We used broad parameters employing controlled vocabulary (MeSH/Emtree preferred and related concepts) as well as specific keywords related to three constructs: (1) ICI terms (e.g. FDA-approved ICI), (2) PRO terms and (3) oncology and immunotherapy terms (Table S1 in the Supplementary Information). To ensure a comprehensive search strategy, we used the CT identification number from selected conference abstracts and CT study protocols to find additional CTs with published PRO results. The search was conducted from database inception to June 26, 2017 and updated on January 22, 2020. Articles written in English, French, Spanish, and German were included.

### Selection criteria

Four pairs of reviewers (SCL, SR, LM, DBL, ME, NK, AC, AA) screened titles and abstracts following a predefined list of inclusion/exclusion criteria (Table S2). A Cohen’s kappa test was performed in order to ensure consistency between reviewers (κ = 0.81). Full texts of publications and conference abstracts were obtained and reviewed independently by two pairs of reviewers (SCL, SR, KR, LM) to identify articles for final analysis. Disagreements were resolved by discussion to reach consensus.

### Types of publications included

**Fig. 1:**
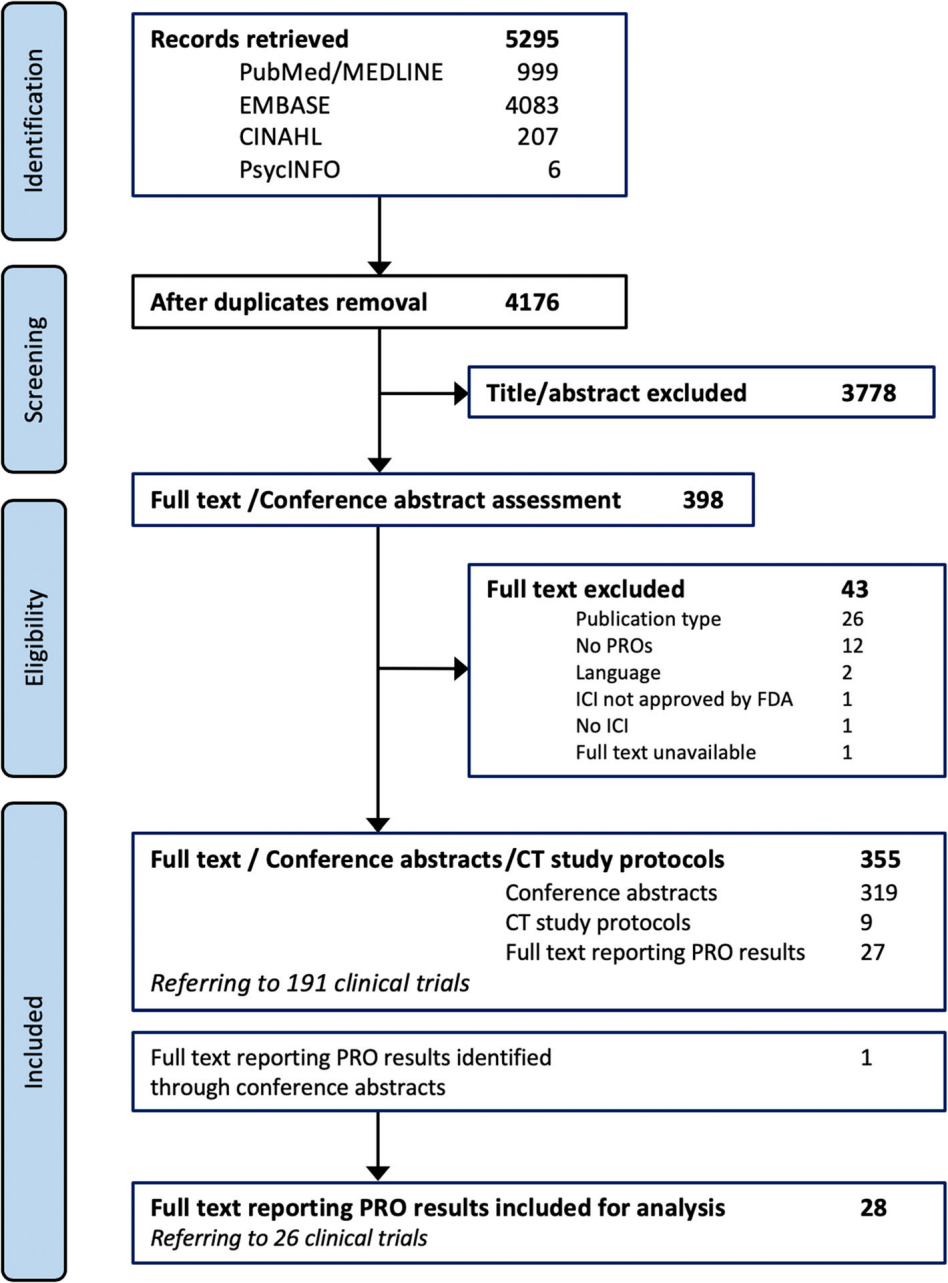
PRISMA chart mapping the number of records identified, selected, and included for final analysis. Twenty-eight full-text articles reporting PRO results referring to 26 ICI-CTs, 9 study protocols, and 319 conference abstracts stating the use of PRO measures in ICI-CTs, were retained for data extraction

Three types of publications were included (Fig. 1). Full-text articles with PRO results served to evaluate the reporting of PRO in ICI-CTs. CT study protocols and conference abstracts were used to assess the use and combination of PRO instruments in ICI-CTs and to identify additional full-text articles reporting on PRO results in ICI-CTs.

### Data extraction

Reviewers (SR, LM, KR, DFL) completed a structured, online form using Distiller SR, to extract data from each full-text article including study and ICI-CT characteristics, analysis, results, study conclusions, and information about the PRO instruments used in the study (Table S3). Second reviewers (SCL and DS) verified the extracted data. For CT study protocols and conference abstracts, the following data were extracted: first author, publication year, study characteristics (name of the clinical trial/study, CT identifier, start year, cancer type, and number of participants), PRO endpoints, and name of the PRO instrument(s). Collected information was completed and verified across sources: clinicaltrials.gov/EudraCT, published CT protocol (when available), and primary CT publication (Table S4).

### Categorization, combination and frequency of use of PRO instruments

Identified PRO instruments for all ICI-CTs were grouped into categories based on the focus of the measure (e.g. generic, cancer-specific, disease/tumor-specific, symptom-specific, item bank/single items, or other) and their frequency of use and combination were subsequently analyzed. The *Cochrane checklist for describing and assessing PROs in CTs* [13] was applied to the full-text articles presenting PRO results to assess measurement properties of the PRO instruments and PROs reporting (Table S5).

### Comparison of PRO symptom content and most frequent reported AEs

We examined the content validity of PRO instruments used in the full-text articles reporting PRO results by comparing the extent to which the symptom-related content of identified PRO instruments (Table S7) aligned with the ten most frequent AEs reported in their respective CT study publication (Tables S8). A panel of experts including oncologists, advanced practice oncology nurses, and oncology nursing researchers reviewed and, through consensus, agreed on the differentiation of complex AEs into multi-symptoms that can be reported by patients (Table S9). AEs were considered ‘covered’ when the item matched all corresponding symptoms contained in the PRO instrument. Conversely, items were considered ‘not covered’ when PRO instruments did not include the corresponding symptom. Multi-symptom AEs were considered ‘partially covered’ when at least two of the symptoms pre-defined (Table S9) were contained in the PRO instrument(s). Coverage of the most frequently reported AEs (for each ICI-CT arm) were calculated and reported as a percentage.

## Results

The search yielded 5295 records. After removing duplicates and screening titles and abstracts, 398 publications remained. Subsequent full-text assessment identified 27 articles reporting ICI-CTs PRO results, 319 conference abstracts, and 9 published CT study protocols. An additional article reporting PRO results was identified through conference abstracts and was included in the final analysis (total articles *n* = 28) (Fig. 1). Detailed review of articles, CT study protocols, and conference abstracts identified 191 ICI-CTs. We found information on PRO instruments for 156 of these ICI-CTs (Figure S1 and Table S4).

### Categorization, combination and frequency of use of PRO instruments in ICI-CTs

**Fig. 2:**
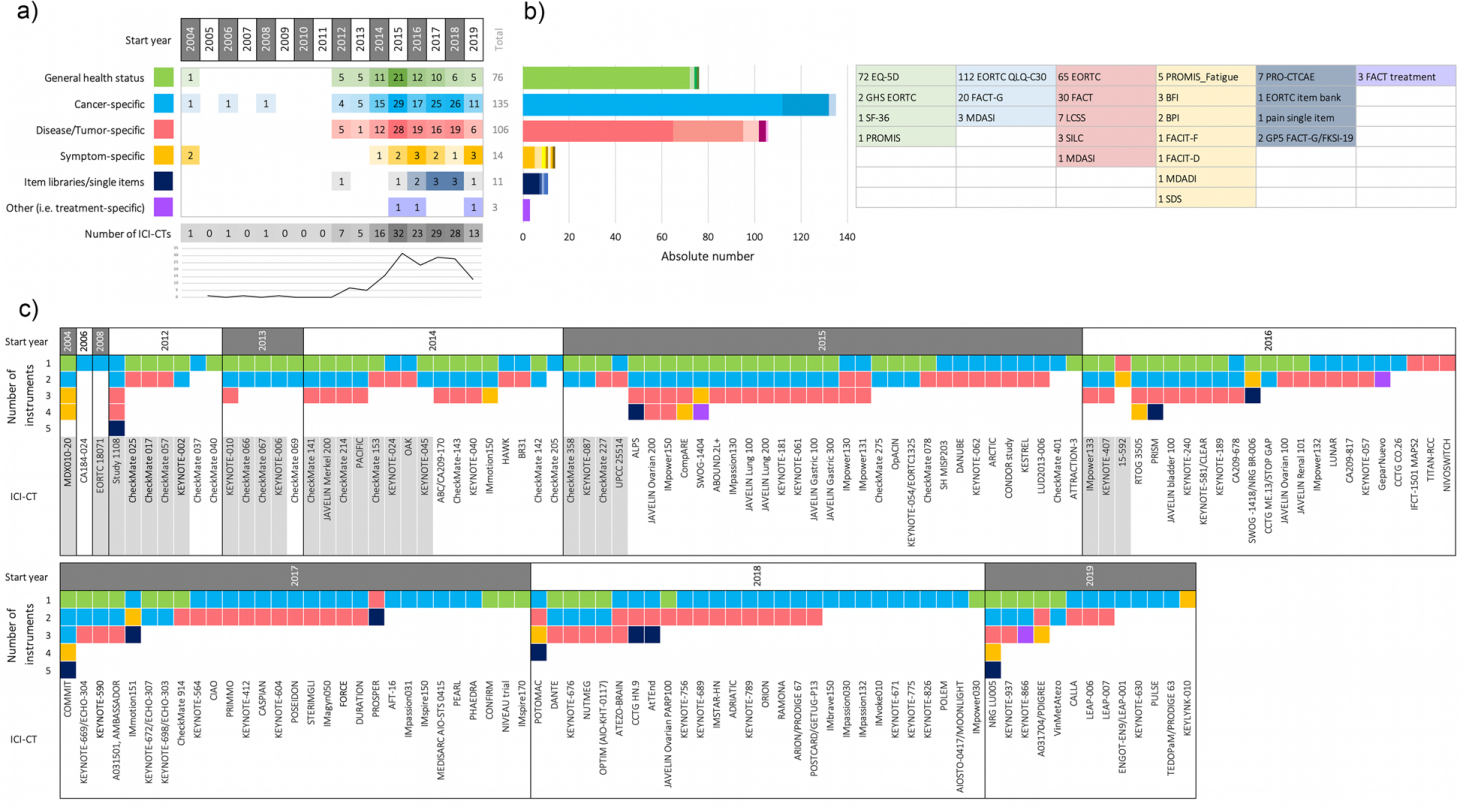
Frequency of use and combination of PRO instruments in ICI-CTs (*n* = 156). **a** Number of PRO instruments divided into categories and start year of the ICI-CTs. Green: general health status; Blue: cancer-specific; Red: disease/tumor-specific; Yellow: symptom-specific; Dark blue: items libraries/single items; Purple: other (e.g. treatment-specific). **b** Absolute number of instruments across all ICI-CTs divided per category. The table shows the different instruments per category. **c** Number and combination of PRO instruments per ICI-CT per year of start of the trial. For FACT questionnaires that include core domains of the FACT-G, both cancer-specific and disease specific have been depicted (see Table S4 for more detail). For ICI-CTs colored in light grey PRO results have been published (*n* = 26)

The number, categorization, and combination of PRO instruments for each of the 156 ICI-CTs is shown in Fig. 2 and supplementary Table S4. Data review and analysis of the 156 ICI-CTs showed an increase over the years of the number of ICI-CTs stating the use or measurement of PROs in publications or conference abstracts (Fig. 2a). The peak was reached in 2015, with 32 ICI-CTs, followed by a plateau in the consecutive years. In the same period of time an increase of ICI-CTs can be observed (Figure S1).

Of the 156 ICI-CTs, 35 used one instrument, 68 used two instruments, 41 used three instruments, and 12 used four or five instruments (Fig. 2c and Table S4). Among the 68 ICI-CTs using two instruments, 16 paired generic with cancer-specific, 10 paired generic with disease/tumor-specific, 39 paired cancer-specific and disease/tumor-specific, and the rest (*n* = 3) combined a disease/tumor or cancer-specific with a symptom-specific questionnaire (*n* = 1), a treatment-specific (n = 1), or selected items (n = 1). In studies using three instruments, combining generic, cancer-specific, and disease-specific questionnaires was the most commonly used approach (*n* = 30/41). The other 11 ICI-CTs combined generic or cancer-specific instruments with disease-specific and symptoms or single item from item-libraries. Of the 12 ICI-CTs that used four or five instruments, most of them combined generic, cancer-specific and disease/tumor-specific with either symptom questionnaires or single items selected from item libraries such as the Patient Reported Outcomes Measurement Information System (PROMIS), the PRO version of the CTCAE (PRO-CTCAE), or the European Organisation for Research and treatment of Cancer (EORTC) (Fig. 2c).

The cancer-specific EORTC Quality of Life Questionnaire Core 30 (EORTC QLQ-C30) was the most widely used PRO instrument (112/156, 72%), followed by the generic health EuroQol 5 Dimension Scale (EQ-5D) (72/156, 46%) (Fig. 2b).

Focusing on the evolution of the use of PROs over time, we observe two main periods. Between 2004 and 2013, few studies used single instruments with the majority (8/15) of studies using two instruments. Between 2014 and 2019 (*n* = 141), we observe a higher variability in the number of instruments used (Fig. 2a).

### Articles reporting on PRO results

From the 28 full-text articles reporting PRO results, one discussed associations between biomarkers and PRO results, 18 reported PRO results alone, and seven presented safety and efficacy data from the main ICI-CT together with PRO results. For two of the latter, additional full-text manuscripts on PRO results alone were published. Thus, the 28 publications referred to 26 individual ICI-CTs on lung carcinoma (*n* = 11) [14–24], melanoma (*n* = 6) [25–30], renal cell carcinoma (*n* = 3) [31–33], urothelial carcinoma (*n* = 2) [34, 35], Hodgkin’s lymphoma (n = 1) [36, 37], head and neck carcinoma (n = 1) [38, 39], Merkel cell carcinoma (n = 1) [40], and cervical and vaginal carcinoma (n = 1) [41] (Table S5). With regard to measured PRO concepts, all publications reported on health-related quality of life (HRQoL) or global health status (GHS) as assessed by the cancer-specific EORTC QLQ-C30 (17/26) and/or the generic EQ-5D (15/26). Eleven publications additionally included the measure of disease-related symptoms, and another four added a function assessment. In general, the selection of the PRO instrument(s) was justified based on previous use and validation in similar patient populations - either general cancer patients or disease-specific populations (Table S5). Only one publication [40] re-validated the instrument in the study population and the psychometric results have been published elsewhere [42].

Overall, most of the publications reported differences between groups in terms of “clinically meaningful deterioration”, “clinically meaningful improvement”, “clinically relevant”, “stable”, “minimal change”, “statistically significant”, or “significant difference” (Table S5). The primary reasons for attrition and missing PRO data were disease progression and AEs (15/26) (Table S6).

### Content validity of PRO instruments

**Fig. 3:**
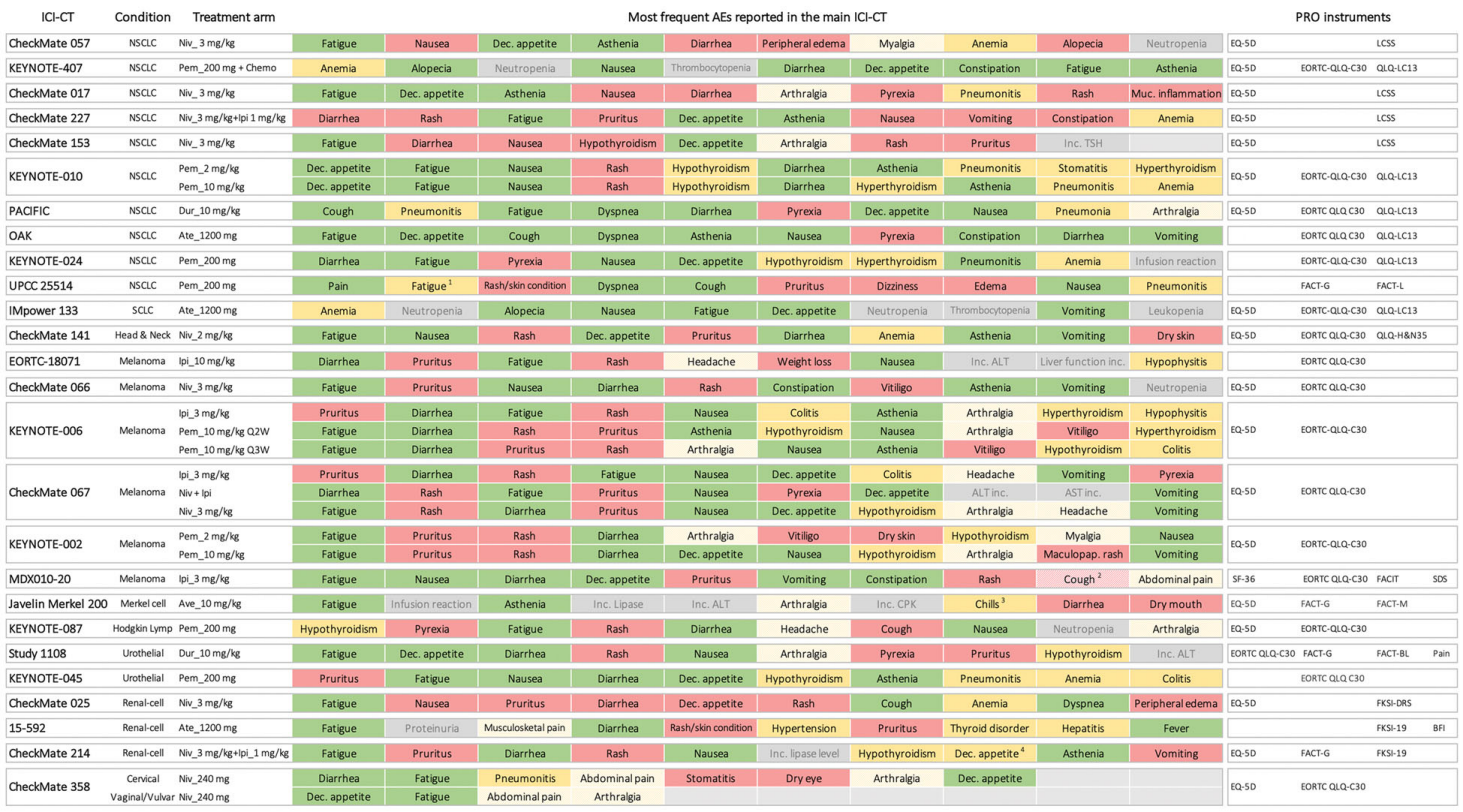
Comparison of PRO instruments’ symptom-related content and AEs reported in ICI-CTs with published PRO results (*n* = 26). The most frequently reported AEs (any grade) are shown for the 26 ICI-CTs identified. AE frequency (most common to least common) is depicted from left to right respectively. Symptom-related content from each PRO instrument was compared to the AEs in the corresponding trial arm. The PRO instruments used in each ICI-CT is shown. For a detailed PRO instrument content, see Table S8. Green: AEs covered; Yellow: AEs partially covered; Light yellow: AEs partially covered related to specific types of pain; Red: AEs not covered. Laboratory tests results (grey) were not included in calculations. “Asthenia” was considered covered by “fatigue” following the National Cancer Institute toxicity grading scale version 3 that included asthenia, lethargy, and malaise under the umbrella of “fatigue”. (1) “Fatigue” was considered partially covered because the term included in the FACT-G was “lack of energy”; (2) While the study protocol announced the use of SF-36, EORTC QLQ-C30, FACIT-Fatigue, and SDS questionnaires, only results from the EORTC QLQ-C30 were reported in the full-text publication. “Cough” would be covered by the SDS instrument; (3) “Chills” was partially covered as related to “fever”, included in the FACT-G; (4) “Dec. appetite” was considered as partially covered by the term “good appetite” in the FKSI-19. Ipi, Ipilimumab; Niv, Nivolumab; Pem, Pembrolizumab; Ate, Atezolizumab; Dur, Durvalumab; Inc., increased; Dec, decreased

The use of the Cochrane checklist (Table S5) revealed a lack of coverage of common ICI-related AEs. We extracted the symptom-related content of each PRO instrument used in each of the 26 ICI-CTs identified (Table S7), followed by the extraction of the AEs reported in the main CT publication (Table S8). We then charted the ten most frequent AEs (any grade) reported in the respective ICI-CTs denoting which AEs were covered by PRO instruments employed in the corresponding study (Fig. 3). In total, almost half of the AEs (142/299, 47%) were covered by PRO instrument(s) employed in the ICI-CTs, even when used in combination. Nearly a third of AEs reported in ICI-CTs were not covered by the PRO instrument(s) (86/299, 29%). Among ‘not covered’ AEs, 51/86 (59%) were dermatologic in nature (e.g. rash, pruritus, vitiligo, dry skin). Further, multi-symptom AEs were partially covered in 71/299 (24%) of which 34% (24/71) related to a specific type of pain (e.g. headache, arthralgia, myalgia) and 28% (20/71) were endocrine-related (e.g. hypothyroidism, hyperthyroidism, hypophysitis).

## Discussion

This systematic review examines the use of PRO instruments in clinical trials of FDA-approved ICI (up to January 2020). Our analysis reveals the cancer-specific EORTC QLQ-C30 and the generic EQ-5D questionnaires are the most widely used PRO instruments (72% and 46%, respectively) - either alone or in combination with disease-specific instruments. Although we observed an increase in the stated use of PRO measurements in ICI-CTs, reaching a peak in 2015, this should be seen in the global context of ICI development and the increase in ICI-CTs over the years. HRQoL or QoL are the most frequent measured concepts and only one third of the articles included additionally disease-specific symptom measures. Furthermore, our results indicate that the used PRO instruments did not include items specifically relating to ICI treatment-related symptoms. These results raise important concerns about the comprehensiveness of current instruments and the measurement of relevant PROs in the context of ICI-CTs. Similar conclusions have been drawn in other recently reported studies of PROs in ICI-CTs [43, 44]. However, the rigorous approach used in our review offers new, more detailed, and compelling insights about the use and content validity of PRO instruments in ICI-CTs. First, we provide a more comprehensive assessment of the aggregate content of PRO instruments in relation to frequent AEs by identifying and categorizing all the PRO instruments used in each ICI-CT (Fig. 3). In contrast, Hall et al. [44] only examined PRO instruments related to symptoms contained in the EORTC questionnaire. Secondly, our study examined the full spectrum of AEs reported in each ICI-CT (Fig. 3) as opposed to King-Kallimanis et al., who limited their examination to eight pre-selected symptoms for anti-PD-1/PD-L1 agents [43].

The use of PROs in CTs has grown in the last decade and PROs are recommended as important endpoints for trials [9, 45]. However, the availability of validated questionnaires and use of the most suitable instruments remains a challenge urging caution when interpreting HRQoL data in ICI-CTs [46–48]. A recent review of studies that led to the approval of ICIs identified several methodological problems with PRO measurement. Specifically, general HRQoL measures might lack sensitivity to provide a comprehensive assessment of the impact that ICI therapies have on patients [49]. For instance, some studies report no differences in HRQoL of ICI-treated patients despite relatively frequent grade 3–4 toxicity. Broadly, ICI-CTs results suggest a stable or overall increased HRQoL yet specific ICI treatment-related symptoms and AEs are not adequately assessed by existing PRO tools. In our study, we identified that dermatologic, endocrine, and specific types of pain are among the most reported ICI-related problems regardless of cancer type/diagnosis. Despite the frequency of such events, these AEs are only partially covered if at all by currently applied PRO instruments.

Studies included in this analysis often employed a combination of different PRO instruments (e.g. generic and cancer-specific, cancer-specific with disease-specific and/or symptom-specific questionnaires). The advantage of generic instruments is their broad applicability for evaluating general aspects of HRQoL independent of the specific diagnosis. However, generic instruments tend to lack responsiveness for detecting deterioration in specific health contexts. Disease- or symptom-specific instruments may be more acceptable and responsive to detecting changes but have limited generalizability for identifying unanticipated events across different populations [13]. In the present review, studies employed PRO instruments based on the intended measure (e.g. to detect changes in HRQoL, GHS, specific disease-related symptoms). Closely examining ICI-related toxicities revealed a unique profile - independent of cancer diagnosis [50–52]. This observation challenges the practice of choosing PRO instruments based on the population rather than treatment type. In other words, both cancer-specific tools and generic HRQoL questionnaires designed for traditional chemotherapies may not capture symptoms and toxicities specific to ICI therapies. Following this line of thought, Kluetz and colleagues have suggested the individual measurement of symptomatic adverse events, physical function, and disease-related symptoms. These three concepts are highly relevant to HRQoL and may better reflect the therapy’s effect on the patient and the patient’s disease [53]. An alternative approach to the traditional static instruments is the use of item libraries or computerized adaptive testing with content and symptom-items adapted for ICI treatments such as the National Cancer Institute PRO-CTCAE, PROMIS or the EORTC item library [6, 54–56]. Recently, efforts have been undertaken to develop such ICI specific modules [57].

Our study highlights the general underreporting of PRO results. Of 3231 ICI-CTs registered in the period between 2004 and 2019, only 191 (6%) had a published statement (study protocol, conference abstract) about the measurement of PROs, of which only 26 had a full-text article on PRO results. This finding aligns with the results from a recent systematic evaluation of PROs in cancer trials by Kyte et al. [58]. Importantly, PROs inform and facilitate treatment decision-making in clinical practice. As such, systematic PRO collection, analysis and reporting are highly relevant and should be considered a priority. This delay in PRO reporting is at odds with recommendations from both the European and American oncology societies (ESMO and ASCO) to incorporate such measures in clinical trials as read-outs for treatment benefit evaluation [59, 60].

## Conclusion

Our systematic review on the use of PROs in ICI-CTs identifies variability in the use of instruments and gaps in existing instruments for addressing symptomatic toxicities that are ICI treatment-related. Further work is needed to develop novel strategies that permit the more comprehensive and systematic collection of PRO data specific to ICI treatments to inform and support drug labelling and clinical decision-making.

## Supplementary information

**Additional file 1: Table S1.** List with the search terms. **Table S2.** List with the inclusion / exclusion criteria. **Table S3.** Data extraction. **Figure S1.** ICI-CT registered in clinicaltrials.gov (2004–2019). **Table S4.** List of identified clinical trials stating the use of PROs. **Table S5.***Cochrane checklist for describing and assessing PROs in CTs.***Table S6.** PRO instruments completion rates. **Table S7.** Symptom-related content of PRO instruments. **Table S8.** AEs reported in selected ICI-CT. **Table S9.** AEs differentiated into multi-symptom.

**Additional file 2: Figure S1.** Frequency of use and combination of PRO instruments in ICI-CTs (*n* = 156). **a** Number of PRO instruments divided into categories and start year of the ICI-CTs. Green: general health status; Blue: cancer-specific; Red: disease/tumor-specific; Yellow: symptom-specific; Dark blue: items libraries/single items; Purple: other (e.g. treatment-specific). **b** Absolute number of instruments across all ICI-CTs divided per category. The table shows the different instruments per category. **c** Number and combination of PRO instruments per ICI-CT per year of start of the trial. For FACT questionnaires that include core domains of the FACT-G, both cancer-specific and disease specific have been depicted (see Table S4 for more detail). For ICI-CTs colored in light grey PRO results have been published (*n* = 26)

**Additional file 3: Figure S2.** Comparison of PRO instruments’ symptom-related content and AEs reported in ICI-CTs with published PRO results (*n* = 26). The most frequently reported AEs (any grade) are shown for the 26 ICI-CTs identified. AE frequency (most common to least common) is depicted from left to right respectively. Symptom-related content from each PRO instrument was compared to the AEs in the corresponding trial arm. The PRO instruments used in each ICI-CT is shown. For a detailed PRO instrument content, see Table S8. Green: AEs covered; Yellow: AEs partially covered; Light yellow: AEs partially covered related to specific types of pain; Red: AEs not covered. Laboratory tests results (grey) were not included in calculations. “Asthenia” was considered covered by “fatigue” following the National Cancer Institute toxicity grading scale version 3 that included asthenia, lethargy, and malaise under the umbrella of “fatigue”. (1) “Fatigue” was considered partially covered because the term included in the FACT-G was “lack of energy”; (2) While the study protocol announced the use of SF-36, EORTC QLQ-C30, FACIT-Fatigue, and SDS questionnaires, only results from the EORTC QLQ-C30 were reported in the full-text publication. “Cough” would be covered by the SDS instrument; (3) “Chills” was partially covered as related to “fever”, included in the FACT-G; (4) “Dec. appetite” was considered as partially covered by the term “good appetite” in the FKSI-19. Ipi, Ipilimumab; Niv, Nivolumab; Pem, Pembrolizumab; Ate, Atezolizumab; Dur, Durvalumab; Inc., increased; Dec, decreased

## Data Availability

Protocols from the clinical trial identified and/or links to the clinical trial registration as well as individual clinical trial information are provided in Supplementary Information.

## Abbreviations

ICI: Immune-checkpoint inhibitor
CT: Clinical trial
ICI-CT: Immune-checkpoint inhibitor clinical trial
AE: Adverse event
irAE: Immune-related adverse event
CTCAE: Common Terminology Criteria for Adverse Events
PRO: Patient-reported outcome
EMA: European Medicines Agency
FDA: Food and Drug Administration
EORTC QLQ-C30: European Organisation for Research and treatment of Cancer Quality of Life Questionnaire Core 30
EQ-5D: Euroqol 5 dimension
FACT: Functional Assessment of Cancer Therapy
FACIT: Functional Assessment of Chronic Illness Therapy
FKSI: FACT-Kidney Symptom Index
SDS: Symptom Distress Scale
FKSI: FACT-Kidney Symptom Index
HRQoL: Health-related quality of life
GHS: Global health status
PRO-CTCAE: Patient-Reported Outcomes Common Terminology Criteria for Adverse Events
PROMIS: Patient-Reported Outcomes Measurement Information System
ESMO: European Society for Medical Oncology
ASCO: American Society of Clinical Oncology
