# Supplementary material for: Patient-reported outcome instruments used in immune-checkpoint inhibitor clinical trials in oncology: a systematic review
Source: J Patient Rep Outcomes. 2020 Jul 16;4:58. doi: 10.1186/s41687-020-00210-z (PMC7364679; doi:10.1186/s41687-020-00210-z)
Supplement: Supplementary file 1 — Additional file 1: Table S1. List with the search terms. Table S2. List with the inclusion / exclusion criteria. Table S3. Data extraction. Figure S1. ICI-CT registered in clinicaltrials.gov (2004–2019). Table S4. List of identified clinical trials stating the use of PROs. Table S5.Cochrane checklist for describing and assessing PROs in CTs.Table S6. PRO instruments completion rates. Table S7. Symptom-related content of PRO instruments. Table S8. AEs reported in selected ICI-CT. Table S9. AEs differentiated into multi-symptom. [file 41687_2020_210_MOESM1_ESM.pdf]

Patient reported outcome instruments used in immune checkpoint inhibitor clinical trials in oncology: a systematic review

Sara Colomer-Lahiguera, Denise Bryant-Lukosius, Sarah Rietkoetter, Lorraine Martelli, Karin Ribi, Donna Fitzpatrick-Lewis, Diana Sherifali, Angela Orcurto, Rosalyn Juergens, Manuela Eicher.

Supplementary information

Table S1: List with the search terms

Table S2: List with the inclusion / exclusion criteria

Table S3: Data extraction

Figure S1: ICI-CT registered in clinicaltrials.gov (2004-2019)

Table S4: List of identified clinical trials stating the use of PROs

Table S5: *Cochrane checklist for describing and assessing PROs in CTs*

Table S6: PRO instruments completion rates

Table S7: Symptom-related content of PRO instruments

Table S8: AEs reported in selected ICI-CT

Table S9: AEs differentiated into multi-symptom

References

Table S1: List with the search terms

	PROs terms	Oncology & Immunotherapy terms	Checkpoint Inhibitor terms
MEDLINE & PubMed	exp Patient Reported Outcome Measures/<br>patient reported outcome*.mp.<br>self report.mp. or exp Self Report/<br>"PROM".mp.<br>"PROMs".mp.<br>“PROs”.mp.<br>(patient report* adj2 (outcome or measure or assessment or questionnaire or instrument or index or indice* or indicator*)).ti,ab.<br>(patient derived adj2 (outcome or measure or assessment or questionnaire or instrument or index or indice* or indicator*)).ti,ab.<br>(patient rated adj2 (outcome or measure or assessment or questionnaire or instrument or index or indice* or indicator*)).ti,ab.<br>(patient based adj2 (outcome or measure or assessment or questionnaire or instrument or index or indice* or indicator*)).ti,ab.<br>quality of life.mp. or exp "Quality of Life"/<br>life quality.mp.<br>QOL.mp.<br>HRQOL.mp.<br>HR-QOL.mp.<br>HRQL.mp.<br>QL.mp.<br>health utilit*.mp.	exp Immunotherapy/<br>immunotherap*.mp.<br>exp Neoplasms/<br>neoplasm*.mp.<br>cancer*.mp.<br>oncolog*.mp.<br>(tumor or tumour).mp.<br>malignan*.mp.	exp CTLA-4 Antigen/<br>CTLA*.mp.<br>exp T-Lymphocytes, Cytotoxic/<br>(CD152 or CD 152).mp.<br>ipilimumab.mp.<br>yervoy.mp.<br>("MDX 010" or MDX010).mp.<br>exp Programmed Cell Death 1 Receptor/<br>programmed cell death.mp.<br>(PD1 or PD 1).mp.<br>(CD279 or CD 279).mp.<br>nivolumab.mp.<br>(MDX1106 or MDX 1106).mp.<br>(ono4538 or ono 4538).mp.<br>(BMS936558 or BMS 936558).mp.<br>opdivo.mp.<br>pembrolizumab.mp.<br>lambrolizumab.mp.<br>keytruda.mp.<br>(MK3475 or MK 3475).mp.<br>programmed death.mp.<br>(PDL1 or PDL 1).mp.<br>exp Antigens, CD274/<br>(CD274 or CD 274).mp.<br>Atezolizumab.mp.<br>(MPDL3280A or MPDL 3280A).mp.<br>tecentriq.mp.<br>(RG7446 or RG 7446).mp.<br>Avelumab.mp.<br>(MSB0010718C or MSB 0010718C).mp.<br>Durvalumab.mp.<br>bavencio.mp.<br>imfinzi.mp.<br>checkpoint blockade.mp.<br>checkpoint inhibitor.mp.<br>immune checkpoint.mp.<br>immune blockade.mp.

	PROs terms	Oncology & Immunotherapy terms	Checkpoint Inhibitor terms
EMBASE	patient reported outcome*.mp.<br>PROMs.mp.<br>PROM.mp.<br>health related quality of life.mp.<br>life quality.mp.<br>QOL.mp.<br>HRQOL.mp.<br>QL.mp.<br>HRQL.mp.<br>HR-QOL.mp.<br>health utilit*.mp.<br>exp patient-reported outcome/<br>quality of life.mp. or exp "quality of life"/<br>self report.mp. or exp self report/<br>(patient report* adj2 (outcome or measure or assessment or questionnaire or instrument or index or indice* or indicator*)).ti,ab.<br>(patient derived adj2 (outcome or measure or assessment or questionnaire or instrument or index or indice* or indicator*)).ti,ab.<br>(patient rated adj2 (outcome or measure or assessment or questionnaire or instrument or index or indice* or indicator*)).ti,ab.<br>(patient based adj2 (outcome or measure or assessment or questionnaire or instrument or index or indice* or indicator*)).ti,ab.	exp immunotherapy/ or exp cancer immunotherapy/<br>immunotherap*.mp.<br>exp neoplasm/ or neoplasm*.mp.<br>cancer*.mp.<br>oncolog*.mp.<br>(tumor or tumour).mp.<br>malignan*.mp.	exp cytotoxic T lymphocyte antigen 4/ or ctla*.mp.<br>exp cytotoxic T lymphocyte/<br>CD152 CD 152.mp.<br>ipilimumab.mp.<br>yervoy.mp.<br>("MDX 010" or MDX010).mp.<br>programmed cell death.mp. or exp apoptosis/<br>(PD1 or PD 1).mp.<br>(CD279 or CD 279).mp.<br>nivolumab.mp.<br>(MDX1106 or MDX 1106).mp.<br>ono4538 ono 4538.mp.<br>(BMS936558 or BMS 936558).mp.<br>opdivo.mp.<br>pembrolizumab.mp.<br>lambrolizumab.mp.<br>keytruda.mp.<br>(MK3475 or MK 3475).mp.<br>exp programmed death 1 ligand 1/ or programmed death.mp. or exp programmed death 1 receptor/<br>(PDL1 or PDL 1).mp.<br>(CD274 or CD 274).mp.<br>Atezolizumab.mp.<br>(MPDL3280A or MPDL 3280A).mp.<br>tecentriq.mp.<br>(RG7446 or RG 7446).mp.<br>avelumab.mp.<br>(MSB0010718C or MSB 0010718C).mp.<br>durvalumab.mp.<br>bavencio.mp.<br>imfinzi.mp.<br>checkpoint blockade.mp.<br>checkpoint inhibitor.mp.<br>immune checkpoint.mp.<br>immune blockade.mp.

	PROs terms	Oncology & Immunotherapy terms	Checkpoint Inhibitor terms
CINAHL	(MH “Patient-Reported Outcomes+”)<br>“patient reported outcome*”<br>(MH “Self Report+”) OR “self report”<br>“PROM”<br>“PROMs”<br>TI patient report* N2 (outcome or measure or assessment or questionnaire or instrument or index or indice* or indicator*<br>AB patient report* N2 (outcome or measure or assessment or questionnaire or instrument or index or indice* or indicator*)<br>TI patient derived N2 (outcome or measure or assessment or questionnaire or instrument or index or indice* or indicator*)<br>AB patient derived N2 (outcome or measure or assessment or questionnaire or instrument or index or indice* or indicator*)<br>TI patient rated N2 (outcome or measure or assessment or questionnaire or instrument or index or indice* or indicator*)<br>AB patient rated N2 (outcome or measure or assessment or questionnaire or instrument or index or indice* or indicator*)<br>TI patient based N2 (outcome or measure or assessment or questionnaire or instrument or index or indice* or indicator*)<br>AB patient based N2 (outcome or measure or assessment or questionnaire or instrument or index or indice* or indicator*)<br>(MH “Quality of Life”) OR “quality of life”<br>“life quality”<br>“QOL”<br>“HRQOL”<br>“HR-QOL”<br>“HRQL”<br>QL<br>“health utilit*”	(MH “Immunotherapy+”)<br>“immunotherap*”<br>(MH “Neoplasms+”)<br>“neoplasm*”<br>“cancer*”<br>“oncolog*”<br>“tumor”<br>“tumour”<br>“malignan*”	“CTLA*”<br>(MH “T Lymphocytes”)<br>“CD152”<br>“ipilimumab”<br>“yervoy”<br>“MDX 010”<br>“programmed cell death”<br>“PD1”<br>“CD279”<br>“nivolumab”<br>“MD1106”<br>“ono4538”<br>BMS936558”<br>“opdivo”<br>“pembrolizumab”<br>“lambrolizumab”<br>“keytruda”<br>“MK3475”<br>“programmed death”<br>“PDL1”<br>“CD274”<br>“atezolizumab”<br>“MPDL3280A”<br>“tecentriq”<br>“RG7446”<br>“avelumab”<br>“MSB0010718C”<br>“durvalumab”<br>“bavencio”<br>“imfinzi”<br>“checkpoint blockade”<br>“checkpoint inhibitor”<br>“immune checkpoint”<br>“immune blockade”

	PROs terms	Oncology & Immunotherapy terms	Checkpoint Inhibitor terms
PsycINFO	Exp Self-Report/<br>Patient reported outcome*.mp.<br>Self report.mp.<br>PROM.mp.<br>PROMs.mp.<br>(patient report* adj2 (outcome or measure or assessment or questionnaire or instrument or index or indice* or indicator*)).ti,ab.<br>(patient derived adj2 (outcome or measure or assessment or questionnaire or instrument or index or indice* or indicator*)).ti,ab.<br>(patient rated adj2 (outcome or measure or assessment or questionnaire or instrument or index or indice* or indicator*)).ti,ab.<br>(patient based adj2 (outcome or measure or assessment or questionnaire or instrument or index or indice* or indicator*)).ti,ab.<br>Quality of life.mp. or exp “Quality of Life”/<br>Life quality.mp.<br>QOL.mp.<br>HRQOL.mp.<br>HR-QOL.mp.<br>HRQL.mp.<br>QL.mp.<br>Health utilit*.mp.	Exp Immunotherapy/<br>Immunotherap*.mp.<br>Exp Neoplasms/<br>Neoplasm*.mp.<br>Cancer*.mp.<br>Oncolog*.mp.<br>(tumor or tumour).mp.<br>Malignan*.mp.	CTLA*.mp.<br>(CD152 or CD 152).mp.<br>Ipilimumab.mp.<br>Yervoy.mp.<br>(“MDX 010 or MDX010).mp.<br>Programmed cell death.mp.<br>(PD1 or PD 1).mp.<br>(CD279 or CD 279).mp.<br>Nivolumab.mp.<br>(MDX1106 or MDX 1106).mp.<br>(ono4538 or ono 4538).mp.<br>(BMS936558 or BMS 936558).mp.<br>Opdivo.mp.<br>Pembrolizumab.mp.<br>Lambrolizumab.mp.<br>Keytruda.mp.<br>(MK3475 or MK 3475).mp.<br>Programmed death.mp.<br>(PDL1 or PDL 1).mp.<br>(CD274 or CD 274).mp.<br>Atezolizumab.mp.<br>(MPDL3280A or MPDL 3280A).mp.<br>Tecentriq.mp.<br>(RG7446 or RG 7446).mp.<br>Avelumab.mp.<br>(MSB0010718C or MSB 0010718C).mp.<br>Durvalumab.mp.<br>Bavencio.mp.<br>Imfinzi.mp.<br>Checkpoint blockade.mp.<br>Checkpoint inhibitor.mp.<br>Immune checkpoint.mp.<br>Immune blockade.mp.

Table S2: List with the inclusion / exclusion criteria

**Inclusion criteria:**
• Clinical trials, intervention studies, study protocols, and conference abstracts from intervention studies<br>• Studies involving patients receiving treatment for cancer<br>• Investigating FDA approved immune checkpoint inhibitors (Ipilimumab, Nivolumab, Pembrolizumab/Lambrolizumab, Atezolizumab, Avelumab, Durvalumab)<br>• Use of patient reported outcome (PRO) instruments
**Exclusion criteria:**
• Articles that do not report on empirical/interventional studies (including discussion papers, commentaries, editorials, opinion papers, clinical practice guidelines, systematic reviews of the literature, QALY/cost-effectiveness studies)<br>• Articles reporting follow-up results from clinical trials or subgroup analyses of main clinical trials<br>• Studies evaluating ICI therapies for patients with conditions or diseases other than cancer (e.g. autoimmune: rheumatoid arthritis, Parkinson disease...)<br>• Investigating other cancer immunotherapies that are not immune checkpoint inhibitors:<br>• vaccines<br>• adoptive therapy: CAR-T cells/Tumor infiltrating lymphocytes TILs/cytotoxic T cells CTL<br>• other monoclonal antibodies<br>• targeted therapy<br>• oncolytic viruses<br>• CTLA4-Ig, Abatacept or Belatacept<br>• Articles referring to clinician reported performance status (ECOG, Karnofsky, etc...) or other instruments that are not completed or reported by patients.
**Special considerations**
• Conference abstracts and study protocols will be reviewed separately in order to identify further published or unpublished studies matching the inclusion criteria.<br>• Articles containing “Quality of life” (QoL) in the abstract as background information and not as an outcome of the study will be excluded.<br>• Articles involving PROs will be included as long as one of the populations analysed in the study is treated with ICI.<br>• Articles including inflammatory or autoimmune diseases as immune-related adverse events (irAEs) derived from ICI treatment that analyse QoL will be included.

Table S3: Data extraction form

REVIEWER NAME:

1. Title
2. First author
3. Journal
4. Year of publication

STUDY CHARACTERISTICS

5. Name of the study
6. Clinical trial ID (if applicable)
7. Duration of the study (if available)
    a. Started (year)
    b. Ended (year)
8. Funding source /sponsor
    a. Company
    b. Grant
9. QALY study
    o Yes
    o No
10. What is the stated purpose of the study?
11. Study design
    ☐ Clinical trial
        12. o Phase I
            o Phase II
            o Phase III
        13. o Controlled / comparative
            o Open-label
    ☐ Observational
    ☐ Randomized
    ☐ Controlled
    ☐ Case report
    ☐ Case series
    ☐ Interventional study
    ☐ Systematic review
    ☐ Other (specify)

14. Disease / condition

15. Intervention group
    ☐ Nivolumab
    ☐ Pembrolizumab
    ☐ Ipilimumab
    ☐ Atezolizumab
    ☐ Avelumab
    ☐ Durvalumab
    ☐ Combination (specify)

16. Control group
    ☐ Placebo
    ☐ Other (specify)
    ☐ Not applicable

17. Type of treatment
    ☐ First line
    ☐ Second line
    ☐ Third line
    ☐ Adjuvant/ combination (specify)

- ☐ Other (specify)
- ☐ Not reported

18. Inclusion criteria

19. Exclusion criteria

20. Study sample size:
    - ☐ Total (n)
    - ☐ Control group (n)
    - ☐ Intervention group (n)

21. PRO tool completion at baseline
    - ☐ Total (n)
    - ☐ Control group (n)
    - ☐ Intervention group (n)

22. Study attrition
    - ☐ Total (n) end of study
    - ☐ Control group (n) end of study
    - ☐ Intervention group (n) end of study
    - ☐ Reason(s) for attrition reported in the study

23. PRO tool attrition
    - ☐ Total (n) end of study
    - ☐ Control group (n) end of study
    - ☐ Intervention group (n) end of study
    - ☐ Reason(s) for attrition reported in the study

24. Missing study data – reason(s) reported in the study

25. Missing PRO tool data – reason(s) reported in the study

26. Notes/comments

ANALYSIS, RESULTS, CONCLUSIONS

1. Describe the methods used for the data analysis

2. Statistical significance
    - o..Yes (specify)
    - o..No (specify reason)

3. Clinical relevance
    - o..Yes (specify)
    - o..No

4. Results (as stated by the author)

5. Main findings/conclusions (as stated by the author)

Methodological issues related to the measurement of the PRO

6. Authors comments about the methods (i.e. overpowered, compliance, etc…)

7. Authors comments about the tool(s) (i.e. validity, specificity, limitations, etc…)

8. Reviewers comments

Other references related to this study (same population sample)

9. Original Clinical Trial study

10. Other PRO studies

11. QALY studies

PRO tool

Name of the PRO

Was the PRO **used as**

- ○ Primary outcome
- ○ Secondary outcome
- ○ Exploratory endpoint
- ○ Not reported

Type of **concept reported** by the PRO

- ☐ Symptom
  - ☐ Specific (specify)
  - ☐ Generic (includes different symptoms)
- ☐ Quality of life
  - ☐ Global health status
  - ☐ Physical functioning
  - ☐ Role
  - ☐ Emotional
  - ☐ Cognitive
  - ☐ Social
  - ☐ Financial
  - ☐ Other (specify)
- ☐ Experience
- ☐ Toxicities / adverse events
- ☐ Satisfaction
- ☐ Health needs
- ☐ Other (specify)
- ☐ Not defined

Is the **PRO**

- ☐ Generic/health status
- ☐ Cancer specific
- ☐ Disease site/condition specific
- ☐ Symptom specific
- ☐ Outcome specific

Method of administration

- ☐ Paper and pencil
- ☐ Electronic device (tablet, smartphone)
- ☐ Telephone
- ☐ Interview
- ☐ Web-based / on-line
- ☐ Not reported
- ☐ Other (specify)

Schedule of administration / timepoints: (i.e. Baseline, week 9 and every 6 weeks up to 2 years) or not reported

PRO **data**

- ○ Reported
- ○ Not reported

Method for the data analysis

- ☐ Regression
- ☐ Post-hoc
- ☐ Meta-analysis
- ☐ Economic
- ☐ Other (specify)

Figure S1: ICI-CT registered in clinicaltrials.gov (2004-2019)

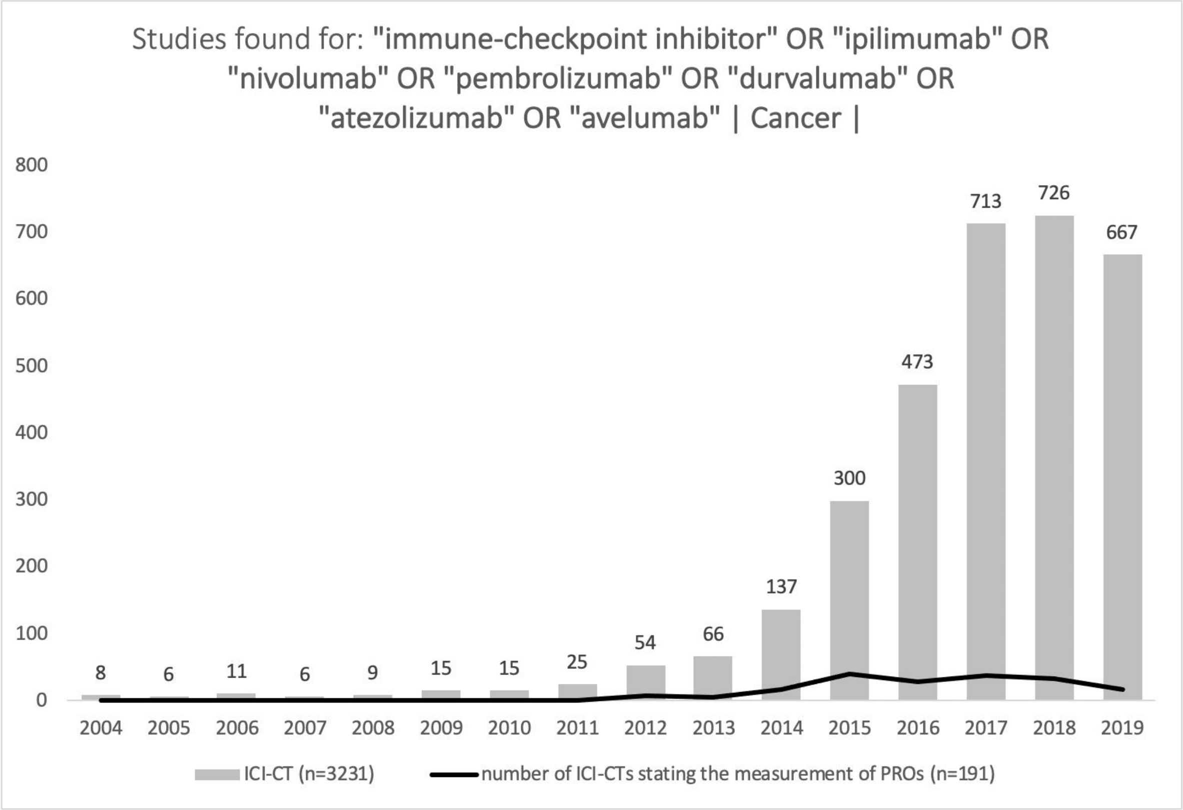

Source: clinicaltrials.gov
Start date from 01/01/2004 to 31/12/2019

Table S4: List of identified clinical trials stating the use of PROs

The following instruments include FACT-G domains and have been categorized as cancer-specific (indicated in italic) and disease-specific: FACT-L; FACT-M; FACT-BL; FACT-Hep; FACT-Cx; FACT-H&N; FACT-BRM; FACT-BL-Cys; FACT-Taxane; FACIT-D.

	Trial	Trial ID	Condition / disease	Drug	Phase	Participants	Start	Endpoint	instruments					Source for PRO instrument identification	Comments
1	15-592	NCT02724878	Advanced Non-Clear Cell Kidney Cancer	Atezolizumab	2	60	2016	S			FKSI-19	BFI		conf abstract + clin trials + publication	
2	516-005	NCT03906071	Metastatic Non-Squamous Non-Small Cell Lung Cancer	Nivolumab	3	532	2019	S	NR					NR	
3	A031501, AMBASSADOR	NCT03244384	Bladder Cancer and Locally Advanced Urothelial Cancer	Pembrolizumab	3	739	2017	E	EQ-5D	EORTC QLQ-C30	QLQ-BLM30			conf abstract + sponsor website	https://www.uchicagomedicine.org/find-a-clinical-trial/clinical-trial/b/cirb171483
4	A031704, PDIGREE	NCT03793166	Advanced Kidney Cancer	Ipilimumab / Nivolumab	3	1046	2019	NR	EQ-5D		FKSI-19	PROMIS Fatigue		conf abstract	
5	ABC / CA209-170	NCT02374242	Melanoma / Brain Metastases	Ipilimumab / Nivolumab	2	76	2014	S	EQ-5D	EORTC QLQ-C30	QLQ-BM20			conf abstract	
6	ABOUND.2L+	NCT02250326	Non-Small-Cell Lung	Durvalumab	2	240	2015	E	EQ-5D	EORTC QLQ-C30	LCSS			conf abstract + protocol	https://clinicaltrials.gov/ProvidedDocs/26/NCT02250326/Prot_001.pdf
7	ADRIATIC	NCT03703297	Small Cell Lung Cancer	Durvalumab / Tremelimumab	3	600	2018	S		EORTC QLQ-C30	QLQ-LC13			clin trials	
8	AFT-16	NCT03102242	Non-Small-Cell Lung	Atezolizumab	2	64	2017	S		EORTC QLQ-C30				conf abstract	
9	AIOSTO-0417-MOONLIGHT	NCT03647969	Stomach-GastroEsophageal Cancer	Nivolumab / Ipilimumab	2	119	2018	S		EORTC QLQ-C30				conf abstract	
10	ALPS	NCT02558894	Pancreatic Ductal Adenocarcinoma	Durvalumab / Tremelimumab	2	65	2015	S	EQ-5D	EORTC QLQ-C30	QLQ-PAN26		PRO-CTCAE	protocol	https://clinicaltrials.gov/ProvidedDocs/94/NCT02558894/Prot_000.pdf
11	ARCTIC	NCT02352948	Non-Small-Cell Lung	Durvalumab	3	597	2015	S		EORTC QLQ-C30	QLQ-LC13			protocol	https://clinicaltrials.gov/ProvidedDocs/48/NCT02352948/Prot_001.pdf
12	ARION/PRODIGE67-UCGI33	NCT03777813	Esophagus Cancer	Durvalumab	2	120	2018	S		EORTC QLQ-C30	QLQ-OES18			clin trials	
13	ATEZO-BRAIN	NCT03526900	Non-Small-Cell Lung	Atezolizumab	2	40	2018	E		EORTC QLQ-C30	QLQ-LC13 + QLQ-BN20			clin trials	
14	ATOMIC, A021502	NCT02912559	Colon Adenocarcinoma	Atezolizumab	3	700	2017	S	NR					NR	
15	AtTEnd	NCT03603184	Endometrial Cancer	Atezolizumab	3	550	2018	S		EORTC QLQ-C30	QLQ-EN24	GP5 item FACT-G		clin trials	GP5 item FACT-G " *I am bothered by side effects of treatment*"
16	ATTRACTION-3	NCT02569242	Esophageal Cancer	Nivolumab	3	390	2015	E	EQ-5D					CT study publication	https://doi.org/10.1016/S1470-2045(19)30626-6
17	BR31	NCT02273375	Non-Small-Cell Lung	Durvalumab	3	1360	2014	S		EORTC QLQ-C30	QLQ-LC13			clin trials	

	Trial	Trial ID	Condition / disease	Drug	Phase	Participants	Start	Endpoint	instruments					Source for PRO instrument identification	Comments
18	CA045-001	NCT03635983	Melanoma	Nivolumab	3	764	2018	E	NR					NR	
19	CA184-024	NCT00324155	Melanoma	Ipilimumab	3	681	2006	S		EORTC QLQ-C30				Conf abstract + protocol	https://www.nejm.org/doi/suppl/10.1056/NEJMoa1104621/suppl_file/nejmoa1104621_protocol.pdf
20	CA184-316 (ILP+/-IPI)	NCT02094391	Melanoma	Ipilimumab	2	4	2014	S	NR					NR	
21	CA209-678	NCT03033446	HepatoCellular Carcinoma	Nivolumab	2	40	2016	S	EORTC QLQ-C30	*FACT-G*	FACT-HEP			conf abstract + clin trials	
22	CA209-817	NCT02869789	Lung Cancer	Ipilimumab / Nivolumab	4	1100	2016	S		*FACT-G*	FACT-L			conf abstract + clin trials	
23	CALLA	NCT03830866	Locally Advanced Cervical Cancer	Durvalumab	3	714	2019	S		EORTC QLQ-C30	QLQ-CX24			conf abstract + clin trials	
24	CAPRA	NCT02565992	Melanoma	Pembrolizumab	1	36	2015	S	NR						
25	CASPIAN	NCT03043872	Small Cell Lung Carcinoma Extensive Disease	Durvalumab / Tremelimumab	3	988	2017	S		EORTC QLQ-C30	QLQ-LC13			conf abstract + clin trials	
26	CCTG BR-34	NCT03057106	Lung Cancer Metastatic	Durvalumab / Tremelimumab	2	301	2015	S	NR					NR	
27	CCTG CO.26	NCT02870920	Colorectal Cancer	Durvalumab / Tremelimumab	2	179	2016	S		EORTC QLQ-C30				conf abstract + clin trials	
28	CCTG HN.9	NCT03410615	Oropharyngeal Squamous Cell Carcinoma	Durvalumab / Tremelimumab	2	180	2018	S		*FACT-G*	FACT H&N		PRO-CTCAE	clin trials	
29	CCTG ME.13/STOP GAP	NCT02821013	Unresectable/Metastatic Melanoma	Pembrolizumab / Nivolumab	3	614	2016	S	EQ-5D	EORTC QLQ-C30				clin trials	
30	CCTG PA.7	NCT02879318	Pancreatic Adenocarcinoma	Durvalumab / Tremelimumab	2	180	2016	S	NR					NR	
31	Checkmate 017	NCT01642004	Non-Small Cell Lung Cancer	Nivolumab	3	352	2012	S	EQ-5D		LCSS			conf abstract + clin trials + protocol + publication PRO results	https://www.nejm.org/doi/suppl/10.1056/NEJMoa1504627/suppl_file/nejmoa1504627_protocol.pdf
32	CheckMate 025	NCT01668784	Renal cell carcinoma	Nivolumab	3	1068	2012	S	EQ-5D		FKSI-DRS			conf abstract + clinicaltrials.gov + protocol	https://www.nejm.org/doi/suppl/10.1056/NEJMoa1510665/suppl_file/nejmoa1510665_protocol.pdf
33	CheckMate 037	NCT01721746	Melanoma	Nivolumab	3	631	2012	S		EORTC QLQ-C30				clin trials	https://www.sciencedirect.com/science/article/pii/S1470204515700768?via%3Dihub
34	CheckMate 040	NCT01658878	hepatocellular carcinoma	Nivolumab	1/2	620	2012	E	EQ-5D					CT study publication	https://www.sciencedirect.com/science/article/pii/S0140673617310462?via%3Dihub
35	CheckMate 057	Nct01673867	Non-Small Cell Lung Cancer	Nivolumab	3	792	2012	E	EQ-5D		LCSS			conf abstract + publication PRO results	
36	Checkmate 066	NCT01721772	Melanoma	Nivolumab	3	583	2013	S	EQ-5D	EORTC QLQ-C30				conf abstract + protocol + publication PRO results	https://www.nejm.org/doi/suppl/10.1056/NEJMoa1412082/suppl_file/nejmoa1412082_protocol.pdf
37	CheckMate 067	Nct01844505	Melanoma	Ipilimumab / Nivolumab	3	1296	2013	S	EQ-5D	EORTC QLQ-C30				conf abstract + protocol + publication PRO results	https://www.nejm.org/doi/suppl/10.1056/NEJMoa1504030/suppl_file/nejmoa1504030_protocol.pdf
38	CheckMate 069	NCT01927419	Melanoma	Ipilimumab / Nivolumab	2	179	2013	S	EQ-5D	EORTC QLQ-C30				conf abstract	https://www.nejm.org/doi/suppl/10.1056/NEJMoa1414428/suppl_file/nejmoa1414428_protocol.pdf
39	CheckMate 078	NCT02613507	Non-Small Cell Lung Cancer	Nivolumab	3	639	2015	S	EQ-5D		LCSS			protocol	https://clinicaltrials.gov/ProvidedDocs/07/NCT02613507/Prot_000.pdf

	Trial	Trial ID	Condition / disease	Drug	Phase	Participants	Start	Endpoint	instruments					Source for PRO instrument identification	Comments
40	CheckMate 141	NCT02105636	Squamous Cell Carcinoma of the Head and Neck	Nivolumab	3	506	2014	E	EQ-5D	EORTC QLQ-C30	QLQ-H&N35			conf abstract + publication PRO results	https://www.nejm.org/doi/suppl/10.1056/NEJMoa1602252/suppl_file/nejmoa1602252_protocol.pdf
41	CheckMate 142	NCT02060188	Colorectal Cancer	Ipilimumab / Nivolumab	2	340	2014	E	EQ-5D	EORTC QLQ-C30				conf abstract	https://www.thelancet.com/journals/lanonc/article/PIIS1470-2045(17)30422-9/fulltext
42	CheckMate 153	NCT02066636	Non-Small Cell Lung Cancer	Nivolumab	3	1380	2014	E	EQ-5D		LCSS			publication PRO results	
43	CheckMate 205	NCT02181738	Hodgkin Lymphoma	Nivolumab	2	338	2014	E		EORTC QLQ-C30				conf abstract + protocol	https://clinicaltrials.gov/ProvidedDocs/38/NCT02181738/Prot_000.pdf
44	CheckMate 214	NCT02231749	Advanced Renal Cell Carcinoma	Ipilimumab / Nivolumab	3	1390	2014	E	EQ-5D	FACT-G	FKSI-19			conf abstract + publication PRO results	
45	CheckMate 227	NCT02477826	Non-Small Cell Lung Cancer	Ipilimumab / Nivolumab	3	2220	2015	E	EQ-5D		LCSS			conf abstract + publication PRO results	
46	CheckMate 275	NCT02387996	urothelial cancer	Nivolumab	2	386	2015	E	EQ-5D	EORTC QLQ-C30				pubication	https://www.sciencedirect.com/science/article/pii/S1470204517300657?via%3Dihub
47	CheckMate 358	NCT02488759	Various Advanced Cancer	Ipilimumab / Nivolumab	1/2	1100	2015	E	EQ-5D	EORTC QLQ-C30				publication PRO results	https://www.ncbi.nlm.nih.gov/pubmed/31487218?dopt=Abstract
48	CheckMate 401	NCT02599402	Melanoma	Ipilimumab / Nivolumab	3	615	2015	E		EORTC QLQ-C30				conf abstract	
49	CheckMate 870	NCT03195491	Non-Small Cell Lung Cancer	Nivolumab	3	400	2017	E	NR					NR	
50	CheckMate 914	NCT03138512	Renal Cell Carcinoma	Ipilimumab / Nivolumab	3	800	2017	E	EQ-5D		FKSI-19			abstract	
51	CHECKMATE-143	NCT02017717	Recurrent Glioblastoma	Nivolumab	3	626	2014	E	EQ-5D	EORTC QLQ-C30	QLQ-BN20			conf abstract	
52	CheckMate-459	NCT02576509	hepatocellular carcinoma	Nivolumab	3	726	2015	E	NR					NR	
53	CIAO	NCT03144778	Oropharyngeal Squamous Cell Carcinoma	Durvalumab / Tremelimumab	1	28	2017	S		*MDASI*	MDASI-HN			clin trials	
54	COMMIT	NCT02997228	Colorectal Cancer	Atezolizumab	3	347	2017	E	EQ-5D	EORTC QLQ-C30	FACT-G	PROMIS Fatigue	PRO-CTCAE	clin trials	
55	CompARE	NCT04116047	Oropharyngeal Cancer	Durvalumab	3	695	2015	S	EQ-5D	EORTC QLQ-C30	QLQ-H&N35	MDADI (Dysphagia)		clin trials	
56	CONDOR study	NCT02319044	squamous cell carcinoma of the head and neck	Durvalumab / Tremelimumab	2	267	2015	S		EORTC QLQ-C30	QLQ-H&N35			clin trials	https://clinicaltrials.gov/ct2/show/NCT02319044
57	CONFIRM	NCT03063450	Mesothelioma	Nivolumab	3	336	2017	S	EQ-5D					clin trials + protocol	https://trialsjournal.biomedcentral.com/articles/10.1186/s13063-018-2602-y
58	D6020C00001	NCT02118337	Select Advanced Malignancies, Kidney Cancer, Clear Cell Renal Cell Carcinoma	durvalumab	1/2	97	2014	E	NR					NR	
59	DANTE	EudraCT2017-002435-42	metastatic melanoma	Pembrolizumab / Nivolumab	4	1208	2018	S	EQ-5D	EORTC QLQ-C30	QLQ-MEL38			WHO international CT registry platform	http://apps.who.int/trialsearch/Trial2.aspx?TrialID=EUCTR2017-002435-42-GB
60	DANUBE	NCT02516241	Urothelial Cancer	Durvalumab / Tremelimumab	3	1200	2015	S		*FACT-G*	FACT-BL			clin trials	

	Trial	Trial ID	Condition / disease	Drug	Phase	Participants	Start	Endpoint	instruments					Source for PRO instrument identification	Comments
61	DURATION	NCT03345810	Non Small Cell Lung Cancer	Durvalumab	2	200	2017	S		*FACT-G*	FACT-L			clin trials	
62	EAGLE study	NCT02369874	squamous cell carcinoma of the head and neck	Durvalumab / Tremelimumab	3	736	2015	S	NR					NR	
63	ECHO-304/KEYNOTE-669	NCT03358472	Head and Neck Cancer	Pembrolizumab	3	89	2017	S	EQ-5D	EORTC QLQ-C30	QLQ-H&N35			protocol	https://clinicaltrials.gov/ProvidedDocs/72/NCT03358472/Prot_SAP_000.pdf
64	ECHO-305/KEYNOTE-654	NCT03322540	Lung Cancer	Pembrolizumab	2	154	2017	E	NR					NR	
65	ECHO-306/KEYNOTE-715	NCT03322566	Lung cancer	Pembrolizumab	2	233	2018	E	NR					NR	
66	EMERALD-2	NCT03847428	Hepatocellular Carcinoma	Durvalumab	3	888	2019	S	NR					NR	
67	ENGOT-EN9/LEAP-001	NCT03884101	Endometrial Neoplasms	Pembrolizumab	3	720	2019	S		EORTC QLQ-C30				clin trials	
68	EORTC 1325-MG/KEYNOTE-054	NCT02362594	Melanoma	Pembrolizumab	3	1019	2015	E	EQ-5D	EORTC QLQ-C30				abstract and protocol	https://clinicaltrials.gov/ProvidedDocs/94/NCT02362594/Prot_SAP_000.pdf
69	EORTC 18071	NCT00636168	Melanoma	Ipilimumab	3	1211	2008	S		EORTC QLQ-C30				conf abstract + clin trials + publication PRO results	https://ars.els-cdn.com/content/image/1-s2.0-S1470204515701221-mmc1.pdf
70	FORCE	NCT03044626	Non-Small-Cell Lung	Nivolumab	2	101	2017	S		*FACT-G*	FACT-L			clin trials	
71	GeparNuevo	NCT02685059	Breast Cancer	Durvalumab	2	174	2016	S		*FACT-G*			FACT-Taxane	EudraCT Number: 2015-002714-72	https://www.clinicaltrialsregister.eu/ctr-search/trial/2015-002714-72/DE#A
72	GETHI021	EudraCT2016-003946-99	pediatric solid tumors presenting in adulthood	Ipilimumab / Nivolumab			2017	S	NR					NR	
73	GHSG AERN	NCT03480334	Classical Hodgkin Lymphoma	Nivolumab	2	29	2019	S	NR					NR	
74	HAWK	NCT02207530	squamous cell carcinoma of the head and neck	Durvalumab	2	112	2014	S		EORTC QLQ-C30	QLQ-H&N35			clin trials	
75	HIMALAYA	NCT03298451	Hepatocellular Carcinoma	Durvalumab / Tremelimumab	3	1310	2017	E	NR					NR	
76	IFCT-1501 MAPS2	NCT02716272	Mesothelioma	Ipilimumab / Nivolumab	2	125	2016	S			LCSS			clin trials	
77	ILLUMINATE 301	NCT03445533	Metastatic Melanoma	ipilimumab	3	454	2018	S	NR					NR	
78	IMagyn050	NCT03038100	ovarian , fallopian tube, or primary peritoneal cancer	Atezolizumab	3	1300	2017	E		EORTC QLQ-C30	EORTC QLQ-OV28			clin trials	
79	IMbrave150	NCT03434379	Hepatocellular Carcinoma	Atezolizumab	3	480	2018	S		EORTC QLQ-C30				clin trials	
80	IMmotion150	NCT01984242	Renal Cell Carcinoma	Atezolizumab	2	305	2014	S	EQ-5D	MDASI		BFI		abstract + clin trials	
81	Immotion151	NCT02420821	Renal Cell Carcinoma	Atezolizumab	3	915	2017	S		MDASI		BFI	GP5-FKSI-19	clin trials	GP5 item FKSI-19 " *I am bothered by side effects of treatment*"
82	IMPACT	NCT03570619	metastatic prostate cancer	Ipilimumab / Nivolumab	2	40	2018	S	NR					NR	
83	IMpassion030	NCT03498716	Triple Negative Breast Cancer	Atezolizumab	3	2300	2018	S		EORTC QLQ-C30				clin trials	

	Trial	Trial ID	Condition / disease	Drug	Phase	Participants	Start	Endpoint	instruments					Source for PRO instrument identification	Comments
84	IMpassion031	NCT03197935	Triple-negative Breast Cancer	Atezolizumab	3	324	2017	S		EORTC QLQ-C30				clin trials	
85	IMpassion130	NCT02425891	Breast Cancer	Atezolizumab	3	900	2015	E	EQ-5D	EORTC QLQ-C30	QLQ-BR23			protocol	https://www.nejm.org/doi/suppl/10.1056/NEJMoa1809615/suppl_file/nejmoa1809615_protocol.pdf
86	IMpassion132	NCT03371017	Triple-negative Breast Cancer	Atezolizumab	3	540	2018	S		EORTC QLQ-C30				conf abstract + clin trials	
87	IMpower030	NCT03456063	Non-Small-Cell Lung	Atezolizumab	3	374	2018	S	GHS/HR QoL					clin trials	subscale (Questions 29 and 30) of the EORTC QLQ-C30
88	IMpower130	NCT02367781	Non-Small Cell Lung Cancer	Atezolizumab	3	724	2015	S		EORTC QLQ-C30	QLQ-LC13	SILC		clin trials	neither publication nor protocol available
89	IMpower131	NCT02367794	Non-Small Cell Lung Cancer	Atezolizumab	3	1021	2015	S		EORTC QLQ-C30	QLQ-LC13	SILC		clin trials	neither publication nor protocol available
90	IMpower132	NCT02657434	Non-Small Cell Lung Cancer	Atezolizumab	3	568	2016	S		EORTC QLQ-C30	QLQ-LC13			clin trials	neither publication nor protocol available
91	IMpower133	NCT02763579	Small Cell Lung Carcinoma	Atezolizumab	3	500	2016	S	EQ-5D	EORTC QLQ-C30	QLQ-LC13			clinicaltrials.gov + protocol + publication PRO results	https://www.nejm.org/doi/suppl/10.1056/NEJMoa1809064/suppl_file/nejmoa1809064_protocol.pdf
92	IMpower150	NCT02366143	Non-Small Cell Lung Cancer	Atezolizumab	3	1202	2015	S	EQ-5D	EORTC QLQ-C30	QLQ-LC13	SILC		clinicaltrials.gov + protocol	https://www.nejm.org/doi/suppl/10.1056/NEJMoa1716948/suppl_file/nejmoa1716948_protocol.pdf
93	IMspire 170	NCT03273153	advanced BRAFV600 wild-type melanoma	Atezolizumab/Pembrolizumab	3	450	2017	E	GHS/HR QoL						subscale (Questions 29 and 30) of the EORTC QLQ-C30
94	IMspire150	NCT02908672	Melanoma	Atezolizumab	3	513	2017	S		EORTC QLQ-C30				clin trials	
95	IMSTAR-HN	NCT03700905	Head and Neck Cancer	Ipilimumab / Nivolumab	3	276	2018	S		EORTC QLQ-C30	QLQ-H&N43			clin trials	
96	IMvoke010	NCT03452137	quamous Cell Carcinoma of the Head and Neck (SCCHN)	Atezolizumab	3	400	2018	S		EORTC QLQ-C30				clin trials	
97	JAVELIN bladder 100	NCT02603432	Urothelial Cancer	Avelumab	3	668	2016	S	EQ-5D	*FACT-G*	FACT-BL			clin trials	
98	JAVELIN Gastric 100	NCT02625610	Gastric cancer	Avelumab	3	499	2015	S	EQ-5D	EORTC QLQ-C30	QLQ-STO22			conf abstract + clinicaltrials.gov + protocol	https://www.futuremedicine.com/doi/10.2217/fon-2018-0668?url_ver=Z39.88-2003&rfr_id=ori:rid:crossref.org&rfr_dat=cr_pub%3dwww.ncbi.nlm.nih.gov
99	JAVELIN Gastric 300	NCT02625623	Gastric cancer	Avelumab	3	371	2015	S	EQ-5D	EORTC QLQ-C30	QLQ-STO22			Conf abstract + protocol + clin trials	https://clinicaltrials.gov/ProvidedDocs/23/NCT02625623/Prot_002.pdf
100	JAVELIN Lung 100	NCT02576574	Non-Small Cell Lung Cancer	Avelumab	3	1224	2015	S	EQ-5D	EORTC QLQ-C30	QLQ-LC13			conf abstract + clin trials	
101	Javelin Lung 200	NCT02395172	Non-Small Cell Lung Cancer	Avelumab	3	792	2015	S	EQ-5D	EORTC QLQ-C30	QLQ-LC13			conf abstract + clinicaltrials.gov + protocol	https://clinicaltrials.gov/ProvidedDocs/72/NCT02395172/Prot_001.pdf
102	JAVELIN Merkel 200	NCT02155647	Merkel cell carcinoma	Avelumab	2	204	2014	E	EQ-5D	FACT-G	FACT-M			publication PRO results	
103	JAVELIN Ovarian 100	NCT02718417	Ovarian Cancer	Avelumab	3	998	2016	S	EQ-5D		FOSI 18			clin trials	

	Trial	Trial ID	Condition / disease	Drug	Phase	Participants	Start	Endpoint	instruments					Source for PRO instrument identification	Comments
104	JAVELIN Ovarian 200	NCT02580058	Ovarian Cancer	Avelumab	3	566	2015	S	EQ-5D	EORTC QLQ-C30	QLQ-OV38	FOSI		conf abstract + clin trials	
105	JAVELIN Ovarian PARP100	NCT03642132	Ovarian Cancer	Avelumab	3	79	2018	S	EQ-5D		FOSI 18			clin trials (study discontinued)	https://clinicaltrials.gov/ct2/show/NCT03642132?term=NCT03642132&draw=2&rank=1
106	JAVELIN Renal 101	NCT02684006	Renal cell carcinoma	Avelumab	3	888	2016	S	EQ-5D		FKSI-19			clin trials	
107	KESTREL	NCT02551159	squamous cell carcinoma of the head and neck	Durvalumab / Tremelimumab	3	823	2015	S		EORTC QLQ-C30	QLQ-H&N35			clin trials	
108	KEYLYNK-010	NCT03834519	Prostatic Neoplasms	Pembrolizumab	3	780	2019	S				BPI-SF		clin trials	
109	KEYNOTE-002	NCT01704287	Melanoma	Pembrolizumab	2	540	2012	S	EQ-5D	EORTC QLQ-C30				conf abstract + protocol + publication PRO results	https://ars.els-cdn.com/content/image/1-s2.0-S1470204515000832-mmc1.pdf
110	KEYNOTE-006	NCT01866319	Melanoma	Pembrolizumab / Ipilimumab	3	834	2013	E	EQ-5D	EORTC QLQ-C30				conf abstract + protocol + publication PRO results	https://www.nejm.org/doi/suppl/10.1056/NEJMoa1503093/suppl_file/nejmoa1503093_protocol.pdf
111	KEYNOTE-010	NCT01905657	Non-small Cell Lung Cancer	Pembrolizumab	2/3	1061	2013	E	EQ-5D	EORTC QLQ-C30	QLQ-LC13			conf abstract + protocol + publication PRO results	https://ars.els-cdn.com/content/image/1-s2.0-S0140673615012817-mmc1.pdf
112	KEYNOTE-024	NCT02142738	Non-small Cell Lung Cancer	Pembrolizumab	3	305	2014	E		EORTC QLQ-C30	QLQ-LC13			conf abstract + protocol + publication PRO results	https://www.nejm.org/doi/suppl/10.1056/NEJMoa1606774/suppl_file/nejmoa1606774_protocol.pdf
113	KEYNOTE-033	NCT02864394	Non-Small-Cell Lung	Pembrolizumab	3	425	2016	E	NR					NR	
114	KEYNOTE-040	NCT02252042	Head and Neck Squamous Cell Cancer	Pembrolizumab	3	495	2014	E	EQ-5D	EORTC QLQ-C30	QLQ-H&N35			conf abstract + protocol	https://clinicaltrials.gov/ProvidedDocs/42/NCT02252042/Prot_SAP_000.pdf
115	KEYNOTE-045	NCT02256436	Urothelial Cancer	Pembrolizumab	3	542	2014	E	EQ-5D	EORTC QLQ-C30				conf abstract + publication PRO results	
116	KEYNOTE-057	NCT02625961	Bladder Cancer	Pembrolizumab	2	260	2016	E		*FACT-G*	FACT-BL			conf abstract	
117	KEYNOTE-061	NCT02370498	gastric or gastroesophageal junction adenocarcinoma	Pembrolizumab	3	592	2015	E	EQ-5D	EORTC QLQ-C30	QLQ-STO22			conf abstract + protocol	https://clinicaltrials.gov/ProvidedDocs/98/NCT02370498/Prot_SAP_000.pdf
118	KEYNOTE-062	NCT02494583	Gastric Adenocarcinoma	Pembrolizumab	3	763	2015	S		EORTC QLQ-C30	QLQ-STO22			conf abstract + clin trials	
119	KEYNOTE-087	NCT02453594	Hodgkin Lymphoma	Pembrolizumab	2	2011	2015	E	EQ-5D	EORTC QLQ-C30				protocol + publication PRO results	http://ascopubs.org/doi/suppl/10.1200/JCO.2016.72.1316/suppl_file/protocol_2016.721316.pdf
120	KEYNOTE-177	NCT02563002	Colorectal Carcinoma	Pembrolizumab	3	308	2015	E	NR					NR	
121	KEYNOTE-181	NCT02564263	Esophageal Carcinoma	Pembrolizumab	3	628	2015	E	EQ-5D	EORTC QLQ-C30	QLQ-OES18			conf abstract + protocol	https://clinicaltrials.gov/ProvidedDocs/63/NCT02564263/Prot_SAP_000.pdf
122	KEYNOTE-189	NCT02578680	Non-Small-Cell Lung Carcinoma	Pembrolizumab	3	616	2016	E	EQ-5D	EORTC QLQ-C30	QLQ-LC13			conf abstract + protocol	https://clinicaltrials.gov/ProvidedDocs/80/NCT02578680/Prot_SAP_000.pdf
123	KEYNOTE-240	NCT02702401	Hepatocellular Carcinoma	Pembrolizumab	3	413	2016	E	EQ-5D	EORTC QLQ-C30	QLQ-HCC18			conf abstract + protocol	https://clinicaltrials.gov/ProvidedDocs/01/NCT02702401/Prot_SAP_000.pdf

	Trial	Trial ID	Condition / disease	Drug	Phase	Participants	Start	Endpoint	instruments					Source for PRO instrument identification	Comments
124	KEYNOTE-407	NCT02775435	Non-small Cell Lung Cancer	Pembrolizumab	3	635	2016	E	EQ-5D	EORTC QLQ-C30	QLQ-LC13			conf abstract + protocol + publication PRO results	https://www.nejm.org/doi/suppl/10.1056/NEJMoa1810865/suppl_file/nejmoa1810865_protocol.pdf
125	KEYNOTE-412	NCT03040999	Head and Neck Neoplasms	Pembrolizumab	3	780	2017	S		EORTC QLQ-C30	QLQ-H&N35			clin trials	
126	KEYNOTE-426	NCT02853331	Renal cell carcinoma	Pembrolizumab	3	862	2016	S	NR					NR	
127	KEYNOTE-564	NCT03142334	Renal Cell Carcinoma	Pembrolizumab	3	950	2017	S		EORTC QLQ-C30	FKSI-DRS			clin trials	
128	KEYNOTE-581-CLEAR	NCT02811861	Renal Cell Carcinoma	Pembrolizumab	3	1069	2016	S	EQ-5D	EORTC QLQ-C30	FKSI-DRS			protocol	https://www.futuremedicine.com/doi/10.2217/fon-2018-0745?url_ver=Z39.88-2003&rfr_id=ori:rid:crossref.org&rfr_dat=cr_pub%3dwww.ncbi.nlm.nih.gov
129	KEYNOTE-590	NCT03189719	Esophageal Neoplasms	Pembrolizumab	3	749	2017	S	EQ-5D	EORTC QLQ-C30	QLQ-OES18			clin trials + protocol	https://www.futuremedicine.com/doi/10.2217/fon-2018-0609?url_ver=Z39.88-2003&rfr_id=ori%3Arid%3Acrossref.org&rfr_dat=cr_pub%3Dwww.ncbi.nlm.nih.gov&
130	KEYNOTE-604	NCT03066778	Small Cell Lung Cancer	Pembrolizumab	3	453	2017	S		EORTC QLQ-C30	QLQ-LC13			clin trials + protocol	
131	KEYNOTE-629	NCT03284424	Squamous Cell Carcinoma	Pembrolizumab	2	150	2017	E	NR					NR	
132	KEYNOTE-630	NCT03833167	Squamous Cell Carcinoma	Pembrolizumab	3	570	2019	S		EORTC QLQ-C30				clin trials	
133	KEYNOTE-671	NCT03425643	Non-small Cell Lung Cancer	Pembrolizumab	3	786	2018	S		EORTC QLQ-C30				clin trials	
134	KEYNOTE-672/ECHO-307	NCT03361865	Urothelial Cancer	Pembrolizumab	3	93	2017	S	EQ-5D	EORTC QLQ-C30				protocol	https://clinicaltrials.gov/ProvidedDocs/65/NCT03361865/Prot_SAP_000.pdf
135	KEYNOTE-676	NCT03711032	Bladder Cancer	Pembrolizumab	3	550	2018	S	EQ-5D	EORTC QLQ-C30	QLQ-NMIBC24			clin trials	
136	KEYNOTE-689	NCT03765918	Head and Neck Neoplasms	Pembrolizumab	3	704	2018	S		EORTC QLQ-C30	QLQ-H&N35			clin trials	
137	KEYNOTE-698/ECHO-303	NCT03374488	Urothelial Cancer	Pembrolizumab	3	84	2017	S	EQ-5D	EORTC QLQ-C30				protocol	https://clinicaltrials.gov/ProvidedDocs/88/NCT03374488/Prot_SAP_000.pdf
138	KEYNOTE-756	NCT03725059	Breast Cancer	Pembrolizumab	3	1140	2018	S		EORTC QLQ-C30	QLQ-BR23			clin trials	
139	KEYNOTE-775	NCT03517449	Endometrial Neoplasms	Pembrolizumab	3	780	2018	S		EORTC QLQ-C30				clin trials	
140	KEYNOTE-789	NCT03515837	Non-small Cell Lung Cancer	Pembrolizumab	3	480	2018	S		EORTC QLQ-C30	QLQ-LC13			conf abstract + clin trials	
141	KEYNOTE-826	NCT03635567	Cervical Cancer	Pembrolizumab	3	600	2018	S		EORTC QLQ-C30				clin trials	
142	KEYNOTE-866	NCT03924856	Bladder Cancer	Pembrolizumab	3	790	2019	S	EQ-5D	FACT-G	FACT-BI-Cys			clin trials	
143	KEYNOTE-905	NCT03924895	Bladder Cancer	Pembrolizumab	3	610	2019	E	NR					NR	

	Trial	Trial ID	Condition / disease	Drug	Phase	Participants	Start	Endpoint	instruments					Source for PRO instrument identification	Comments
144	KEYNOTE-937	NCT03867084	Hepatocellular Carcinoma	Pembrolizumab	3	950	2019	S	EQ-5D	EORTC QLQ-C30	QLQ-HCC18			clin trials	
145	LEAP-006	NCT03829319	Non-small Cell Lung Cancer	Pembrolizumab	3	726	2019	S		EORTC QLQ-C30	QLQ-LC13			clin trials	
146	LEAP-007	NCT03829332	Non-small Cell Lung Cancer	Pembrolizumab	3	620	2019	S		EORTC QLQ-C30	QLQ-LC13			clin trials	
147	LUD2013-006	NCT02336165	Glioblastoma	Durvalumab	2	159	2015	S		EORTC QLQ-C30	QLQ-BN20			conf abstract + clin trials	
148	LUNAR	NCT02973789	Non-small Cell Lung Cancer	Nivolumab / Pembrolizumab	3	534	2016	S		EORTC QLQ-C30	QLQ-LC13			conf abstract + clin trials	
149	MCC-17978	NCT02313272	Malignant Glioma	Pembrolizumab	1	32	2015	NR	NR					NR	
150	MDX010-20	NCT00094653	Melanoma	Ipilimumab	3	1783	2004	S	SF-36	EORTC QLQ-C30		SDS	FACIT-Fatigue Scale	conf abstract + protocol + publication PRO results	https://www.nejm.org/doi/suppl/10.1056/NEJMoa1003466/suppl_file/nejmoa1003466_protocol.pdf
151	MEDISARC AIO-STS 0415	NCT03317457	Adult Soft Tissue Sarcoma	Durvalumab / Tremelimumab	2	100	2017	S		EORTC QLQ-C30				ab + clin trials	
152	MEDITREME	NCT03202758	Colorectal Cancer	Durvalumab / Tremelimumab	1 / 2	48	2017	S	NR					NR	*secondary endpoints include overall response rate and quality of life.* Mentioned in conference abstract not in protoco*l* https://www.ncbi.nlm.nih.gov/pmc/articles/PMC6012564/
153	MYSTIC	NCT02453282	Non-Small Cell Lung Cancer	Durvalumab / Tremelimumab	3	1118	2015	S	NR					NR	
154	NCI-2016-00534	NCT02853318	ovarian, fallopian tube, or primary peritoneal cancer	Pembrolizumab	2	40	2016	NR	NR					NR	*"The primary objectives were to assess safety, clinical benefit, response rate, PFS, and quality of life"* but not in clin trials nor in WHO database http://apps.who.int/trialsearch/Trial3.aspx?trialid=NCT02853318
155	NeoTrio Trial	NCT02858921	Melanoma	Pembrolizumab	2	60	2017	S	NR					NR	
156	NIAGARA	NCT03732677	Bladder Cancer	Durvalumab	3	1050	2018	S / E	NR					NR	
157	NIBIT-M2	NCT02460068	Melanoma with brain metastasis	Ipilimumab / Nivolumab	3	168	2012	S	NR					NR	
158	NILE	NCT03682068	Urothelial Cancer	Durvalumab / Tremelimumab	3	885	2018	S	NR					NR	
159	NIVEAU trial	NCT03366272	Lymphoma, Non-Hodgkin	Nivolumab	2 / 3	388	2017	S	EQ-5D					conf abstract + clin trials	
160	NIVOSWITCH	NCT02959554	Renal Cell Carcinoma	Nivolumab	2	244	2016	S			FKSI-15			conf abstract + clin trials	
161	NRG LU005	NCT03811002	Limited Stage Lung Small Cell Carcinoma	Atezolizumab	2 / 3	506	2019	S	EQ-5D-5L	*FACT-G*	*FACT-L*	PROMIS Fatigue	PRO-CTCAE	clin trials	announced as FACT-TOI
162	NUTMEG	NCT04195139	Glioblastoma Multiforme	Nivolumab	2	102	2018	S	EQ-5D-5L	EORTC QLQ-C30	QLQ-BN-20			conf abstract + clin trials	
163	OAK	NCT02008227	Non-Small Cell Lung Cancer	Atezolizumab	3	1225	2014	S		EORTC QLQ-C30	QLQ-LC13			conf abstract+ clin trials + publication PRO results	
164	OpACIN	NCT02437279	Melanoma	Ipilimumab / Nivolumab	1	20	2015	NR	EORTC QLQ C30					conf abstract	

	Trial	Trial ID	Condition / disease	Drug	Phase	Participants	Start	Endpoint	instruments					Source for PRO instrument identification	Comments
165	OPTIM (AIO-KHT-0117)	NCT03620123	squamous carcinoma of the head and neck	Ipilimumab / Nivolumab	2	280	2018	S	EQ-5D-5L	EORTC QLQ-C30	QLQ-H&N35			clin trials	
166	ORION	NCT03775486	Non-small Cell Lung Cancer	Durvalumab	2	327	2018	S		EORTC QLQ-C30	QLQ-LC13			clin trials	
167	PACIFIC	NCT02125461	Non-Small Cell Lung Cancer	Durvalumab	3	713	2014	S	EQ-5D	EORTC QLQ-C30	QLQ-LC13			conf abstract + clin trials + publication PRO results	https://www.nejm.org/doi/suppl/10.1056/NEJMoa1709937/suppl_file/nejmoa1709937_protocol.pdf
168	PACIFIC-6	NCT03693300	Non-small Cell Lung Cancer	Durvalumab	2	150	2019	E	NR					NR	
169	PEARL	NCT03003962	Non-Small Cell Lung Cancer	Durvalumab	3	669	2017	S		EORTC QLQ-C30				clin trials	
170	PHAEDRA	ANZGOG1601	advanced endometrial cancer	Durvalumab	2	70	2017	S		EORTC QLQ-C30				ANZCTR	Trial registration ANZCTR
171	POLEM	NCT03827044	Colon Cancer	Avelumab	3	402	2018	S		EORTC QLQ-C30				clin trials	
172	POSEIDON	NCT03164616	Non-small Cell Lung Cancer	Durvalumab / Tremelimumab	3	1000	2017	S		EORTC QLQ-C30	QLQ-LC13			clin trials	
173	POSTCARD - GETUG-P13	EudraCT2017-003827-31	Prostate cancer	Durvalumab	2	96	2018	S		EORTC QLQ-C30	QLQ-PR25			clinical trials registry EU	
174	POTOMAC	NCT03528694	Bladder Cancer	Durvalumab	3	975	2018	S		EORTC QLQ-C30	QLQ-NMIBC24	pain with BPI + EVA	PRO-CTCAE	clin trials	
175	PRIMMO	NCT03192059	cervical, endometrial or uterine sarcoma	Pembrolizumab	2	43	2017	S		FACT-G	FACT-Cx			clin trials + protocol	https://bmccancer.biomedcentral.com/articles/10.1186/s12885-019-5676-3
176	PRISM	ISRCTN95351638	Renal Cancer	Ipilimumab / Nivolumab	2	189	2016	S	EQ-5D-5L	EORTC QLQ-C30	FKSI-19		EORTC item bank	protocol	https://bmccancer.biomedcentral.com/articles/10.1186/s12885-019-6273-1
177	PROSPER	NCT03055013	Renal Cancer	Nivolumab	3	805	2017	S			FKSI-19		PRO-CTCAE	clin trials	
178	PULSE	NCT03774901	Penile Cancer	Avelumab	2	32	2019	S		EORTC QLQ-C30				clin trials	
179	RAMONA	NCT03416244	Esophageal Cancer	Ipilimumab / Nivolumab	2	75	2018	S		EORTC QLQ-C30	QLQ-ELD14			clin trials + protocol	https://bmccancer.biomedcentral.com/articles/10.1186/s12885-019-5446-2
180	RTOG 3504	NCT02764593	Head and Neck Squamous Cell Carcinoma	Nivolumab	1	40	2016	S	NR					NR	
181	RTOG 3505	NCT02768558	Non-Small Cell Lung Cancer	Nivolumab	3	N/A	2016	S	EQ-5D-5L	*FACT-G*	FACT-L	PROMIS Fatigue		clin trials + protocol	https://clinicaltrials.gov/ProvidedDocs/58/NCT02768558/Prot_SAP_000.pdf
182	SH MISP203	NCT02586207	squamous cell carcinoma of the head and neck	Pembrolizumab	1	57	2015	S		*FACT-G*	FACT H&N			conf abstract + clin trials	neither publication nor protocol available
183	STERIMGLI	NCT02866747	Glioblastoma	Durvalumab	1 / 2	62	2017	S		EORTC QLQ-C30	QLQ-BN20			clin trials	
184	Study 1108	NCT01693562	Advanced Solid Tumors (mUC)	Durvalumab	1 / 2	1022	2012	E	EORTC QLQ-C30	*FACT-G*	FACT-BL	QLQ-cancer modules	pain (single item)	protocol + publication PRO results	https://jamanetwork.com/journals/jamaoncology/fullarticle/2648865

	Trial	Trial ID	Condition / disease	Drug	Phase	Participants	Start	Endpoint	instruments					Source for PRO instrument identification	Comments
185	SWOG - 1418/NRG BR-006	NCT02954874	Triple-Negative Breast Carcinoma	Pembrolizumab	3	1000	2016	P / S	PROMIS GPHS			PROMIS Fatigue	PRO-CTCAE	clin trials	
186	SWOG-1404	NCT02506153	Melanoma	Pembrolizumab	3	1378	2015	S	EQ-5D	*FACT-G*		FACT-BRM	FACIT-D	clin trials	
187	TEDOPaM-PRODIGE63	NCT03806309	Pancreatic Ductal Adenocarcinoma	Nivolumab	2	156	2019	S		EORTC QLQ-C30				conf abstract + clin trials	
188	TITAN-RCC	NCT02917772	Renal Cell Carcinoma	Ipilimumab / Nivolumab	2	200	2016	S			FKSI-19			clin trials	
189	UPCC 25514	NCT02316002	Oligometastatic Non-small Cell Lung Cancer	Pembrolizumab	2	51	2015	S		*FACT-G*	FACT-L			protocol	https://clinicaltrials.gov/ProvidedDocs/02/NCT02316002/Prot_SAP_000.pdf
190	VinMetAtezo	NCT03801304	Non-small Cell Lung Cancer	Atezolizumab	2	71	2019	S	EQ-5D	EORTC QLQ-C30				clin trials + protocol	https://www.futuremedicine.com/doi/10.2217/fon-2019-0730?url_ver=Z39.88-2003&rfr_id=ori:rid:crossref.org&rfr_dat=cr_pub%3dwww.ncbi.nlm.nih.gov
191	VLA-013 MITCI	NCT02307149	Melanoma	Pembrolizumab	1	59	2015	S	NR					NR	

Table S5: Cochrane checklist

**Article 1**	**KEYNOTE-087, pembrolizumab, Hodgkin Lymphoma**
**First author & year of publication**	**von Tresckow, 2019**
**Stated purpose of the article**	*we expand the report on the effect of pembrolizumab on patient-reported health-related quality of life (HRQoL) to include data up to week 24 and data on other HRQoL measures*
**What were PROs measuring?**	
What concepts were the PROs used in the study measuring?	HRQoL
What rationale (if any) for selection of concept or constructs did the authors provide?	Patient-reported outcome (PRO) assessment of the functional and symptomatic benefits of cancer treatment is increasingly used to complement clinical/biological data
Were patients involved in the selection of outcomes measured by the PROs?	Not reported
**Omissions**	
Were there any important aspects of health (e.g., symptoms, function, perceptions) or quality of life (e.g. overall evaluation, satisfaction with life) that were omitted in this study from the perspectives of the patient, clinician, significant others...	Treatment-related adverse events – any grade [1]
**If randomized trials and other studies measured PROs, what were the instruments' measurement strategies?**	
Did investigators use instruments that yield a single indicator or index number, a profile, or a battery or instruments?	Battery of instruments: EQ-5D; EORTC QLQ-C30
If investigators measure PROs, did they use specific or generic measures, or both?	Both: General health status and cancer-specific;
Who exactly completed the instruments?	all patients receiving the intended treatment
**Did the instruments work in the way they were supposed to work - validity?**	
Had the instruments used been validated previously (provide reference)? Was evidence of prior validation for use in this population presented?	*The QLQ-C30 is a cancer-specific, 30- item questionnaire found to be valid and reliable*<br>*The EQ-5D is a non-disease-specific measure of five health state*<br>*The cancer-specific QLQ-C30 instrument has undergone continuous development, refinement, and validation over decades, and a review of published cancer studies using EQ-5D has provided evidence to support its validity and reliability. Both have been used widely in HL clinical trials and other HL research study designs*<br>Aaronson NK, Ahmedzai S, Bergman B, et al. The European Organization for Research and Treatment of Cancer QLQ-C30: a quality-of-life instrument for use in international clinical trials in oncology. J Natl Cancer Inst. 1993<br>Fayers P, Bottomley A, EORTC Quality of Life Group, et al. Quality of life research within the EORTC QLQ-C30. European Organisation for Research and Treatment of Cancer. Eur J Cancer. 2002<br>Pickard AS, Neary MP, Cella D. Estimation of minimally important differences in EQ-5D utility and VAS scores in cancer. Health Qual Life Outcomes. 2007
Were the instruments re-validated in this study?	No
**Did the instruments work in the way they were supposed to work – ability to measure**	
Are the PROs able to detect change in patient status, even if those changes are small?	*Mean changes from baseline to week 24 showed improvements in QLQ-C30 functional and symptom domains in the FAS population and all three cohorts. Similar trends were also observed for QLQC30 at week 12 and for EQ-5D VAS at weeks 12 and 24*
**Can you make the magnitude of effect (if any) understandable to readers?**	
Can you provide an estimate of the difference in patients achieving a threshold of function or improvement, and the associated number needed to treat	**NA**

**Article 2**	**KEYNOTE-087; pembrolizumab, Hodgkin Lymphoma**
**First author & year of publication**	**Chen, 2017***[1]*<br>**The article presents the results of a phase III study including PROs as an exploratory outcome.*

NA: Not applicable

**Article 3**	**CheckMate 025 ; nivolumab vs everolimus; Renal cell carcinoma**
**First author & year of publication**	**Cella, 2016**
**Stated purpose of the article**	*We report on the complete HRQoL analysis, including results from mixed model analyses from CheckMate025*
**What were PROs measuring?**	
What concepts were the PROs used in the study measuring?	HRQoL; symptoms
What rationale (if any) for selection of concept or constructs did the authors provide?	*The main prespecified patient-reported outcome objective in the CheckMate 025 protocol was assessment of disease-related symptom progression in each treatment group based on the FKSI-DRS*
Were patients involved in the selection of outcomes measured by the PROs?	Not reported
**Omissions**	
Were there any important aspects of health (e.g., symptoms, function, perceptions) or quality of life (e.g. overall evaluation, satisfaction with life) that were omitted in this study from the perspectives of the patient, clinician, significant others...	Treatment-related adverse events – any grade [2]
**If randomized trials and other studies measured PROs, what were the instruments' measurement strategies?**	
Did investigators use instruments that yield a single indicator or index number, a profile, or a battery or instruments?	Battery: The Functional Assessment of Cancer Therapy-Kidney Symptom Index-Disease Related Symptoms (FKSI-DRS) *and* EQ-5D
If investigators measure PROs, did they use specific or generic measures, or both?	Both: Disease-specific related symptoms; General health status
Who exactly completed the instruments?	patients on-treatment phase from both treatment groups
**Did the instruments work in the way they were supposed to work - validity?**	
Had the instruments used been validated previously (provide reference)? Was evidence of prior validation for use in this population presented?	Cella D et al., Development and validation of a scale to measure disease-related symptoms of kidney cancer, 2007.<br>EuroQol Group, EuroQol- a new facility for the measurement of health-related quality of life,1990.<br>Dolan P. Modeling valuations EuroQol health states, 1997<br>Schrag A, et al. The EQ-5D-a generic quality of life measure-is a useful instrument to measure quality of life with patients with Parkinson's disease, 2000.
Were the instruments re-validated in this study?	No
**Did the instruments work in the way they were supposed to work – ability to measure**	
Are the PROs able to detect change in patient status, even if those changes are small?	Yes, significant mean difference between the two groups assessed by the FKSI-DRS. Scores for the EQ-5D utility index and VAS were higher in the *Nivolumab group compared with those from patients treated with everolimus.*<br>*Using FKSI-DRS, a clinically meaningful deterioration (p<0·001) in HRQoL was observed for patients receiving everolimus*<br>*For nivolumab versus everolimus, more patients experienced a clinically meaningful HRQoL improvement (p=0·001) assessed by EQ-5D VAS*
**Can you make the magnitude of effect (if any) understandable to readers?**	
Can you provide an estimate of the difference in patients achieving a threshold of function or improvement, and the associated number needed to treat	**NA**

**Article 4**	**CheckMate 067; Nivolumab *vs* Ipilimumab *vs* Nivolumab + Ipilimumab; Melanoma**
**First author & year of publication**	**Schadendorf, 2017**
**Stated purpose of the article**	*we report analyses of HRQoL for patients with advanced melanoma treated in CheckMate 067*
**What were PROs measuring?**	
What concepts were the PROs used in the study measuring?	HRQoL
What rationale (if any) for selection of concept or constructs did the authors provide?	*This toxicity profile might diminish health-related quality of life (HRQoL). Patient-reported outcomes (PROs), such as symptoms, HRQoL, and patient-perceived health status supplement clinical data and are now more important during decision-making in oncology because they provide a holistic understanding of patient experience and treatment effectiveness*
Were patients involved in the selection of outcomes measured by the PROs?	Not reported
**Omissions**	

Were there any important aspects of health (e.g., symptoms, function, perceptions) or quality of life (e.g. overall evaluation, satisfaction with life) that were omitted in this study from the perspectives of the patient, clinician, significant others...	Treatment-related adverse events – any grade [3]<br>*The instruments used to assess HRQoL are designed for patients treated with chemotherapy and may not detect the impact of the AEs observed with immunotherapy*
**If randomized trials and other studies measured PROs, what were the instruments' measurement strategies?**	
Did investigators use instruments that yield a single indicator or index number, a profile, or a battery or instruments?	battery of instruments: EQ-5D-3L and EQ-5D VAS, EORTC QLQ-C30
If investigators measure PROs, did they use specific or generic measures, or both?	Both: General health status; Cancer-specific
Who exactly completed the instruments?	all randomised patients
**Did the instruments work in the way they were supposed to work - validity?**	
Had the instruments used been validated previously (provide reference)? Was evidence of prior validation for use in this population presented?	Aaronson NK, et al. The European Organization for Research and Treatment of Cancer QLQ-C30: a quality-of-life instrument for use in international clinical trials in oncology. 1993<br>Scott NW, et al. EORTC QLQ-C30 reference values. 2008<br>EuroQol Group. EuroQol-a new facility for the measurement of health-related quality of life. 1990<br>Van Reenen M, oppe M. EQ-5D-3L user guide. Version 5.1. 2015<br>Reilly MC,et al. The validity and reproducibility of a work productivity and activity impairment instrument. 1993
Were the instruments re-validated in this study?	No
**Did the instruments work in the way they were supposed to work – ability to measure**	
Are the PROs able to detect change in patient status, even if those changes are small?	**EORTC QLQ-C30***: No clinically meaningful changes were observed in any treatment group while on treatment.*<br>*No clinically meaningful changes were observed in any group while on treatment in any time point* for **EQ-5D** utility index and for **EQ-5D VAS**<br>*In this study, HRQoL stayed within ranges defined as minimally important difference (MID) in the EORTC QLQ-C30, EQ-5D utility index, and EQ-5D VAS assessments, including across various subgroups of patients.*
**Can you make the magnitude of effect (if any) understandable to readers?**	
Can you provide an estimate of the difference in patients achieving a threshold of function or improvement, and the associated number needed to treat	**NA**

**Article 5**	**Checkmate 141; Nivolumab vs Investigator's choice; Squamous Cell Carcinoma of the Head and Neck**
**First author & year of publication**	**Harrington, 2017**
**Stated purpose of article**	*Report the full quality-of-life analysis based on three widely used, validated PRO questionnaires completed by patients in the CheckMate 141 study*
**What were PROs measuring?**	
What concepts were the PROs used in the study measuring?	Patient-reported quality-of-life outcomes
What rationale (if any) for selection of concept or constructs did the authors provide?	*Patient-reported outcomes (PROs) have been collected to assess quality of life in a small number of clinical trials of chemotherapy and targeted therapies in recurrent or metastatic squamous cell carcinoma of the head and neck, few of which have shown improvements or significant differences between treatment groups. However, baseline quality of life scores have been reported to be independent prognostic factors for overall survival in patients with recurrent or metastatic head and neck cancer. Therefore, there is a large unmet medical need for treatments that improve prognosis as well as preserve and maximise quality of life.*
Were patients involved in the selection of outcomes measured by the PROs?	Not reported
**Omissions**	
Were there any important aspects of health (e.g., symptoms, function, perceptions) or quality of life (e.g. overall evaluation, satisfaction with life) that were omitted in this study from the perspectives of the patient, clinician, significant others...	Treatment-related adverse events – any grade [4]<br>*Although the questionnaires used in this trial have been used previously in several clinical trials, their validation has been done primarily in patients with locally advanced disease; thus, it is possible that certain symptoms of importance in recurrent or metastatic squamous cell carcinoma of the head and neck could have been missed in this and other trials. Furthermore, the EQ-5D is a measure that can be used in general or targeted clinical populations, and is not apt to be as sensitive as a condition-targeted measure that is used in the designated population.*
**If randomized trials and other studies measured PROs, what were the instruments' measurement strategies?**	

Did investigators use instruments that yield a single indicator or index number, a profile, or a battery or instruments?	Battery of instruments: EORTC QLQ-C30; EORTC QLQ-H&N35; EQ-5D-3L and EQ-5D VAS
If investigators measure PROs, did they use specific or generic measures, or both?	Cancer-specific; Disease-specific; General health status
Who exactly completed the instruments?	patients under treatment and follow-up<br>EQ-5D also in patients which discontinued therapy
**Did the instruments work in the way they were supposed to work - validity?**	
Had the instruments used been validated previously (provide reference)? Was evidence of prior validation for use in this population presented?	*PRO assessments were done (…) using three validated patient-reported questionnaires the EQ-5D includes other measures that are important to patients with squamous cell carcinoma of the head and neck such as anxiety and depression, as well as measures not covered by the EORTC measures such as the ability to do general, daily activities.*<br>Mesia R, et al. Quality of life of patients receiving platinum-based chemotherapy plus cetuximab first line for recurrent and/or metastatic squamous cell carcinoma of the head and neck. 2010<br>Machiels JP, et al. Afatinib versus methotrexate as second-line treatment in patients with recurrent or metastatic squamous-cell carcinoma of the head and neck progressing on or after platinum-based therapy (LUX-Head & Neck 1): an open-label, randomised phase 3 trial. 2015<br>Bjordal K, et al. A 12 country field study of the EORTC QLQ-C30 (version 3.0) and the head and neck cancer specific module (EORTC QLQ-H&N35) in head and neck patients. EORTC Quality of Life Group. 2000<br>Bottomley A, et al. An international phase 3 trial in head and neck cancer: quality of life and symptom results: EORTC 24954 on behalf of the EORTC Head and Neck and the EORTC Radiation Oncology Group. 2014<br>Curran D, et al. Quality of life in head and neck cancer patients after treatment with high-dose radiotherapy alone or in combination with cetuximab. 2007<br>Sherman AC, et al. Assessing quality of life in patients with head and neck cancer: cross-validation of the European Organization for Research and Treatment of Cancer (EORTC) Quality of Life Head and Neck module (QLQ-H&N35). 2000<br>Pickard AS, et al. Estimation of minimally important differences in EQ-5D utility and VAS scores in cancer. 2007
Were the instruments re-validated in this study?	No
**Did the instruments work in the way they were supposed to work – ability to measure**	
Are the PROs able to detect change in patient status, even if those changes are small?	**EORTC QLQ-C30:** *Treatment with nivolumab resulted in adjusted mean changes from baseline to weeks 9 and 15 indicating no clinically meaningful changes. Clinically meaningful deterioration occurred in eight (53%) of the 15 domains in the investigator's choice group at week 15.*<br>**EORTC QLQ-H&N35:** *treatment with investigator's choice led to clinically meaningful deterioration (decline of 10 points or more) at week 15*<br>**EQ-5D VAS:** *patients in the nivolumab group had a clinically meaningful improvement (according to a difference of 7 points or greater) in adjusted mean change in VAS score from baseline to week 15, by contrast with a clinically meaningful deterioration in the investigator's choice group*<br>**EQ-5D:** *Neither significant nor clinically meaningful differences in outcomes were observed at 9 or 15 weeks within or between groups*
**Can you make the magnitude of effect (if any) understandable to readers?**	
Can you provide an estimate of the difference in patients achieving a threshold of function or improvement, and the associated number needed to treat	**NA**

**Article 6**	**Checkmate 141; Nivolumab *vs* Investigator's choice; Squamous Cell Carcinoma of the Head and Neck**
**First author & year of publication**	**Ferris, 2016** *[4]*<br>**The article presents the results of a phase III study including PROs as an exploratory outcome.*

**Article 7**	**EORTC-18071; Ipilimumab vs Placebo; Melanoma**
**First author & year of publication**	**Coens, 2017**
**Stated purpose of article**	*we report the HRQoL data from the EORTC 18071 trial. To compare health-related quality-of-life (HRQoL) outcomes between the two treatment groups using the EORTC QLQ-C30 quality-of-life instrument*
**What were PROs measuring?**	
What concepts were the PROs used in the study measuring?	health-related quality-of-life (HRQoL) outcomes
What rationale (if any) for selection of concept or constructs did the authors provide?	*A pre-specified secondary endpoint of this study was to compare health-related quality-of-life (HRQoL) outcomes between the two treatment groups*
Were patients involved in the selection of outcomes measured by the PROs?	Not reported

**Omissions**	
Were there any important aspects of health (e.g., symptoms, function, perceptions) or quality of life (e.g. overall evaluation, satisfaction with life) that were omitted in this study from the perspectives of the patient, clinician, significant others...	Treatment-related adverse events – any grade [5]<br>*Although the EORTC QLQ-C30 questionnaire is one of the most commonly used and validated measures within the oncology clinical trials setting, when applied to this particular study, no immunotherapy-specific validation exists, and several symptoms common to immune-related adverse events are missing. Most notably absent are symptoms related to endocrine (hypothyroiditis, hypophysitis) or skin reactions, which can be significant burdens for the patient that are not always clinically apparent.*
**If randomized trials and other studies measured PROs, what were the instruments' measurement strategies?**	
Did investigators use instruments that yield a single indicator or index number, a profile, or a battery or instruments?	Single instrument: EORTC QLQ-C30
If investigators measure PROs, did they use specific or generic measures, or both?	cancer-specific
Who exactly completed the instruments?	All randomized patients
**Did the instruments work in the way they were supposed to work - validity?**	
Had the instruments used been validated previously (provide reference)? Was evidence of prior validation for use in this population presented?	*This patient-reported-outcome tool* [EORTC QLQ-C30] *is composed of 30 questions, measuring various aspects of HRQoL that are specific to cancer.*<br>*the EORTC QLQ-C30 core questionnaire has been successfully used to detect clinically relevant treatment differences in a melanoma patient population*<br>Aaronson NK, et al. The European Organization for Research and Treatment of Cancer QLQ-C30: a quality-of-life instrument for use in international clinical trials in oncology. 1993<br>De Wolf L, et al. EORTC translating procedures. Brussels: EORTC Publications, 2009.<br>Bottomley A, et al. Adjuvant therapy with pegylated interferon alfa-2b versus observation in resected stage III melanoma: a phase III randomized controlled trial of health-related quality of life and symptoms by the European Organisation for Research and Treatment of Cancer Melanoma Group. 2009
Were the instruments re-validated in this study?	No
**Did the instruments work in the way they were supposed to work – ability to measure**	
Are the PROs able to detect change in patient status, even if those changes are small?	*ipilimumab had no significant effect on global health status after induction.*<br>*Significantly worse outcomes in the ipilimumab group compared with the placebo group were found for specific symptom scales—namely, diarrhoea, insomnia, and fatigue*<br>*Ipilimumab can be administered in this patient population without clinically relevant deterioration in HRQoL as measured by the EORTC QLQ-C30 questionnaire.*
**Can you make the magnitude of effect (if any) understandable to readers?**	
Can you provide an estimate of the difference in patients achieving a threshold of function or improvement, and the associated number needed to treat	**NA**

**Article 8**	**KEYNOTE-002; Pembrolizumab vs Chemotherapy; Melanoma**
**First author & year of publication**	**Schadendorf, 2016**
**Stated purpose of article**	*To report the analyses of HRQoL for patients with advanced melanoma treated with pembrolizumab compared with investigator's choice of chemotherapy in KEYNOTE-002*
**What were PROs measuring?**	
What concepts were the PROs used in the study measuring?	Health Related Quality of Life (HRQoL)
What rationale (if any) for selection of concept or constructs did the authors provide?	*Although outcomes for cancer patients are generally measured in terms of survival and response, patient- reported outcomes (PROs) and health-related quality of life (HRQoL) are of high relevance to the patient*
Were patients involved in the selection of outcomes measured by the PROs?	Not reported
**Omissions**	
Were there any important aspects of health (e.g., symptoms, function, perceptions) or quality of life (e.g. overall evaluation, satisfaction with life) that were omitted in this study from the perspectives of the patient, clinician, significant others...	Treatment-related adverse events – any grade [6]<br>*Clinical trial populations may differ from melanoma patients in the general population with regard to motivation, the likelihood of PRO reporting, and ability to withstand treatment-related AEs.*

**If randomized trials and other studies measured PROs, what were the instruments' measurement strategies?**	
Did investigators use instruments that yield a single indicator or index number, a profile, or a battery or instruments?	Single instrument: EORTC QLQ-C30
If investigators measure PROs, did they use specific or generic measures, or both?	cancer-specific
Who exactly completed the instruments?	All randomized patients
**Did the instruments work in the way they were supposed to work - validity?**	
Had the instruments used been validated previously (provide reference)? Was evidence of prior validation for use in this population presented?	Cormier JN, et al. Assessment of patient-reported outcomes in patients with melanoma. 2011
Were the instruments re-validated in this study?	No
**Did the instruments work in the way they were supposed to work – ability to measure**	
Are the PROs able to detect change in patient status, even if those changes are small?	*Consistently smaller proportions of deteriorated and larger proportions of stable or improved GHS/HRQoL and functional and symptoms scales scores were observed for the two pembrolizumab arms compared with the chemotherapy arm.*<br>*GHS/HRQoL deteriorated by ≥10 points in 7-12% fewer patients in the pembrolizumab arms than in the chemotherapy arm between baseline and week 12*
**Can you make the magnitude of effect (if any) understandable to readers?**	
Can you provide an estimate of the difference in patients achieving a threshold of function or improvement, and the associated number needed to treat	**NA**

**Article 9**	**MDX010-20; Ipilimumab with or without gp100 vaccine; Melanoma**
**First author & year of publication**	**Revicki, 2012**
**Stated purpose of article**	*To summarize the HRQL outcomes during the 12 week treatment induction period of the ipilimumab Phase III clinical trial (MDX010-20)*
**What were PROs measuring?**	
What concepts were the PROs used in the study measuring?	Health Related Quality of Life
What rationale (if any) for selection of concept or constructs did the authors provide?	*Assessment of the effects of ipilimumab in relation to overall HRQL is important and will allow oncologists to appropriately educate patients on the risks and benefits of treatment with this agent*<br>Extensive evidence is available supporting the reliability, validity, and responsiveness of the EORTC QLQ-C30 in different cancer populations<br>*We identified three studies that used the EORTC QLQ-C30 comparing treatments for advanced melanoma*
Were patients involved in the selection of outcomes measured by the PROs?	Not reported
**Omissions**	
Were there any important aspects of health (e.g., symptoms, function, perceptions) or quality of life (e.g. overall evaluation, satisfaction with life) that were omitted in this study from the perspectives of the patient, clinician, significant others...	Treatment-related adverse events – any grade [7]<br>*Although EORTC QLQ-C30 is an internationally validated, widely used questionnaire for assessing the HRQL in oncology, melanoma specific HRQL questions might not have been addressed.*
**If randomized trials and other studies measured PROs, what were the instruments' measurement strategies?**	
Did investigators use instruments that yield a single indicator or index number, a profile, or a battery or instruments?	Single instrument; EORTC QLQ-C30
If investigators measure PROs, did they use specific or generic measures, or both?	cancer-specific
Who exactly completed the instruments?	All randomized patients
**Did the instruments work in the way they were supposed to work - validity?**	
Had the instruments used been validated previously (provide reference)? Was evidence of prior validation for use in this population presented?	Aaronson NK, et al. The European Organizationfor Research and Treatment of Cancer QLQ-C30: a quality-of-life instrument for use in international clinical trials in oncology. 1993<br>Osoba D, Modification of the EORTC QLQ-C30 (version 2.0) based on content validity and reliability testing in large samples of patients with cancer. The Study Group on Quality of Life of the EORTC and the Symptom Control and Quality of Life Committees of the NCI of Canada Clinical Trials Group. 1997<br>Avril MF, et al. Fotemustine compared with dacarbazine in patients with disseminated malignant melanoma: a phase III study. 2004

	Kiebert GM, et al. Health-related quality of life in patients with advanced metastatic melanoma: results of a randomized phase III study comparing temozolomide with dacarbazine. 2003<br>Sigurdardottir V, et al. Quality of life evaluation by the EORTC questionnaire technique in patients with generalized malignant melanoma on chemotherapy.1996
Were the instruments re-validated in this study?	No
**Did the instruments work in the way they were supposed to work – ability to measure**	
Are the PROs able to detect change in patient status, even if those changes are small?	the PROs did detect changes and the authors reported the changes as no change, minimal or moderate<br>*The HRQL results for the ipilimumab groups demonstrate that ipilimumab treatment is associated with minimal impairments on functioning and symptoms during the treatment induction period. The only statistically significant difference between ipilimumab and gp100 vaccine was for constipation, and this finding may be due to increased rate of colitis in the ipilimumab groups*
**Can you make the magnitude of effect (if any) understandable to readers?**	
**Can you provide an estimate of the difference in patients achieving a threshold of function or improvement, and the associated number needed to treat**	**NA**

**Article 10**	**KEYNOTE-006; Pembrolizumab Q2W vs Pembrolizumab Q3W vs Ipilimumab; Melanoma**
**First author & year of publication**	**Petrella, 2017**
**Stated purpose of article**	*To report results of patient-reported health-related quality of life (HRQoL) and symptoms from phase III KEYNOTE-006 study of pembrolizumab versus ipilimumab in patients with ipilimumab-naive advanced melanoma.*
**What were PROs measuring?**	
What concepts were the PROs used in the study measuring?	HRQoL and symptoms
What rationale (if any) for selection of concept or constructs did the authors provide?	*Patient-reported outcomes (PROs) are increasingly used as a complement to biological data to inform pa- tient-centred care and clinical decision-making. Indeed, research has indicated that health-related quality of life (HRQoL) impairment may affect the survival rate of patients with melanoma*
Were patients involved in the selection of outcomes measured by the PROs?	Not reported
**Omissions**	
Were there any important aspects of health (e.g., symptoms, function, perceptions) or quality of life (e.g. overall evaluation, satisfaction with life) that were omitted in this study from the perspectives of the patient, clinician, significant others...	Treatment-related adverse events – any grade [8]
**If randomized trials and other studies measured PROs, what were the instruments' measurement strategies?**	
Did investigators use instruments that yield a single indicator or index number, a profile, or a battery or instruments?	battery of instruments: EQ-5D-3L and EQ-5D VAS; EORTC QLQ-C30
If investigators measure PROs, did they use specific or generic measures, or both?	Both: General health status, Cancer-specific
Who exactly completed the instruments?	All randomized patients
**Did the instruments work in the way they were supposed to work - validity?**	
Had the instruments used been validated previously (provide reference)? Was evidence of prior validation for use in this population presented?	Refers to the HRQoL results of the following studies: EORTC 18071, CheckMate 067, Keynote-002, measured by the EORTC QLQ-C30<br>Aaronson NK, et al. The European Organization for Research and Treatment of Cancer QLQ-C30: a quality-of-life instrument for use in international clinical trials in oncology. J Natl Cancer Inst 1993<br>The EuroQol Group. EuroQolea new facility for the measure- ment of health-related quality of life. Health Policy 1990
Were the instruments re-validated in this study?	No
**Did the instruments work in the way they were supposed to work – ability to measure**	
Are the PROs able to detect change in patient status, even if those changes are small?	*In the two pembrolizumab arms, the least squares mean for EORTC QLQ-C30 GHS/QoL score changes from baseline to week 12 were minimal. For the ipilimumab arm, there was a clinically meaningful -10.0 point change*<br>*A statistically significant difference in the EQ-5D utility scores was observed between both pembrolizumab arms and the ipilimumab arm. No differences in the results of EORTC QLQ-C30 and EQ-5D were observed between the two pembrolizumab schedules.*
**Can you make the magnitude of effect (if any) understandable to readers?**	

Can you provide an estimate of the difference in patients achieving a threshold of function or improvement, and the associated number needed to treat	**NA**

**Article 11**	**KEYNOTE-024; Pembrolizumab vs Chemotherapy; Non-small-cell lung cancer**
**First author & year of publication**	**Brahmer, 2017**
**Stated purpose of article**	*We report the effect of pembrolizumab versus chemotherapy on patient-reported outcomes (PROs), evaluated as prespecified exploratory endpoints, in the KEYNOTE-024 study.*
**What were PROs measuring?**	
What concepts were the PROs used in the study measuring?	HRQoL and symptoms
What rationale (if any) for selection of concept or constructs did the authors provide?	*Patients with advanced NSCLC have a high burden of symptoms, such as fatigue, cough, dyspnoea, anorexia, weight loss, and pain that can have a substantial negative effect on health-related quality of life (HRQOL) and functioning. Therefore, the effect of novel treatments on symptom control and HRQOL needs to be considered alongside survival.*
Were patients involved in the selection of outcomes measured by the PROs?	Not reported
**Omissions**	
Were there any important aspects of health (e.g., symptoms, function, perceptions) or quality of life (e.g. overall evaluation, satisfaction with life) that were omitted in this study from the perspectives of the patient, clinician, significant others...	Treatment-related adverse events – any grade [9]<br>*The use of QOL instruments that were developed in the chemotherapy era, and might thus not adequately reflect the experiences of patients receiving therapies developed since then, could also be considered a limitation. However, although the QLQ-C30 and QLQ-LC13 questionnaires were not developed specifically for use with immunotherapies, they do remain in large part applicable because they capture key symptoms across both treatment groups (eg, fatigue and gastrointestinal symptoms). Immunotherapy-associated symptoms (eg, pruritus, pneumonitis) are not considered by these instruments, but were captured by clinicians using Common Terminology Criteria for Adverse Events version 4.0*
**If randomized trials and other studies measured PROs, what were the instruments' measurement strategies?**	
Did investigators use instruments that yield a single indicator or index number, a profile, or a battery or instruments?	Battery of instruments: EORTC QLQ-C30; EORTC QLQ-LC13; EQ-5D-3L and EQ-5D VAS
If investigators measure PROs, did they use specific or generic measures, or both?	Cancer-specific; Disease-specific; General health
Who exactly completed the instruments?	All randomized patients
**Did the instruments work in the way they were supposed to work - validity?**	
Had the instruments used been validated previously (provide reference)? Was evidence of prior validation for use in this population presented?	*The QLQ-C30 and QLQ-LC13 questionnaires have been validated for use in patients with a variety of cancer diagnoses, including lung cancer. The EQ-5D-3L instrument is used to calculate a health index score, and a visual analogue scale that patients use to rate their current general health status (GHS)*<br>Aaronson NK, et al. The European Organization for Research and Treatment of Cancer QLQ-C30: a quality-of-life instrument for use in international clinical trials in oncology. 1993<br>Osoba D, et al. Interpreting the significance of changes in health-related quality-of-life scores. 1998<br>Bergman B, et al. The EORTC QLQ-LC13: a modular supplement to the EORTC Core Quality of Life Questionnaire (QLQ-C30) for use in lung cancer clinical trials. EORTC Study Group on Quality of Life. 1994<br>Hjermstad MJ, et al. Test/retest study of the European organization for Research and Treatment of Cancer Core Quality-of-Life Questionnaire. 1995<br>Osoba D, et al. Psychometric properties and responsiveness of the EORTC quality of life questionnaire (QLQ-C30) in patients with breast, ovarian and lung cancer. 1994<br>Ringdal GI, et al. Testing the EORTC Quality of Life Questionnaire on cancer patients with heterpgenous diagnoses. 1993<br>Velikova G, et al. Health-related Quality of Life in EORTC clinical trials-30 years of progress from methodological developments to making a real impact on oncology practice. 2017.
Were the instruments re-validated in this study?	No
**Did the instruments work in the way they were supposed to work – ability to measure**	
Are the PROs able to detect change in patient status, even if those changes are small?	*Pembrolizumab was associated with a clinically meaningful improvement in HRQOL compared with that for platinum-based chemotherapy as first-line treatment in patient with metastatic NSCLC and a PD-L1 tumour proportion score of 50% or more.*<br>*At 15 weeks, GHS/QOL score on the QLQ-C30 for pembrolizumab was significantly different from that for chemotherapy.*<br>*No significant difference was found in EQ-5D-3L visual analogue scale score by week 15 between the two treatment groups*
**Can you make the magnitude of effect (if any) understandable to readers?**	

Can you provide an estimate of the difference in patients achieving a threshold of function or improvement, and the associated number needed to treat	**NA**

**Article 12**	**CheckMate 066; Nivolumab vs Dacarbazine; Melanoma**
**First author & year of publication**	**Long, 2016**
**Stated purpose of article**	*Herein are presented results of prospectively collected analyses in CheckMate 066 that compared the impact of nivolumab and dacarbazine on HRQoL using reliable and validated patient-reported outcomes (PROs).*
**What were PROs measuring?**	
What concepts were the PROs used in the study measuring?	HRQoL
What rationale (if any) for selection of concept or constructs did the authors provide?	*A frequent concern with immunotherapies is that their toxicity profile might diminish health-related quality of life (HRQoL), even when meaningful disease outcomes are observed. Given the increasing importance of considering HRQoL during treatment decision-making in oncology, the CheckMate 066 study incorporated these measures.*
Were patients involved in the selection of outcomes measured by the PROs?	Not reported
**Omissions**	
Were there any important aspects of health (e.g., symptoms, function, perceptions) or quality of life (e.g. overall evaluation, satisfaction with life) that were omitted in this study from the perspectives of the patient, clinician, significant others...	Treatment-related adverse events – any grade [10]
**If randomized trials and other studies measured PROs, what were the instruments' measurement strategies?**	
Did investigators use instruments that yield a single indicator or index number, a profile, or a battery or instruments?	battery of instruments: EQ-5D-3L and EQ-5D VAS; EORTC QLQ-C30
If investigators measure PROs, did they use specific or generic measures, or both?	Both: General health; Cancer-specific
Who exactly completed the instruments?	all randomized patients during on-study and follow-up phases
**Did the instruments work in the way they were supposed to work - validity?**	
Had the instruments used been validated previously (provide reference)? Was evidence of prior validation for use in this population presented?	*HRQoL was evaluated using the EORTC QLQ-C30 and the EQ-5D-3L, two PROs whose use has been well documented in advanced melanoma. The study used PROs that were not developed and validated specifically for melanoma. However, given their common use in melanoma clinical trials, these instruments were considered to be the most content valid among those currently available*<br>Cornish D, et al. A systematic review of health-related quality of life in cutaneous melanoma. 2009<br>Askew RL, et al. Mapping FACT-melanoma quality-of-life scores to EQ-5D health utility weights. 2011.<br>Revicki DA, et al. Health related quality of life outcomes for unresectable stage III or IV melanoma patients receiving ipilimumab treatment. 2012<br>Grob JJ, et al. Patient perception of the benefit of a BRAF inhibitor in metastatic melanoma: quality-of-life analyses of the BREAK-3 study comparing dabrafenib with dacarbazine. 2014<br>Schadendorf D, et al. Functional and symptom impact of trametinib versus chemotherapy in BRAF V600E advanced or metastatic melanoma: quality-of-life analyses of the METRIC study. 2014<br>Schadendorf D, et al. Health-related quality of life impact in a randomised phase III study of the combination of dabrafenib and trametinib versus dabrafenib monotherapy in patients with BRAF V600 metastatic melanoma. 2015<br>Schadendorf D, et al.Patient-reported outcomes (PROs) in KEYNOTE-002, a randomized study of pembrolizumab vs chemotherapy in patients (pts) with ipilimumab-refractory (IPI-R) metastatic melanoma (MEL). 2015, ASCO Poster 9040.
Were the instruments re-validated in this study?	No
**Did the instruments work in the way they were supposed to work – ability to measure**	
Are the PROs able to detect change in patient status, even if those changes are small?	In general, both **EORTC QLQ-C30** *functioning subscale and symptom mean scores remained relatively stable over time compared with baseline for both groups, with a few statistically significant and clinically meaningful changes.*<br>**EQ-5D:** *The only significant difference observed between treatment arms was at week 7. Clinically meaningful improvements occurred in patients treated with nivolumab.*<br>**EQ-5D VAS:** *Significant improvements from baseline were observed for patients receiving nivolumab. Clinically meaningful improvements were noted for nivolumab patients. No significant or clinically meaningful improvements from baseline were observed for dacarbazine patients.*
**Can you make the magnitude of effect (if any) understandable to readers?**	

Can you provide an estimate of the difference in patients achieving a threshold of function or improvement, and the associated number needed to treat	**NA**

**Article 13**	**KEYNOTE-010; Pembrolizumab vs Docetaxel; PD-L1–Expressing NSCLC**
**First author & year of publication**	**Barlesi, 2019**
**Stated purpose of article**	The effects of pembrolizumab and docetaxel on HRQoL were evaluated in the present study
**What were PROs measuring?**	
What concepts were the PROs used in the study measuring?	HRQoL
What rationale (if any) for selection of concept or constructs did the authors provide?	*In addition to efficacy, the tolerability of treatment and impact on health-related quality of life (HRQoL) are very important considerations for patients with cancer.*
Were patients involved in the selection of outcomes measured by the PROs?	Not reported
**Omissions**	
Were there any important aspects of health (e.g., symptoms, function, perceptions) or quality of life (e.g. overall evaluation, satisfaction with life) that were omitted in this study from the perspectives of the patient, clinician, significant others...	Treatment-related adverse events – any grade [11]
**If randomized trials and other studies measured PROs, what were the instruments' measurement strategies?**	
Did investigators use instruments that yield a single indicator or index number, a profile, or a battery or instruments?	Battery of instruments: EQ-5D-3L; EORTC QLQ-C30 and EORTC-LC13
If investigators measure PROs, did they use specific or generic measures, or both?	Both: global health status; time to deterioration; composite endpoint of cough; dyspnea; chest pain
Who exactly completed the instruments?	*The HRQoL instruments were administered electronically by trained personnel and completed by the patients themselves: all randomized patients who received at least one dose of study medication*
**Did the instruments work in the way they were supposed to work - validity?**	
Had the instruments used been validated previously (provide reference)? Was evidence of prior validation for use in this population presented?	*The effects of pembrolizumab and docetaxel on HRQoL were evaluated in the present study using three patient- reported outcomes (PRO) instruments that have been widely used in phase III NSCLC trials*<br>Aaronson NK, et al. The European Organization for Research and Treatment of Cancer QLQ-C30: a quality-of-life instrument for use in international clinical trials in oncology. J Natl Cancer Inst. 1993.<br>Bergman B, et al. The EORTC QLQ-LC13: a modular supplement to the EORTC Core Quality of Life Questionnaire (QLQ- C30) for use in lung cancer clinical trials. EORTC Study Group on Quality of Life. Eur J Cancer. 1994.<br>The EuroQol Group. EuroQol—a new facility for the measurement of health-related quality of life. Health Policy. 1990.<br>Brooks R. EuroQol: the current state of play. Health Policy. 1996<br>Shaw AT, et al Crizotinib versus chemotherapy in advanced ALK-positive lung cancer. N Engl J Med. 2013.<br>Blackhall F, et al. Patient-reported outcomes and quality of life in PROFILE 1007: a randomized trial of crizotinib compared with chemotherapy in previously treated patients with ALK-positive advanced non-small-cell lunch cancer. J Thorac Oncol. 2014
Were the instruments re-validated in this study?	**No**
**Did the instruments work in the way they were supposed to work – ability to measure**	
Are the PROs able to detect change in patient status, even if those changes are small?	*findings suggest that HRQoL and symptoms were either maintained or improved to a greater degree with 2 mg/kg pembrolizumab than with docetaxel in this population of patients with previously treated, PD-L1–expressing (TPS >=1%) advanced NSCLC*
**Can you make the magnitude of effect (if any) understandable to readers?**	
Can you provide an estimate of the difference in patients achieving a threshold of function or improvement, and the associated number needed to treat	**NA**

**Article 14**	**OAK; Atezolizumab vs Docetaxel ; Advanced Non-Small-cell Lung Cancer**
**First author & year of publication**	**Bordoni, 2018**

**Stated purpose of article**	*PRO data were collected to determine the relative effect of atezolizumab and docetaxel on symptom burden, patient functioning, and HRQoL in patients with previously treated, advanced NSCLC*
**What were PROs measuring?**	
What concepts were the PROs used in the study measuring?	Symptom burden, patient functioning, and HRQoL
What rationale (if any) for selection of concept or constructs did the authors provide?	*In lung cancer, worsening of cancer-related symptoms, not only adversely affects patients' HRQoL, but has also been shown to correlate with a lower response to treatment and might be associated with reduced OS. Similarly, research has shown that both physical functioning and patient-reported pain were predictive of survival and could be considered collectively as part of the evaluation of a treatment's benefit/risk profile in NSCLC.*
Were patients involved in the selection of outcomes measured by the PROs?	Not reported
**Omissions**	
Were there any important aspects of health (e.g., symptoms, function, perceptions) or quality of life (e.g. overall evaluation, satisfaction with life) that were omitted in this study from the perspectives of the patient, clinician, significant others...	Treatment-related adverse events – any grade [12]
**If randomized trials and other studies measured PROs, what were the instruments' measurement strategies?**	
Did investigators use instruments that yield a single indicator or index number, a profile, or a battery or instruments?	Battery: EORTC-QLQ-C30 and EORTC-LC13
If investigators measure PROs, did they use specific or generic measures, or both?	Cancer-specific; Disease-specific
Who exactly completed the instruments?	patients
**Did the instruments work in the way they were supposed to work - validity?**	
Had the instruments used been validated previously (provide reference)? Was evidence of prior validation for use in this population presented?	*The PROs reflecting lung cancer symptoms, commonly reported treatment-related symptoms (eg, nausea, constipation), functioning in daily life, and HRQoL were collected using 2 self-administered questionnaires that have been routinely used in lung cancer studies*<br>Aaronson NK, et al. The European Organization for Research and Treatment of Cancer QLQ-C30: a quality of life instrument for use in international clinical trials in oncology. J Natl Cancer Inst 1993<br>Bergman B, et al. The EORTC QLQ-LC13: a modular supplement to the EORTC core quality of life questionnaire (QLQ-C30) for use in lung cancer clinical trials. EORTC Study Group on Quality of Life. Eur J Cancer 1994
Were the instruments re-validated in this study?	No
**Did the instruments work in the way they were supposed to work – ability to measure**	
Are the PROs able to detect change in patient status, even if those changes are small?	*PRO data from the phase III OAK study have indicated that atezolizumab prolongs the time until patients with advanced NSCLC experience limitations in performing their day-to-day activities (physical function and role function) and improves patient HRQoL compared with docetaxel*
**Can you make the magnitude of effect (if any) understandable to readers?**	
Can you provide an estimate of the difference in patients achieving a threshold of function or improvement, and the associated number needed to treat	**NA**

**Article 15**	**PACIFIC; Durvalumab after chemoradiotherapy; stage III, unresectable NSCLC**
**First author & year of publication**	**Hui, 2019**
**Stated purpose of article**	*Phase 3 PACIFIC trial, durvalumab improved the primary endpoints of progression-free survival and overall survival compared with that for placebo, with similar safety, in patients with unresectable, stage III non-small-cell lung cancer. In this analysis, we aimed to evaluate one of the secondary endpoints, patient-reported outcomes (PROs).*
**What were PROs measuring?**	
What concepts were the PROs used in the study measuring?	patients' symptoms, functioning, and global health status (ie, quality of life)
What rationale (if any) for selection of concept or constructs did the authors provide?	*Patient-reported outcomes (PROs), which reflect patients' perspective of their symptoms, functioning, and health-related quality of life, can provide important complementary data to efficacy and safety endpoints.*
Were patients involved in the selection of outcomes measured by the PROs?	Not reported
**Omissions**	

Were there any important aspects of health (e.g., symptoms, function, perceptions) or quality of life (e.g. overall evaluation, satisfaction with life) that were omitted in this study from the perspectives of the patient, clinician, significant others...	Treatment-related adverse events – any grade [13]
**If randomized trials and other studies measured PROs, what were the instruments' measurement strategies?**	
Did investigators use instruments that yield a single indicator or index number, a profile, or a battery or instruments?	Battery: EORTC-QLQ-C30 and EORTC-LC13
If investigators measure PROs, did they use specific or generic measures, or both?	patient-reported symptoms, functioning, and global health status or quality of life
Who exactly completed the instruments?	All randomized patients
**Did the instruments work in the way they were supposed to work - validity?**	
Had the instruments used been validated previously (provide reference)? Was evidence of prior validation for use in this population presented?	*QLQ-C30 was tested in interviews of patients with lung cancer during development of the disease-specific lung cancer module. Both QLQ-C30 and QLQ-LC13 are widely used in patients with advanced non-small-cell lung cancer, and they have been well validated in psychometric testing.*<br>Aaronson NK, et al. The European Organization for Research and Treatment of Cancer QLQ-C30: a quality-of-life instrument for use in international clinical trials in oncology. J Natl Cancer Inst. 1993.<br>Bergman B, et al. The EORTC QLQ-LC13: a modular supplement to the EORTC Core Quality of Life Questionnaire (QLQ- C30) for use in lung cancer clinical trials. EORTC Study Group on Quality of Life. Eur J Cancer. 1994.
Were the instruments re-validated in this study?	**No**
**Did the instruments work in the way they were supposed to work – ability to measure**	
Are the PROs able to detect change in patient status, even if those changes are small?	*The between-group differences in changes from baseline to 12 months in cough (difference in adjusted mean changes 1·1, 95% CI –1·89 to 4·11), dyspnoea (1·6, –0·58 to 3·87), chest pain (0·4, –2·13 to 2·93), fatigue (2·2,–0·38 to 4·78), appetite loss (1·2, –1·27 to 3·67), physical functioning (–1·9, –3·91 to 0·15), or global health status or quality of life (0·8, –1·55 to 3·14) were not clinically relevant. Generally, there were no clinically important between group differences in time to deterioration of pre-specified key PRO endpoints.*
**Can you make the magnitude of effect (if any) understandable to readers?**	
Can you provide an estimate of the difference in patients achieving a threshold of function or improvement, and the associated number needed to treat	**NA**

**Article 16**	**JAVELIN Merkel 200; avelumab; metastatic Merkel cell carcinoma**
**First author & year of publication**	**Kaufman, 2018**
**Stated purpose of article**	*To assess the association between tumor response and health-related quality of life (HRQoL) in patients with metastatic Merkel cell carcinoma treated with the anti-PD-L1 avelumab*
**What were PROs measuring?**	
What concepts were the PROs used in the study measuring?	HRQoL
What rationale (if any) for selection of concept or constructs did the authors provide?	To date, there is little knowledge about quality of life (QoL) and the impact of disease progression in patients with mMCC
Were patients involved in the selection of outcomes measured by the PROs?	Not reported
**Omissions**	
Were there any important aspects of health (e.g., symptoms, function, perceptions) or quality of life (e.g. overall evaluation, satisfaction with life) that were omitted in this study from the perspectives of the patient, clinician, significant others...	Treatment-related adverse events – any grade [14]<br>*The majority of adverse events experienced under treatment with avelumab (including grade 3 or 4 adverse events) do not have a detrimental impact on HRQoL.*
**If randomized trials and other studies measured PROs, what were the instruments' measurement strategies?**	
Did investigators use instruments that yield a single indicator or index number, a profile, or a battery or instruments?	HRQoL was assessed with the melanoma-specific FACT-M questionnaire and the generic 5-level EQ-5D (EQ5D-5L) questionnaire
If investigators measure PROs, did they use specific or generic measures, or both?	General health; Cancer-specific; disease specific
Who exactly completed the instruments?	

**Did the instruments work in the way they were supposed to work - validity?**	
Had the instruments used been validated previously (provide reference)? Was evidence of prior validation for use in this population presented?	*Among generic instruments, the EuroQol – 5 Dimensions (EQ-5D) questionnaire has been widely used in oncology and has been shown to be reliable and responsive and to have content as well as construct validity.*<br>*No disease-specific instruments exist to specifically capture health-related quality of life (HRQoL) in patients with MCC. However, the Functional Assessment of Cancer Therapy – Melanoma (FACT-M) has shown promising characteristics for patients with malignant melanomas*<br>Schwenkglenks M, et al. Is the EQ-5D suitable for use in oncology? An overview of the literature and recent developments. Expert Rev. Pharmacoecon. Outcomes Res. (2016).<br>Cormier JN, Cromwell KD, Ross MI. Health-related quality of life in patients with melanoma: overview of instruments and outcomes. Dermatol. Clin. (2012).<br>EuroQol – a new facility for the measurement of health-related quality of life. Health Policy 16(3), 199–208 (1990).<br>Herdman M, Gudex C, Lloyd A et al. Development and preliminary testing of the new five-level version of EQ-5D (EQ-5D-5L). Qual. Life Res. 2011
Were the instruments re-validated in this study?	**Yes**, FACT-M for the MCC population<br>*the content of the FACT-M seems appropriate to assess QoL in patients with MCC. This was further confirmed by a thorough psychometric validation of the FACT-M in the JAVELIN Merkel 200 trial population*<br>Bharmal M, Fofana F, Dias Barbosa C, Mahnke L, Schlichting M. Psychometric validation of the FACT-M questionnaire in patients with Merkel cell carcinoma. Presented at: *ISPOR 22nd Annual International Meeting.* Abstract PCN189, MA, USA, 20–24 May 2017
**Did the instruments work in the way they were supposed to work – ability to measure**	
Are the PROs able to detect change in patient status, even if those changes are small?	*Results show that reduction in tumor size correlates with improvements in patients' health status, as measured by both generic and skin cancer specific HRQoL instruments. Results also demonstrated significantly higher HRQoL and utility for patients with non-PD compared with PD, measured as per RECIST criteria. Clinically meaningful differences were observed for most FACT-M changes from baseline scores.*
**Can you make the magnitude of effect (if any) understandable to readers?**	
Can you provide an estimate of the difference in patients achieving a threshold of function or improvement, and the associated number needed to treat	**NA**

**Article 17**	**IMpower133; Atezolizumab, carboplatin, and etoposide; SCLC**
**First author & year of publication**	**Mansfield, 2019**
**Stated purpose of article**	*We have evaluated adverse events (AEs) and patient-reported outcomes in IMpower133 to assess the benefit-risk profile of this regimen.*<br>*Here we report the safety profile of atezolizumab combined with CP/ET in the induction and maintenance settings, and the impact of treatment on symptoms, functioning, and HRQoL from the patient's perspective, to inform overall treatment burden.*
**What were PROs measuring?**	
What concepts were the PROs used in the study measuring?	symptoms, functioning, and HRQoL
What rationale (if any) for selection of concept or constructs did the authors provide?	*In assessing the overall benefiterisk profile of a new treatment regimen, particularly in a non-curative setting, it is important to consider the impact of disease and/or treatment burden on patients' safety and health-related quality of life (HRQoL), to ensure that the benefits of enhanced tumor control and increased survival do not come at the expense of increased toxicity and reduced HRQoL.*
Were patients involved in the selection of outcomes measured by the PROs?	Not reported
**Omissions**	
Were there any important aspects of health (e.g., symptoms, function, perceptions) or quality of life (e.g. overall evaluation, satisfaction with life) that were omitted in this study from the perspectives of the patient, clinician, significant others...	Treatment-related adverse events – any grade [15]
**If randomized trials and other studies measured PROs, what were the instruments' measurement strategies?**	
Did investigators use instruments that yield a single indicator or index number, a profile, or a battery or instruments?	Battery: EORTC QLQ-C30 and QLQ-LC13
If investigators measure PROs, did they use specific or generic measures, or both?	Both: cancer-specific and disease specific
Who exactly completed the instruments?	**Patients**
**Did the instruments work in the way they were supposed to work - validity?**	

Had the instruments used been validated previously (provide reference)? Was evidence of prior validation for use in this population presented?	Aaronson NK, Ahmedzai S, Bergman B, et al. The European Organization for Research and Treatment of Cancer QLQ-C30: a quality of life instrument for use in international clinical trials in oncology. J Natl Cancer Inst 1993; 85:365-76<br>Bergman B, Aaronson NK, Ahmedzai S, Kaasa S, Sullivan M. The EORTC QLQ-LC13: a modular supplement to the EORTC core quality of life questionnaire (QLQ-C30) for use in lung cancer clinical trials. EORTC Study Group on Quality of Life. Eur J Cancer 1994; 30A:635-42
Were the instruments re-validated in this study?	
**Did the instruments work in the way they were supposed to work – ability to measure**	
Are the PROs able to detect change in patient status, even if those changes are small?	*Considering the broader impact of symptoms on patients' global health status, while HRQoL improved in both arms, clinically meaningful improvements persisted in the atezolizumab plus CP/ET arm through week 54, suggesting that the survival benefit achieved with the addition of atezolizumab to CP/ET was associated with minimal impact on treatment-related symptoms. Taken together, the notable HRQoL improvements reported by patients in the atezolizumab arm suggest that the addition of atezolizumab to CP/ ET did not increase toxicity or symptom burden.*
**Can you make the magnitude of effect (if any) understandable to readers?**	
Can you provide an estimate of the difference in patients achieving a threshold of function or improvement, and the associated number needed to treat	**NA**

**Article 18**	**KEYNOTE-407; Carboplatin-Paclitaxel or nab-Paclitaxel with or without Pembrolizumab; Metastatic Squamous NSCLC**
**First author & year of publication**	**Mazieres, 2019**
**Stated purpose of article**	*We report patient-reported outcomes (PROs) from KEYNOTE-407, which were evaluated as prespecified exploratory endpoints.*
**What were PROs measuring?**	
What concepts were the PROs used in the study measuring?	GHS/QoL and time to deterioration in lung cancer symptoms
What rationale (if any) for selection of concept or constructs did the authors provide?	*Disease-related symptoms associated with advanced NSCLC are associated with poor health-related quality of life (HRQoL)*
Were patients involved in the selection of outcomes measured by the PROs?	Not reported
**Omissions**	
Were there any important aspects of health (e.g., symptoms, function, perceptions) or quality of life (e.g. overall evaluation, satisfaction with life) that were omitted in this study from the perspectives of the patient, clinician, significant others...	Treatment-related adverse events – any grade [16]
**If randomized trials and other studies measured PROs, what were the instruments' measurement strategies?**	
Did investigators use instruments that yield a single indicator or index number, a profile, or a battery or instruments?	Battery: EQ-5D-3L, EORTC QLQ-C30, and EORTC QLQ-LC13
If investigators measure PROs, did they use specific or generic measures, or both?	General health status, cancer-specific, disease-specific
Who exactly completed the instruments?	**patients**
**Did the instruments work in the way they were supposed to work - validity?**	
Had the instruments used been validated previously (provide reference)? Was evidence of prior validation for use in this population presented?	Aaronson NK, Ahmedzai S, Bergman B, et al. The European Organization for Research and Treatment of Cancer QLQ-C30: a quality of life instrument for use in international clinical trials in oncology. J Natl Cancer Inst 1993; 85:365-76<br>Bergman B, Aaronson NK, Ahmedzai S, Kaasa S, Sullivan M. The EORTC QLQ-LC13: a modular supplement to the EORTC core quality of life questionnaire (QLQ-C30) for use in lung cancer clinical trials. EORTC Study Group on Quality of Life. Eur J Cancer 1994; 30A:635-42
Were the instruments re-validated in this study?	No
**Did the instruments work in the way they were supposed to work – ability to measure**	
Are the PROs able to detect change in patient status, even if those changes are small?	*HRQoL results favoring the pembrolizumab-combination group at week 18 were supported by trends in the EORTC functional scales, which favored the pembrolizumab combination group across all scales at weeks 9 and 18.*<br>*The largest differences occurred in physical functioning (abilities essential for maintaining independence) and role functioning (abilities for work/leisure). Improvements in physical and role functioning are relevant, given the anticipated continued increase in cancer survivorship; treatment regimens may differentially affect patient functioning, and physical and role functional status is likely to affect HRQoL. The symptom scale results include trends favoring each treatment group.*
**Can you make the magnitude of effect (if any) understandable to readers?**	

Can you provide an estimate of the difference in patients achieving a threshold of function or improvement, and the associated number needed to treat	**NA**

**Article 19**	**CheckMate 358; Nivolumab; Recurrent or Metastatic Cervical, Vaginal, or Vulvar Carcinoma**
**First author & year of publication**	**Naumann, 2019**<br>**The article presents the results of a phase I/II study including PROs as an exploratory outcome.*
**Stated purpose of article**	*Nivolumab was assessed in patients with virus-associated tumors in the phase I/II CheckMate 358 trial. We report on patients with recurrent/metastatic cervical, vaginal, or vulvar cancers. Exploratory end points included safety and patient-reported outcomes (PROs)*
**What were PROs measuring?**	
What concepts were the PROs used in the study measuring?	cancer-specific health-related quality of life and overall health status
What rationale (if any) for selection of concept or constructs did the authors provide?	Not Reported
Were patients involved in the selection of outcomes measured by the PROs?	Not reported
**Omissions**	
Were there any important aspects of health (e.g., symptoms, function, perceptions) or quality of life (e.g. overall evaluation, satisfaction with life) that were omitted in this study from the perspectives of the patient, clinician, significant others...	Treatment-related adverse events – any grade [17]
**If randomized trials and other studies measured PROs, what were the instruments' measurement strategies?**	
Did investigators use instruments that yield a single indicator or index number, a profile, or a battery or instruments?	Battery: EQ-5D and EORTC QLQ-C30
If investigators measure PROs, did they use specific or generic measures, or both?	Cancer-specific and general health status
Who exactly completed the instruments?	Patients undergoing treatment
**Did the instruments work in the way they were supposed to work - validity?**	
Had the instruments used been validated previously (provide reference)? Was evidence of prior validation for use in this population presented?	Not Reported
Were the instruments re-validated in this study?	**No**
**Did the instruments work in the way they were supposed to work – ability to measure**	
Are the PROs able to detect change in patient status, even if those changes are small?	*Patients were stable at week 9 compared with baseline on the basis of EQ-5D utility scores and EQ-5D visual analog scale scores. PRO data were not reported for the vaginal/vulvar cohort owing to small patient numbers*<br>*On the basis of EORTC QLQ-C30 scores, global health status was stable at week 9 compared with baseline, as were physical functioning, emotional functioning, and social functioning; however, clinically meaningful deterioration was noted in role functioning and cognitive functioning. Patients reported clinically meaningful improvement in pain and constipation, clinically meaningful deterioration in fatigue, and stability with regard to nausea and vomiting, dyspnea, insomnia, appetite loss, diarrhea, and financial difficulties.*
**Can you make the magnitude of effect (if any) understandable to readers?**	
Can you provide an estimate of the difference in patients achieving a threshold of function or improvement, and the associated number needed to treat	**NA**

**Article 20**	**Study 1108; Durvalumab; Advanced/Metastatic Urothelial Carcinoma**
**First author & year of publication**	**O'Donnell, 2019**<br>**The article presents the results of PRO and biomarkers in a a phase I/II study*
**Stated purpose of article**	Here, in a post hoc analysis of Study 1108, we report the impact of durvalumab on PROs, and we assess the relationship between inflammatory biomarkers and PROs.

**What were PROs measuring?**	
What concepts were the PROs used in the study measuring?	Disease-related symptoms, functioning, and HRQOL
What rationale (if any) for selection of concept or constructs did the authors provide?	As novel treatments for mUC emerge, it is equally important to evaluate changes in disease-related symptoms, functioning, and HRQOL with patient-reported outcome (PRO) measures along- side efficacy and safety data.
Were patients involved in the selection of outcomes measured by the PROs?	No
**Omissions**	
Were there any important aspects of health (e.g., symptoms, function, perceptions) or quality of life (e.g. overall evaluation, satisfaction with life) that were omitted in this study from the perspectives of the patient, clinician, significant others...	Treatment-related adverse events – any grade [18]
**If randomized trials and other studies measured PROs, what were the instruments' measurement strategies?**	
Did investigators use instruments that yield a single indicator or index number, a profile, or a battery or instruments?	Battery: Functional Assessment of Cancer Therapy–Bladder (FACT-Bl), EORTC QLQ-C30, and a single-item pain questionnaire
If investigators measure PROs, did they use specific or generic measures, or both?	Cancer-specific, disease-specific, symptom-specific (single item)
Who exactly completed the instruments?	patients
**Did the instruments work in the way they were supposed to work - validity?**	
Had the instruments used been validated previously (provide reference)? Was evidence of prior validation for use in this population presented?	Supporting information file:<br>Cella D. FACIT Manual: Manual of the Functional Assessment of Chronic Illness Therapy (FACIT) Scales. 4th ed. Evanston, Illinois: Evanston Northwestern Healthcare and Northwestern University; 1997.<br>Aaronson NK, Ahmedzai S, Bergman B, et al. The European Organization for Research and Treatment of Cancer QLQ-C30: a quality-of-life instrument for use in international clinical trials in oncology. *J Natl Cancer Inst*. 1993;85:365-376.
Were the instruments re-validated in this study?	**No**
**Did the instruments work in the way they were supposed to work – ability to measure**	
Are the PROs able to detect change in patient status, even if those changes are small?	*Improvements in scores over time were seen with FACT-Bl total scores, FACT-Bl BLCS, and FACT-Bl TOI together with improvements in the EORTC QLQ-C30 functional domains, Global Health Status score, and symptom scores (Pain and Fatigue). HRQOL improvements in patients with stable disease do not represent a response, but they do signify a potential clinical benefit.*
**Can you make the magnitude of effect (if any) understandable to readers?**	
Can you provide an estimate of the difference in patients achieving a threshold of function or improvement, and the associated number needed to treat	**NA**

**Article 21**	**CheckMate 017; Nivolumab versus Docetaxel ; Advanced Squamous NSCLC**
**First author & year of publication**	**Reck, 2017**
**Stated purpose of article**	*In the phase III CheckMate 017 study, nivolumab prolonged overall survival versus docetaxel in previously treated patients with advanced squamous NSCLC. Study objectives included health-related quality of life (HRQoL) and symptom assessments.*
**What were PROs measuring?**	
What concepts were the PROs used in the study measuring?	Health-Related Quality of Life and Symptoms
What rationale (if any) for selection of concept or constructs did the authors provide?	*Assessment of patient-reported outcomes (PROs) in cancer trials is valuable for understanding in- dividual symptom benefits, impact on HRQoL, and the balance of potential risks with treatment.*<br>*Few studies have reported on HRQoL in the second-line NSCLC setting, and none has evaluated pa- tients with the squamous histologic type specifically.*
Were patients involved in the selection of outcomes measured by the PROs?	Not Reported
**Omissions**	
Were there any important aspects of health (e.g., symptoms, function, perceptions) or quality of life (e.g. overall evaluation,	Treatment-related adverse events – any grade [19]

satisfaction with life) that were omitted in this study from the perspectives of the patient, clinician, significant others...	
**If randomized trials and other studies measured PROs, what were the instruments' measurement strategies?**	
Did investigators use instruments that yield a single indicator or index number, a profile, or a battery or instruments?	Battery: LCSS, EQ-SD utility index; EQ-5D VAS
If investigators measure PROs, did they use specific or generic measures, or both?	General health status and disease-specific
Who exactly completed the instruments?	Patients
**Did the instruments work in the way they were supposed to work - validity?**	
Had the instruments used been validated previously (provide reference)? Was evidence of prior validation for use in this population presented?	HRQoL was analyzed by using the validated, patient-reported Lung Cancer Symptom Scale (LCSS) and the European Quality of Life Five Dimensions (EQ-5D) questionnaires.<br>Hollen PJ, Gralla RJ, Kris MG, Potanovich LM. Quality of life assessment in individuals with lung cancer: testing the Lung Cancer Symptom Scale (LCSS). Eur J Cancer. 1993<br>Hollen PJ, et al. Measurement of quality of life in patients with lung cancer in multicenter trials of new therapies. Psychometric assessment of the Lung Cancer Symptom Scale. Cancer. 1994<br>Hollen PJ, Gralla RJ, Kris MG, Cox C. Quality of life during clinical trials: conceptual model for the Lung Cancer Symptom Scale (LCSS). Support Care Cancer. 1994<br>Pickard AS, Neary MP, Cella D. Estimation of minimally important differences in EQ-5D utility and VAS scores in cancer. Health Qual Life Outcomes. 2007<br>EuroQol Group. EuroQol—a new facility for the mea- surement of health-related quality of life. Health Policy. 1990
Were the instruments re-validated in this study?	**No**
**Did the instruments work in the way they were supposed to work – ability to measure**	
Are the PROs able to detect change in patient status, even if those changes are small?	*Although disease-related symptom improvement was similar with nivolumab and docetaxel at the prespecified 12-week analysis, clear improvements with nivolumab were evident at later time points, which may be related to its distinct mechanism of action. These conclusions are supported by data from two PRO instruments and a multistep analytical plan evaluating data descriptively, cross-sectionally, and longitudinally.*
**Can you make the magnitude of effect (if any) understandable to readers?**	
Can you provide an estimate of the difference in patients achieving a threshold of function or improvement, and the associated number needed to treat	**NA**

**Article 22**	**CheckMate 227; Nivolumab plus ipilimumab versus chemotherapy ; advanced NSCLC**
**First author & year of publication**	**Reck, 2019**
**Stated purpose of article**	*To evaluate patient-reported outcomes (PROs) in this population.*
**What were PROs measuring?**	
What concepts were the PROs used in the study measuring?	Disease-related symptoms and general health status
What rationale (if any) for selection of concept or constructs did the authors provide?	High symptom burden of patients with non-NSCLC
Were patients involved in the selection of outcomes measured by the PROs?	Not Reported
**Omissions**	
Were there any important aspects of health (e.g., symptoms, function, perceptions) or quality of life (e.g. overall evaluation, satisfaction with life) that were omitted in this study from the perspectives of the patient, clinician, significant others...	Treatment-related adverse events – any grade [20]
**If randomized trials and other studies measured PROs, what were the instruments' measurement strategies?**	
Did investigators use instruments that yield a single indicator or index number, a profile, or a battery or instruments?	Battery: Lung Cancer Symptom Scale(LCSS), EQ-5D
If investigators measure PROs, did they use specific or generic measures, or both?	General health status and disease-specific

Who exactly completed the instruments?	Patients
**Did the instruments work in the way they were supposed to work - validity?**	
Had the instruments used been validated previously (provide reference)? Was evidence of prior validation for use in this population presented?	*PROs were assessed using two validated measures, the Lung Cancer Symptom Scale (LCSS) to examine the impact of treatment on lung cancer-specific symptoms and the EQ-5D to examine the impact of treatment on general health status.*<br>Hollen, PJ, Gralla RJ, Kris MG, Cox C. Quality of life during clinical trials: conceptual model for the Lung Cancer Symptom Scale (LCSS). Support Care Canc 1994; 2(4): 213-22<br>Hollen, PJ, et al. Measurement of quality of life in patients with lung cancer in multicentre trails of new therapies. Psychometric assessment of the Lung Cancer Symptom Scale. Cancer 1994; 73(8):2087-98.<br>Hollen, PJ, Gralla RJ, Kris MG, Potanovich LM. Quality of life assessment in individuals with lung cancer: testing the Lung Cancer Symptoms Scale (LCSS). Eur J Cancer 199329A(Suppl 1):S51-8.<br>Rabin R, de Charro F. EQ-5D: a measure of health status from the EuroQoL Group. Ann Med 2001;33(5):337-43.
Were the instruments re-validated in this study?	**No**
**Did the instruments work in the way they were supposed to work – ability to measure**	
Are the PROs able to detect change in patient status, even if those changes are small?	*patients treated with nivolumab plus ipilimumab experienced more rapid, durable and clinically meaningful improvements in PROs than those treated with chemotherapy.*
**Can you make the magnitude of effect (if any) understandable to readers?**	
Can you provide an estimate of the difference in patients achieving a threshold of function or improvement, and the associated number needed to treat	**NA**

**Article 23**	**CheckMate 153; Nivolumab; Advanced Non–Small Cell Lung Cancer**
**First author & year of publication**	**Spigel, 2019**<br>**The article presents the results of a phase IIIB/IV study including PROs as an exploratory outcome.*
**Stated purpose of article**	*we report safety, efficacy, and patient-reported outcome (PRO) results from CheckMate 153 for the entire patient population, patients aged 70 years or older, and those with an Eastern Cooperative Oncology Group performance status (ECOG PS) of 2.*
**What were PROs measuring?**	
What concepts were the PROs used in the study measuring?	General QoL, symptom burden
What rationale (if any) for selection of concept or constructs did the authors provide?	Not Reported
Were patients involved in the selection of outcomes measured by the PROs?	Not Reported
**Omissions**	
Were there any important aspects of health (e.g., symptoms, function, perceptions) or quality of life (e.g. overall evaluation, satisfaction with life) that were omitted in this study from the perspectives of the patient, clinician, significant others...	Treatment-related adverse events – any grade [21]
**If randomized trials and other studies measured PROs, what were the instruments' measurement strategies?**	
Did investigators use instruments that yield a single indicator or index number, a profile, or a battery or instruments?	Battery: EQ-5D and LCSS
If investigators measure PROs, did they use specific or generic measures, or both?	Generic health status; disease-specific
Who exactly completed the instruments?	**patients**
**Did the instruments work in the way they were supposed to work - validity?**	
Had the instruments used been validated previously (provide reference)? Was evidence of prior validation for use in this population presented?	EuroQoL Research Foundation. EQ-5D-3L user guide. Version 5.1. April 2015. https://euroqol.org/wpcontent/uploads/2016/09/EQ-5D-3L_UserGuide_2015.pdf<br>Bharmal M, Thomas J 3rd. Comparing the EQ-5D and the SF-6D descriptive systems to assess their ceiling effects in the US general population. Value Health. 2006<br>Pickard AS, Neary MP, Cella D. Estimation of minimally important differences in EQ-5D utility and VAS scores in cancer. Health Qual Life Outcomes. 2007

	Hollen PJ, Gralla RJ, Kris MG, et al. Measurement of quality of life in patients with lung cancer in multicenter trials of new therapies. Psychometric assessment of the Lung Cancer Symptom Scale. Cancer. 1994
Were the instruments re-validated in this study?	
**Did the instruments work in the way they were supposed to work – ability to measure**	
Are the PROs able to detect change in patient status, even if those changes are small?	*Patients' symptom burden (as measured by the LCSS average symptom burden index) decreased with length of time receiving treatment; the mean change from baseline in symptoms decreased continually for patients still receiving treatment and approached the clinically meaningful threshold of a mean change from baseline of more than 10 points. Very similar patterns of improved quality of life and decreased symptom burden were also observed in patients aged 70 years or older.*<br>*Patients with an ECOG PS of 2 had a lower quality of life and greater symptom burden at baseline than did the overall population; however, these patients also demonstrated improvements in quality of life and decreases in symptoms while undergoing treatment*
**Can you make the magnitude of effect (if any) understandable to readers?**	
Can you provide an estimate of the difference in patients achieving a threshold of function or improvement, and the associated number needed to treat	**NA**

**Article 24**	**KEYNOTE-045; Pembrolizumab versus Chemotherapy; Urothelial cancer**
**First author & year of publication**	**Vaughn, 2018**
**Stated purpose of article**	*In the phase III KEYNOTE-045 study, pembrolizumab significantly prolonged overall survival compared with investigator's choice of chemotherapy in patients with previously treated advanced urothelial cancer. Here, we report the results of health related quality-of-life (HRQoL) analyses from the KEYNOTE-045 trial.*
**What were PROs measuring?**	
What concepts were the PROs used in the study measuring?	HRQoL
What rationale (if any) for selection of concept or constructs did the authors provide?	Patients with urothelial cancer, in particular, report significant and clinically relevant decrements across all HRQoL domains, with the greatest difficulties related to fatigue and social and role functioning
Were patients involved in the selection of outcomes measured by the PROs?	Not Reported
**Omissions**	
Were there any important aspects of health (e.g., symptoms, function, perceptions) or quality of life (e.g. overall evaluation, satisfaction with life) that were omitted in this study from the perspectives of the patient, clinician, significant others...	Treatment-related adverse events – any grade [22]
**If randomized trials and other studies measured PROs, what were the instruments' measurement strategies?**	
Did investigators use instruments that yield a single indicator or index number, a profile, or a battery or instruments?	EQ-5D and EORTC QLQ-C30
If investigators measure PROs, did they use specific or generic measures, or both?	Generic health status and cancer-specific
Who exactly completed the instruments?	patients
**Did the instruments work in the way they were supposed to work - validity?**	
Had the instruments used been validated previously (provide reference)? Was evidence of prior validation for use in this population presented?	Not Reported
Were the instruments re-validated in this study?	**No**
**Did the instruments work in the way they were supposed to work – ability to measure**	
Are the PROs able to detect change in patient status, even if those changes are small?	*Pembrolizumab prolonged TTD in HRQoL compared with chemotherapy. Patients who were treated with pembrolizumab had stable or improved global health status/quality of life, whereas those who were treated with investigator's choice of chemotherapy experienced declines in global health status/quality of life. Combined with efficacy and safety outcomes, these data support pembrolizumab as standard of care for patients with platinum-refractory advanced urothelial cancer.*
**Can you make the magnitude of effect (if any) understandable to readers?**	

Can you provide an estimate of the difference in patients achieving a threshold of function or improvement, and the associated number needed to treat	**NA**

**Article 25**	**15-592; Atezolizumab and Bevacizumab; Metastatic Renal Cell carcinoma**
**First author & year of publication**	**McGregor, 2019**<br>**The article presents the results of a phase II study including PROs as an exploratory outcome.*
**Stated purpose of article**	*In this multicenter phase II trial, we evaluated atezolizumab combined with bevacizumab in patients with advanced renal cell carcinoma (RCC) with variant histology or any RCC histology with $ 20% sarcomatoid differentiation.*
**What were PROs measuring?**	
What concepts were the PROs used in the study measuring?	Quality of Life
What rationale (if any) for selection of concept or constructs did the authors provide?	Not reported
Were patients involved in the selection of outcomes measured by the PROs?	Not reported
**Omissions**	
Were there any important aspects of health (e.g., symptoms, function, perceptions) or quality of life (e.g. overall evaluation, satisfaction with life) that were omitted in this study from the perspectives of the patient, clinician, significant others...	Treatment-related adverse events – any grade [23]
**If randomized trials and other studies measured PROs, what were the instruments' measurement strategies?**	
Did investigators use instruments that yield a single indicator or index number, a profile, or a battery or instruments?	Battery: FKSI-19 and Brief Fatigue Inventory (BFI)
If investigators measure PROs, did they use specific or generic measures, or both?	Disease-specific and symptom-specific
Who exactly completed the instruments?	
**Did the instruments work in the way they were supposed to work - validity?**	
Had the instruments used been validated previously (provide reference)? Was evidence of prior validation for use in this population presented?	Rao D, Butt Z, Rosenbloom S, et al: A comparison of the Renal Cell Carcinoma-Symptom Index (RCC-SI) and the Functional Assessment of Cancer Therapy- Kidney Symptom Index (FKSI). J Pain Symptom Manage 38:291-298, 2009<br>Mendoza TR, Wang XS, Cleeland CS, et al: The rapid assessment of fatigue severity in cancer patients: Use of the Brief Fatigue Inventory. Cancer 85: 1186-1196, 1999
Were the instruments re-validated in this study?	No
**Did the instruments work in the way they were supposed to work – ability to measure**	
Are the PROs able to detect change in patient status, even if those changes are small?	*This safety profile was comparable to our study and reinforced by the quality-of-life data, which suggests a lack of decline in quality of life with treatment*
**Can you make the magnitude of effect (if any) understandable to readers?**	
Can you provide an estimate of the difference in patients achieving a threshold of function or improvement, and the associated number needed to treat	**NA**

**Article 26**	**UPCC 25514; Pembrolizumab; Oligometastatic NSCLC**
**First author & year of publication**	**Bauml, 2019**<br>**The article presents the results of a phase II study including PROs as a secondary outcome.*
**Stated purpose of article**	Herein we report the outcomes of our trial, including the effects of PD-L1 status and CD8 T-cell infiltration on outcomes.
**What were PROs measuring?**	
What concepts were the PROs used in the study measuring?	Quality of life
What rationale (if any) for selection of concept or constructs did the authors provide?	Not reported
Were patients involved in the selection of outcomes measured by the PROs?	Not reported

**Omissions**	
Were there any important aspects of health (e.g., symptoms, function, perceptions) or quality of life (e.g. overall evaluation, satisfaction with life) that were omitted in this study from the perspectives of the patient, clinician, significant others...	Treatment-related adverse events – any grade [24]
**If randomized trials and other studies measured PROs, what were the instruments' measurement strategies?**	
Did investigators use instruments that yield a single indicator or index number, a profile, or a battery or instruments?	Single instrument: FACT-L
If investigators measure PROs, did they use specific or generic measures, or both?	Cancer-specific and disease-specific
Who exactly completed the instruments?	
**Did the instruments work in the way they were supposed to work - validity?**	
Had the instruments used been validated previously (provide reference)? Was evidence of prior validation for use in this population presented?	Cella DF, Bonomi AE, Lloyd SR, Tulsky DS, Kaplan E, Bonomi P. Reliability and validity of the Functional Assessment of Cancer Therapy–Lung (FACT-L) quality of life instrument. Lung Cancer. 1995
Were the instruments re-validated in this study?	**No**
**Did the instruments work in the way they were supposed to work – ability to measure**	
Are the PROs able to detect change in patient status, even if those changes are small?	*Pembrolizumab after LAT yielded no new safety signals and no reduction in quality of life*
**Can you make the magnitude of effect (if any) understandable to readers?**	
Can you provide an estimate of the difference in patients achieving a threshold of function or improvement, and the associated number needed to treat	**NA**

**Article 27**	**CheckMate 214; nivolumab plus ipilimumab *vs* sunitinib; Renal cell carcinoma**
**First author & year of publication**	**Cella, 2019**
**Stated purpose of article**	Here, we describe further PRO results using the FKSI-19, Functional Assessment of Cancer Therapy-General (FACT-G), and EuroQol five dimensional three level (EQ-5D-3L) instruments
**What were PROs measuring?**	
What concepts were the PROs used in the study measuring?	Disease symptoms; HRQoL; general health status
What rationale (if any) for selection of concept or constructs did the authors provide?	We aimed to assess whether health-related quality of life (HRQoL) could be used to further describe the benefit-risk profile of nivolumab plus ipilimumab versus sunitinib.
Were patients involved in the selection of outcomes measured by the PROs?	Not reported
**Omissions**	
Were there any important aspects of health (e.g., symptoms, function, perceptions) or quality of life (e.g. overall evaluation, satisfaction with life) that were omitted in this study from the perspectives of the patient, clinician, significant others...	Treatment-related adverse events – any grade [25]
**If randomized trials and other studies measured PROs, what were the instruments' measurement strategies?**	
Did investigators use instruments that yield a single indicator or index number, a profile, or a battery or instruments?	Battery: FACT-G; FKSI-19; EQ-5D
If investigators measure PROs, did they use specific or generic measures, or both?	**Generic health status, cancer-specific, disease-specific**
Who exactly completed the instruments?	Patients
**Did the instruments work in the way they were supposed to work - validity?**	
Had the instruments used been validated previously (provide reference)? Was evidence of prior validation for use in this population presented?	*The FKSI-19 is a validated 19-item instrument that measures tumour- specific PROs in patients with kidney cancer.*<br>*The FACT-G is a validated 27-item instrument that measures general cancer HRQoL*<br>*The EQ-5D-3L is a validated, standardised instrument for measuring general health status*

	we used both a generic instrument designed for the general population (EQ-5D-3L) and cancer-specific instruments (FACT-G and FKSI-19) to measure HRQoL.<br>*Given its generic nature, EQ-5D-3L might not detect small changes in health that are important for HRQoL studies in patients with cancer. We believe that future trials in advanced renal cell carcinoma could preferentially use the FKSI-19 instrument, with the FACT-G and EQ-5D-3L also used depending on the interests of the investigating team.*<br>Rothrock NE, Jensen SE, Beaumont JL, et al. Development and initial validation of the NCCN/FACT symptom index for advanced kidney cancer. Value Health 2013<br>Cella DF, Tulsky DS, Gray G, et al. The Functional Assessment of Cancer Therapy scale: development and validation of the general measure. J Clin Oncol 1993<br>Euro-Qol Group. EuroQol—a new facility for the measurement of health-related quality of life. Health Policy 1990<br>Schrag A, Selai C, Jahanshahi M, Quinn NP. The EQ-5D—a generic quality of life measure—is a useful instrument to measure quality of life in patients with Parkinson's disease.<br>J Neurol Neurosurg Psychiatry 2000<br>Cella D, Yount S, Brucker PS, et al. Development and validation of a scale to measure disease-related symptoms of kidney cancer. Value Health 2007
Were the instruments re-validated in this study?	
**Did the instruments work in the way they were supposed to work – ability to measure**	
Are the PROs able to detect change in patient status, even if those changes are small?	*all PRO scores after baseline were higher in participants in the nivolumab plus ipilimumab group than in those in the sunitinib group. Overall, PRO scores were maintained or improved from baseline with nivolumab plus ipilimumab throughout the trial.*<br>*…analyses of changes from baseline over the first 103 weeks on study treatment showed improved HRQoL for nivolumab plus ipilimumab compared with sunitinib, with differences between treatment groups reaching significance at most timepoints for FKSI-19 and FACT-G total scores and most domain scores*
**Can you make the magnitude of effect (if any) understandable to readers?**	
Can you provide an estimate of the difference in patients achieving a threshold of function or improvement, and the associated number needed to treat	**NA**

**Article 28**	**CheckMate 057; nivolumab *vs* docetaxel; non-squamous non-small cell lung cancer**
**First author & year of publication**	**Reck, 2018**
**Stated purpose of article**	To evaluate health-related quality of life (HRQoL) with nivolumab or docetaxel using patient-reported outcomes.
**What were PROs measuring?**	
What concepts were the PROs used in the study measuring?	Disease-related symptoms and general health status
What rationale (if any) for selection of concept or constructs did the authors provide?	*advanced NSCLC is associated with a substantial symptom burden related to physical, social and psychological well-being. Poor health-related quality of life (HRQoL) at diagnosis and higher symptom burden at treatment initiation have been associated with shorter overall survival (OS) in patients with NSCLC. Therefore, prolonging OS while maintaining or improving HRQoL is an important treatment goal. Beyond traditional efficacy end- points (e.g. OS), HRQoL end-points based on patient- reported outcomes (PROs) data have become increasingly important for analysing novel therapies in clinical trials by providing the patient perspective on treatment benefit*
Were patients involved in the selection of outcomes measured by the PROs?	Not reported
**Omissions**	
Were there any important aspects of health (e.g., symptoms, function, perceptions) or quality of life (e.g. overall evaluation, satisfaction with life) that were omitted in this study from the perspectives of the patient, clinician, significant others...	Treatment-related adverse events – any grade [26]
**If randomized trials and other studies measured PROs, what were the instruments' measurement strategies?**	
Did investigators use instruments that yield a single indicator or index number, a profile, or a battery or instruments?	Battery: Lung Cancer Symptom Scale (LCSS) and EQ-5D

If investigators measure PROs, did they use specific or generic measures, or both?	Disease-specific and generic health status
Who exactly completed the instruments?	Patients
**Did the instruments work in the way they were supposed to work - validity?**	
Had the instruments used been validated previously (provide reference)? Was evidence of prior validation for use in this population presented?	*using two validated patient-reported instruments*<br>*Given that advanced NSCLC is associated with distinct and challenging symptoms, general HRQoL instruments such as the EQ-5D may be less sensitive than lung cancer-specific instruments for assessment of PROs in this population.*<br>Hollen P. Lung cancer symptom scale (LCSS). Charlottesville, VA: Quality of Life Research Associates, LLC; 2013<br>EuroQol Group. EuroQol d a new facility for the measurement of health-related quality of life. Health Policy 1990
Were the instruments re-validated in this study?	
**Did the instruments work in the way they were supposed to work – ability to measure**	
Are the PROs able to detect change in patient status, even if those changes are small?	Although the proportion of patients with disease-related symptom improvement, measured by the LCSS, by week 12 (a secondary study end-point) was similar in the two treatment arms, data at subsequent assessments consistently showed alleviation of symptom burden during treatment with nivolumab, whereas treatment with docetaxel was generally associated with stabilisation or worsening of symptoms<br>Regardless of assessment instrument used in this study, nivolumab demonstrated improved HRQoL and symptom burden compared with docetaxel, and these improvements occur relatively early in treatment.
**Can you make the magnitude of effect (if any) understandable to readers?**	
Can you provide an estimate of the difference in patients achieving a threshold of function or improvement, and the associated number needed to treat	**NA**

Table S6: PRO instruments completion rates

First author & year of publication	Name of study/trial (Trial ID)/Start of the trial	Total study sample size (n)	Control group (n)	Intervention group (n)	PRO tool completion at baseline: -> Control group (n)	PRO tool completion at baseline: -> Intervention group (n)	Last week reported	PRO tool completion at the end of the study: -> Control group (n)	PRO tool completion at the end of the study: -> Intervention group (n)	Reasons for attrition reported in the study
von Tresckow, 2019	KEYNOTE-087 (NCT02453594) 2015	210 206 PRO analysis	NA	Pembrolizumab 69 in cohort 1 79 in cohort 2 58 in cohort 3	NA	QLQ-C30: 189/206 66/69 cohort 1 72/79 cohort 2 51/58 cohort 3 EQ-5D : numbers not reported, but similar	Week 36	NA	QLQ-C30: 102/150 41/54 cohort 1 31/55 cohort 2 30/41 cohort 3 EQ-5D : numbers not reported, but similar	Discontinuation from the study due to adverse events, PD or death
Cella, 2016	CheckMate-025 (NCT01668784) 2012	821	411	410 Nivolumab	FKSI: 343/344* EQ-5D:344/344	FKSI-DRS: 361/362 EQ-5D:361/362	Week 104	FKSI-DRS: 9/*10* # EQ-5D: 9/*10*	FKSI-DRS: 20/*26* # EQ-5D: 20/*26*	At the end of treatment, in both groups, most patients who had discontinued treatment had done so because of disease progression
Schadendorf 2017	CheckMate-067 (NCT01844505) 2013	945	NA	316 Nivolumab 315 Ipilimumab 314 Nivo+Ipi	NA	QLQ-C30: N: 269/316 I: 259/315 N+I: 274/314 EQ-5D: N: 267/316 I: 258/315 N+I: 274/314	Week 79	NA	QLQ-C30: N: - I: 1/2 N+I: 2/2 EQ-5D: N: - I: 2/2 N+I: 1/2	Disease progression in the nivolumab and ipilimumab monotherapy groups and toxicity in Nivo+Ipi
Harrington, 2017	CheckMate-141 (NCT02105636) 2014	361	121	240 Nivolumab	QLQ-C30: 91/121 QLQ-H&N35: 91/121 EQ-5D: 90/121	QLQ-C30: 191/240 QLQ-H&N35: 193/240 EQ-5D: 191/240	Week 69	QLQ-C30: 0/0 QLQ-H&N35: 0/0 EQ-5D: 0/0	QLQ-C30: 2/2 QLQ-H&N35: 1/1 EQ-5D: 2/2	Specific information about reasons patients did not complete questionnaires were not collected, because this was not specified in the protocol.
Schadendorf 2016	KEYNOTE-002 (NCT01704287) 2012	540	167	177 Pembro. 10mg/kg 176 Pembro. 2mg/kg	QLQ-C30: 156/167	QLQ-C30: P. 10mg/kg: 170/177 P. 2mg/kg: 169/176	Week 36	QLQ-C30: 21/72	QLQ-C30: P. 10mg/kg: 39/75 P. 2mg/kg: 33/69	Disease progression or adverse events, death, and site administrative error
Petrella, 2017	KEYNOTE-006 (NCT1866319) 2013	834	NA	270 Pembro. Q2W 266 Pembro. Q3W 240 Ipilimumab	NA	QLQ-C30: P. Q2W: 267/270 P. Q3W: 263/266 Ipilimumab: 237/240	Week 36	NA	QLQ-C30: P. Q2W: 112/143 P. Q3W: 120/131 Ipilimumab: 54/128	Disease progression, adverse event, or death
Brahmer, 2017	KEYNOTE-024 (NCT02142738) 2014	305	148	151 Pembrolizumab	QLQ-C30: 137/148 QLQ-LC13: 136/148 EQ-5D: 137/148	QLQ-C30: 145/151 QLQ-LC13: 145/151 EQ-5D: 144/151	Week 24	QLQ-C30: 75/92 QLQ-LC13: 75/92 EQ-5D:75/92	QLQ-C30: 98/111 QLQ-LC13: 98/111 EQ-5D: 98/111	Death, adverse events, or disease progression
Long, 2016	CheckMate-066 (NCT01721772) 2013	418	208	210 Nivolumab	QLQ-C30: 135/208 EQ-5D: 135/208	QLQ-C30: 147/210 EQ-5D: 146/210	Week 73	QLQ-C30: 0/0 EQ-5D: 0/0	QLQ-C30: 1/1 EQ-5D: 1/1	The high attrition rate in the dacarbazine arm, possibly due to death and/or disease progression
Revicki, 2012	MDX010-20 (NCT00094653) 2004	676	136 (gp100)	137 Ipilimumab 403 Ipi+gp100	Numbers not reported Compliance of 95%		Week 12	QLQ-C30: gp100: 80	QLQ-C30: Ipilimumab: 85 Ipi+gp100: 236	Missing HRQL data at baseline were due to administrative errors. Reasons for missing Week 12 data were primarily due to disease progression, adverse events, or death

First author & year of publication	Name of study/trial (Trial ID)/Start of the trial	Total study sample size (n)	Control group (n)	Intervention group (n)	PRO tool completion at baseline: -> Control group (n)	PRO tool completion at baseline: -> Intervention group (n)	Last week reported	PRO tool completion at the end of the study: -> Control group (n)	PRO tool completion at the end of the study: -> Intervention group (n)	Reasons for attrition reported in the study
Coens, 2017	EORTC-18071 (NCT00636168) 2008	951	476	475 Ipilimumab	QLQ-C30: 444/476	QLQ-C30: 449/475	Week 108	QLQ-C30: 187/338	QLQ-C30: 167/359	Administrative failure (either by patient or staff), accounting for 962 (55%) of 1752 reasons reported for missing questionnaires.
Reck, 2017	CheckMate 017 (NCT01642004) 2012	272	135	137 Nivolumab	LCSS:106/137<br>EQ-5D:107/137	LCSS:106/135<br>EQ-5D:110/135	Week 98	LCSS:1/1<br>EQ-5D:1/1	LCSS:na<br>EQ-5D:na	Incomplete patient participation at each time point, reasons not reported
Reck, 2018	CheckMate 057 (NCT01673867) 2012	582	290	292 Nivolumab	LCSS:222/290<br>EQ-5D:232/290	LCSS:240/292<br>EQ-5D:244/292	Week 108	LCSS: 0<br>EQ-5D: 0	LCSS:1/2<br>EQ-5D:1/2	Incomplete patient participation at each time point, reasons not reported
Spigel, 2019	CheckMate 153 (NCT02066636) 2014	1426	NA	1426 Nivolumab	N/A	LCSS: 1095/1099<br>EQ-5D: 1095/1099	Week 42	N/A	LCSS: 246/299<br>EQ-5D: 246/299	Incomplete patient participation at each time point, reasons not reported
Cella, 2019	CheckMate 214 (NCT022317) 2014	1096	422 (intermediate and poor risk patients)	425 Nivolumab+Ipilimumab (intermediate and poor risk patients)	FKSI-19: 400/422<br>FACT-G: 400/422<br>EQ-5D: 403/422	FKSI-19: 413/425<br>FACT-G: 412/425<br>EQ-5D: 415/425	Week 133	FKSI-19: 1/1<br>FACT-G: 1/1<br>EQ-5D: 1/1	FKSI-19: 1/1<br>FACT-G: 1/1<br>EQ-5D: 1/1	Treatment discontinuation due to adverse events
Reck, 2019	CheckMate 227 (NCT02477826) 2015	1166	583	583 Nivolumab+Ipilimumab	LCSS:542/583<br>EQ-5D: 542/583	LCSS:540/583<br>EQ-5D:543/583	Week 102	LCSS:4/4<br>EQ-5D: 4/4	LCSS: 5/5<br>EQ-5D: 5/5	Exclusion of data on patients who discontinued therapy
Wendel Naumann, 2019	CheckMate 358 (NCT02488759) 2015	24 (19 cervical; 5 vaginal/vulvar)	NA	24 Nivolumab	NA	QLQ-C30 : 16/19<br>EQ-5D : 18/19<br>(only reported for cervical cohort)	Week 9	NA	QLQ-C30 : 13/19<br>EQ-5D : 13/19<br>(only reported for cervical cohort)	Not reported
Mansfield, 2020	IMpower133 (NCT027635) 2016	403	202	201 Atezolizumab+ Arboplatin/Etoposide	QLQ-C30: 179/202<br>QLQ-LC13: 176/202	QLQ-C30:175/201<br>QLQ-LC13:168/201	Week 54	34remained on study treatment and were eligible to complete PRO assessment		Relatively small number of patients eligible for PRO assessment at later time points, reasons not reported
Kaufmann, 2017 **§**	JAVELIN Merkel 20 (NCT02155647) 2014	88	NA	88 Avelumab	NA	FACT-M: 70/88<br>EQ-5D: 72/88	Week 25	NA	FACT-M: 31<br>EQ-5D: 32	QoL only assessed until disease progression, no other reasons for non-completion reported
Barlesi, 2018	KEYNOTE-010 (NCT01905657) 2013	1034	293	329 Pembro. 10mg/kg<br>331 Pembro. 2mg/kg<br>for tumor proportion score ≥ 1	QLQ-C30:273/293<br>QLQ-LC13: 273/293<br>EQ-5D: 237/255	QLQ-C30:<br>10mg/kg:311/329<br>2mg/kg:318/331<br>QLQ-LC13:<br>10mg/kg:319/331<br>2mg/kg:311/329<br>EQ-5D:<br>10mg/kg:293/310<br>2mg/kg:296/306	Week 36	QLQ-C30:21/293#<br>QLQ-LC13: 21/293#<br>EQ-5D: 13/255#	QLQ-C30:<br>10mg/kg:77/329#<br>2mg/kg:71/331#<br>QLQ-LC13:<br>10mg/kg:76/331#<br>2mg/kg:69/329<br>EQ-5D:<br>10mg/kg:65/310#<br>2mg/kg:58/306#	Disease progression, physician decision, adverse events or death

First author & year of publication	Name of study/trial (Trial ID)/Start of the trial	Total study sample size (n)	Control group (n)	Intervention group (n)	PRO tool completion at baseline: -> Control group (n)	PRO tool completion at baseline: -> Intervention group (n)	Last week reported	PRO tool completion at the end of the study: -> Control group (n)	PRO tool completion at the end of the study: -> Intervention group (n)	Reasons for attrition reported in the study
Vaughn, 2018 **§**	KEYNOTE 045 (NCT02256436) 2014	542 519 QoL analysis	253	266 Pembrolizumab	QLQ-C30 and / or EQ-5D: 242/253	QLQ-C30 and /or EQ-5D: 260/266	Week 51	QLQ-C30 and/or EQ-5D: 7	QLQ-C30 and/or EQ-5D : 56	Reasons why not included in primary analysis (at week 15) were: Disease progression, physician decision, adverse events, visit not scheduled or medication exclusion
Mazieres, 2019	KEYNOTE 407 (NCT02775435) 2016	559	280	278 Pembrolizumab + Chemotherapy (Carboplatin+Paclitaxel or nab-Paclitaxel)	QLQ-C30:264/278 QLQ-LC13: 263/268 EQ-5D: not reported	QLQ-C30:254/276 QLQ-LC13: 252/275 EQ-5D: not reported	Week 18	QLQ-C30:162/187 QLQ-LC13: 162/187 EQ-5D: not reported	QLQ-C30:191/217 QLQ-LC13: 191/217 EQ-5D: not reported	Adverse events, physician decision, disease progression, study withdrawal, or death, or patient had no scheduled visit
Bordoni, 2018	OAK (NCT02008227) 2014	850	425	425 Atezolizumab	QLQ-C30 and LC13:388/402	QLQ-C30 and LC13:413/421	Cycle 6 (week 18)	QLQ-C30 and LC13:151/156	QLQ-C30 and LC13:225/232	Patients did not complete form because of patient refusal or other reason
Hui 2019	PACIFIC (NCT02125461) 2014	713	237	476 Durvalumab	QLQ-C30:232/237 QLQ-LC13: 232/237 EQ-5D: not reported	QLQ-C30:474/476 QLQ-LC13: 472/476 EQ-5D: not reported	Week 48	QLQ-C30:88/102 QLQ-LC13: 88/102 EQ-5D: not reported	QLQ-C30:254/304 QLQ-LC13: 254/304 EQ-5D: not reported	Treatment discontinuation due to disease progression, adverse event, withdrawal, protocol non-adherence, meeting trial-specific criteria for discontinuation, other reasons
O'Donell, 2019 **§**	Study 1108 (NCT01693562) 2012	191	NA	191 Durvalumab (182 PRO assessment)	NA	FACT-BI :169/182 QLQ-C30:113/182	Day 337	NA	FACT-BI :10 QLQ-C30:7	Not reported Completion was lower than for FACT-BI because it was added to the protocol after study start and not administered at all scheduled visits
McGregor, 2019 **§**	15-592 (NCT02724878) 2016	60	NA	60 Atezolizumab+ bevacizumab	NA	FKSI-19:60/60 BFI:59/60	End of therapy	NA	FKSI-19:29 BFI:13	Discontinuation of therapy, toxicity, disease progression death; not reported for PRO completion
Bauml, 2019 **§**	UPCC 25514 (NCT02316002) 2105	51	NA	45 received pembrolizumab	NA	FACT-L: 38/45	Cycle 16	NA	FACT-L: 13	Not reported

*The total for PRO completion is for all randomised patients with non-missing data at baseline and data from at least one port-baseline visit.

The total number of patients have been calculated based on the percentage of completion provided in the study

§ These publications do not provide the total number remaining at the end of the study

Table S7: Symptom-related content of PRO instruments

EQ-5D
Pain / discomfort
Anxiety /

SF-36
Anxiety /
Pain
Energy
Tired

EORTC QLQ-
Fatigue
Dyspnea
Pain
Insomnia
Weakness
Lack of appetite
Nausea
Vomiting
Constipation
Diarrhea
Memory
Concentration
Irritability
Depression

FACT-G
Lack of energy
Nausea
Pain
Feel ill
Sad
Nervous
Sleeping well

FACT-L
Lack of energy
Nausea
Pain
Feel ill
Sad
Nervous
Sleeping well
Short of breath
Losing weight
Clear thinking
Cough
Hair loss
Good appetite
Tightness in
Difficulty

FACT-BL
Lack of energy
Nausea
Pain
Feel ill
Sad
Nervous
Sleeping well
Trouble
controlling my
Losing weight
Control of my
bowels
Urinate more
frequently
Diarrhea
Good appetite
Burns when I
urinate
Able to have and
maintain an
erection

FACT-M
Lack of energy
Nausea
Pain
Feel ill
Sad
Nervous
Sleeping well
Pain at my
melanoma site or
Changes in my
skin (lumps,
Short of breath
Headaches
Fevers
Swelling or
cramps in my
Good appetite
Aches and pains
in my bones
Blood in my stool
Memory /
concentration
Fatigue
SURGERY
Swelling at my
melanoma site
Numbness
Good range of
movement

FKSI-DRS
Lack of energy
Pain
Weight loss
Bone pain
Fatigue
Short of breath
Cough
Fevers
Blood in urine

FKSI-19
Lack of energy
Pain
Weight loss
Fatigue
Short of breath
Fevers
Bone pain
Cough
Weak all over
Blood in urine
Good appetite
Sleeping well
Nausea
Diarrhea

QLQ-LC13
Fatigue
Dyspnea
Pain
Insomnia
Weakness
Lack of appetite
Nausea
Vomiting
Constipation
Diarrhea
Memory
Concentration
Irritability
Depression
Cough
Dyspnea
Swallowing
Alopecia
Pain
Soreness
Tingling

QLQ-H&N35
Fatigue
Dyspnea
Pain
Insomnia
Weakness
Lack of appetite
Nausea
Vomiting
Constipation
Diarrhea
Memory
Concentration
Irritability
Depression
Pain mouth
Swallowing
Soreness mouth
Dry mouth
Sticky saliva
Smell
Taste
Cough
Hoarsening
Libido
Weight loss
Weight gain

LCSS
Loss of appetite
Fatigue
Cough
Shortness of
Pain
Hemoptysis

SDS
Nausea
Nausea (severity)
Appetite
Insomnia
Pain (frequency)
Pain (severity)
Fatigue
Bowel
Concentration
Breathing
Cough

FACIT-Fatigue

Brief Fatigue Inventory

Pain (single question)

Table S8: AEs reported in selected ICI-CT

CheckMate 057	nonsquamous NSCLC			
Total participants	287			
Niv_3 mg/kg	Any grade		Grade ≥3	
AE	n	%	n	%
Fatigue	46	16	3	1
Nausea	34	12	2	1
Dec. appetite	30	10	0	0
Asthenia	29	10	1	<1
Diarrhea	22	8	2	1
Peripheral edema	8	3	0	0
Myalgia	7	2	1	<1
Anemia	6	2	1	<1
Alopecia	1	<1	0	0
Neutropenia	1	<1	0	0
Febrile neutropenia	0	0	0	0
Leukopenia	0	0	0	0

observed in 10% or more of patients

Borghaei et al., NEJM, 2015

CheckMate 141	Head & Neck			
Total participants	236			
Niv_2 mg/kg	Any grade		Grade ≥3	
AE	n	%	n	%
Fatigue	33	14	5	2
Nausea	20	8	0	0
Rash	18	8	0	0
Dec. appetite	17	7	0	0
Pruritus	17	7	0	0
Diarrhea	16	7	0	0
Anemia	12	5	3	1
Asthenia	10	4	1	<1
Vomiting	8	3	0	0
Dry skin	7	3	0	0
Stomatitis	5	2	1	<1
Weight loss	4	2	0	0
Mucosal inflammation	3	1	0	0
neurologic/neuropathy	1	<1	0	0
Alopecia	0	0	0	0
Neutropenia	0	0	0	0

observed in 5% or more of patients

Ferris et al., NEJM, 2016

Impower 133	SCLC			
Total participants	198			
Atezo 1200 mg	Any grade *		Grade ≥3	
AE	n	%	n	%
Anemia	77	39	28	14
Neutropenia	72	36	46	23
Alopecia	69	35	0	0
Nausea	63	32	1	<1
Fatigue	42	21	3	2
Dec. appetite	41	21	2	1
Dec. neutrophil count	35	18	28	14
Thrombocitopenia	32	16	20	10
Vomiting	27	14	2	1
Leukopenia	25	13	10	5
Dec. platelet count	24	12	7	4
Constipation	20	10	1	<1
Diarrhea	19	10	4	2
Dec. white-cell count	16	8	6	3
Infusion-related reaction	10	5	4	2
Febrile neutropenia	6	3	6	3

observed in 10% or more of patients

Horn et al., NEJM, 2018

KEYNOTE-407	squamous NSCLC			
Total participants	278			
Pem 200 mg + Chemo	Any grade		Grade ≥3	
AE	n	%	n	%
Anemia	148	53	43	16
Alopecia	128	46	1	<1
Neutropenia	105	38	63	23
Nausea	99	36	3	1
Thrombocitopenia	85	31	19	7
Diarrhea	83	30	11	4
Dec. appetite	68	25	6	2
Constipation	64	23	2	<1
Fatigue	63	23	9	3
Asthenia	60	22	6	2
Arthralgia	57	21	4	1
Peripheral neuropathy	57	21	3	1
Vomiting	45	16	1	<1
Cough	37	13	2	<1
Dyspnea	36	13	4	1

observed in 15% or more of patients

Paz-Ares et al., NEJM, 2018

15-592	renal-cell carcinoma			
Total participants	60			
Atezo 1200 mg	Any grade		Grade ≥3	
AE	n	%	n	%
Fatigue	21	35	1	
Proteinuria	21	35	2	
Musculoskeletal pain	20	33	0	
Diarrhea	13	22	6	
Rash/skin condition	12	20	0	
Hypertension	11	18	2	
Pruritus	11	18	0	
Thyroid disorder	10	17	0	
Hepatitis	9	15	2	
Fever	8	13	1	
Mucositis	7	12	2	
Myalgia	7	12	0	

observed in 10% or more of patients

McGregor et al., JCO, 2019

CheckMate 025	renal-cell carcinoma			
Total participants	406			
Niv_3 mg/kg	Any grade		Grade ≥3	
AE	n	%	n	%
Fatigue	134	33	10	2
Nausea	57	14	1	<1
Pruritus	57	14	0	0
Diarrhea	50	12	5	1
Dec. appetite	48	12	2	<1
Rash	41	10	2	<1
Cough	36	9	0	0
Anemia	32	8	7	2
Dyspnea	30	7	3	<1
Peripheral edema	17	4	0	0
Pneumonitis	16	4	6	1
Dysgeusia	11	3	0	0
Mucosal inflammation	11	3	0	0
Hyperglycemia	9	2	5	1
Stomatitis	8	2	0	0
Hypertriglyceridemia	5	1	0	0
Epistaxis	3	<1	0	0

observed in 10% or more of patients

Motzer et al., NEJM, 2015

CheckMate 214	renal-cell carcinoma			
Total participants	547			
Niv_3 mg/kg+Ipi_1 mg/kg	Any grade		Grade ≥3	
AE	n	%	n	%
Fatigue	202	37	23	4
Pruritus	154	28	3	<1
Diarrhea	145	27	21	4
Rash	118	22	8	1
Nausea	109	20	8	1
Inc. lipase level	90	16	56	10
Hypothyroidism	85	16	2	<1
Dec. appetite	75	14	7	1
Asthenia	72	13	8	1
Vomiting	59	11	4	<1
Anemia	34	6	2	<1
Dysgeusia	31	6	0	0
Stomatitis	23	4	0	0
Dyspepsia	15	3	0	0
Mucosal inflammation	13	2	0	0
Hypertension	12	2	4	<1
Palmar-plantar erythrod	5	<1	0	0
Thrombocitopenia	2	<1	0	0

observed in 15% or more of patients

Motzer et al., NEJM, 2018

CheckMate 017	squamous-cell NSCLC			
Total participants	131			
Niv_3 mg/kg	Any grade		Grade ≥3	
AE	n	%	n	%
Fatigue	21	16	1	1
Dec. appetite	14	11	1	1
Asthenia	13	10	0	0
Nausea	12	9	0	0
Diarrhea	10	8	0	0
Arthralgia	7	5	0	0
Pyrexia	6	5	0	0
Pneumonitis	6	5	0	0
Rash	5	4	0	0
Mucosal inflammation	3	2	0	0
Myalgia	2	2	0	0
Anemia	2	2	0	0
peripheral neuropathy	1	1	0	0
Leukopenia	1	1	1	1
Neutropenia	1	1	0	0
Febrile neutropenia	0	0	0	0
Alopecia	0	0	0	0

observed in 5% or more of patients

Brahmer et al., NEJM, 2015

KEYNOTE-045	urothelial carcinoma			
Total participants	266			
Pem_200 mg	Any grade		Grade ≥3	
AE	n	%	n	%
Pruritus	52	20	0	0
Fatigue	37	14	3	1
Nausea	29	11	1	<1
Diarrhea	24	9	3	1
Dec. appetite	23	9	0	0
Hypothyroidism	17	6	0	0
Asthenia	15	6	1	<1
Pneumonitis	11	4	6	2
Anemia	9	3	2	<1
Colitis	6	2	3	1
Constipation	6	2	0	0
Nephritis	2	<1	2	<1
Severe skin reaction	2	<1	1	<1
Thyroiditis	2	<1	0	0
Preripheral sensory neur	2	<1	0	0
Dec. neutrophil count	1	<1	1	<1
Adrenal insufficiency	1	<1	1	<1
Peripheral neuropathy	1	<1	0	0

observed in 10% or more of patients

Bellmunt et al., NEJM, 2017

UPCC 25514	oligometastatic NSCLC			
Total participants	45			
Pem_200 mg	Any grade		Grade ≥3	
AE	n	%	n	%
Pain	19	42	1	2
Fatigue	16	36	0	0
Rash/skin condition	10	22	0	0
Dyspnea	8	18	1	2
Cough	7	16	0	0
Pruritus	7	16	0	0
Dizziness	6	13	0	0
Edema	6	13	0	0
Nausea	6	13	1	2
Pneumonitis	5	11	3	7
Dry eyes	5	11	0	0
Headache	5	11	0	0
Insomnia	5	11	0	0

observed in 10% or more of patients

Bauml et al., JAMA Oncology, 2019

* "Any grade" was calculated adding all grades

CheckMate 066 — melanoma

Total participants: 206

Niv_3 mg/kg	Any grade		Grade ≥3	
AE	n	%	n	%
Fatigue	41	20	0	0
Pruritus	35	17	1	<1
Nausea	34	17	0	0
Diarrhea	33	16	2	1
Rash	31	15	1	<1
Constipation	22	11	0	0
Vitiligo	22	11	0	0
Asthenia	21	10	0	0
Vomiting	13	6	1	<1
Neutropenia	0	0	0	0
Thrombocytopenia	0	0	0	0

observed in 10% or more of patients

Robert et al., NEJM, 2015

CheckMate 227 — advanced NSCLC

Total participants: 576

Niv_3 mg/kg+Ipi 1 mg/kg	Any grade		Grade ≥3	
AE	n	%	n	%
Diarrhea	98	17	10	2
Rash	98	17	9	2
Fatigue	83	14	10	2
Pruritus	82	14	3	<1
Dec. appetite	76	13	4	<1
Asthenia	59	10	8	1
Nausea	57	10	3	<1
Vomiting	28	5	2	<1
Constipation	26	5	0	0
Anemia	22	4	8	1
Neutropenia	1	<1	0	0

observed in 15% or more of patients

Hellmann et al., NEJM, 2019

CheckMate 153 — advanced NSCLC

Total participants: 1426

Niv_ 3 mg/kg	Any grade		Grade ≥3	
AE	n	%	n	%
Fatigue	296	21	34	2
Diarrhea	151	11	13	1
Nausea	128	9	3	<1
Hypothyroidism	115	8	1	<1
Dec. appetite	110	8	2	<1
Arthralgia	76	5	2	<1
Rash	69	5	4	<1
Pruritus	64	4	3	<1
Inc. TSH	29	2	1	<1

observed in 5% or more of patients

Spigel et al., J Thor Oncology, 2019

KEYNOTE-006 — melanoma

Ipi_3 mg/kg (Total participants 256)	Any grade		Grade ≥3		Pem_10 mg/kg Q2W (Total participants 278)	Any grade		Grade ≥3		Pem_10 mg/kg Q3W (Total participants 277)	Any grade		Grade ≥3	
AE	n	%	n	%	AE	n	%	n	%	AE	n	%	n	%
Pruritus	65	25	1	<1	Fatigue	58	21	0	0	Fatigue	53	19	1	<1
Diarrhea	58	23	8	3	Diarrhea	47	17	7	3	Diarrhea	40	14	3	1
Fatigue	39	15	3	1	Rash	41	15	0	0	Pruritus	39	14	0	0
Rash	37	14	2	<1	Pruritus	40	14	0	0	Rash	37	13	0	0
Nausea	22	9	1	<1	Asthenia	32	12	1	<1	Arthralgia	32	12	1	<1
Colitis	21	8	18	7	Hypothyroidism	28	10	1	<1	Nausea	31	11	1	<1
Asthenia	16	6	2	<1	Nausea	28	10	0	0	Asthenia	31	11	0	0
Arthralgia	13	5	2	<1	Arthralgia	26	9	0	0	Vitiligo	31	11	0	0
Hyperthyroidism	6	2	1	<1	Vitiligo	25	9	0	0	Hypothyroidism	24	9	0	0
Hypophysitis	6	2	4	2	Hyperthyroidism	18	6	0	0	Colitis	10	4	7	3
Hypothyroidism	5	2	0	0	Colitis	5	2	4	1	Hyperthyroidism	9	3	0	0
Vitiligo	4	2	0	0	Hepatitis	3	1	3	1	Hepatitis	5	2	5	2
Hepatitis	3	1	1	<1	Hypophysitis	1	<1	1	<1	Pneumonitis	5	2	1	<1
Myositis	1	<1	0	0	Pneumonitis	1	<1	0	0	Uveitis	3	1	0	0
Nephritis	1	<1	1	<1	Type 1 diabetes	1	<1	1	<1	Hypophysitis	2	<1	1	<1
Pneumonitis	1	<1	1	<1	Uveitis	1	<1	0	0	Myositis	2	<1	0	0
Type 1 diabetes	0	0	0	0	Myositis	0	0	0	0	Nephritis	1	<1	0	0
Uveitis	0	0	0	0	Nephritis	0	0	0	0	Type 1 diabetes	1	<1	1	<1

observed in 10% or more of patients

Robert et al., NEJM, 2015

OAK — NSCLC

Total participants: 609

Atezo 1200 mg	Any grade		Grade ≥3	
AE	n	%	n	%
Fatigue	163	27.0	17	3
Dec. appetite	143	24.0	2	<1
Cough	141	23.0	2	<1
Dyspnea	118	19.0	15	2
Asthenia	116	19.0	8	1
Nausea	108	18.0	4	<1
Pyrexia	108	18.0	1	<1
Constipation	107	18.0	2	<1
Diarrhea	94	15.0	4	<1
Vomiting	74	12.0	2	<1
Arthralgia	73	12.0	3	<1
Anemia	70	11.0	14	2
Back pain	67	11.0	7	1
Muskuloskeletal pain	64	10.0	4	<1
Peripheral edema	54	9.0	1	<1
Myalgia	39	6.0	1	<1
Peripheral neuropathy	24	4.0	0	0
Stomatitis	19	3.0	1	<1
Dysgeusia	18	3.0	0	0
Neutropenia	10	2.0	3	<1
Alopecia	3	<1	0	0
Febrile neutropenia	1	<1	1	<1

observed in 10% or more of patients

Rittmeyer et al., Lancet, 2017

EORTC-18071 — melanoma

Total participants: 471

Ipi_10 mg/kg	Any grade *		Grade ≥3	
AE	n	%	n	%
Diarrhea	231	49	46	10
Pruritus	203	43	11	2
Fatigue	189	40	10	2
Rash	185	39	6	1
Headache	152	32	4	<1
Weight loss	147	31	2	<1
Nausea	116	25	1	<1
ALT inc.	102	22	25	5
Liver function inc.	93	20	25	5
Hypophysitis	88	19	25	5
Pyrexia	82	17	5	1
AST inc.	78	17	20	4
Colitis	75	16	36	8
Weight inc.	69	15	1	<1
Cough	68	14	0	0
Abdominal pain	66	14	2	<1
Dec. appetite	65	14	1	<1
Vomiting	59	13	2	<1
Hypothyroidism	42	9	1	<1
Neurologic/neuropathy	21	4	9	2
Colitis ulcerative	1	<1	1	<1

observed in 10% or more of patients

Eggermont et al., Lancet Oncol, 2015

PACIFIC — NSCLC

Total participants: 475

Durva 10 mg/kg	Any grade		Grade ≥3	
AE	n	%	n	%
Cough	168	35.0	2	<1
Pneumonitis or radiation pneur	161	34	16	3.0
Fatigue	113	24	1	<1
Dyspnea	106	22	7	2
Diarrhea	87	18	3	<1
Pyrexia	70	15	1	<1
Dec. appetite	68	14	1	<1
Nausea	66	14	0	0
Pneumonia	62	13	21	4
Arthralgia	59	12	0	0
Pruritus	58	12	0	0
Rash	58	12	1	<1
Upper resp. tract infection	58	12	1	<1
Constipation	56	12	1	<1
Hypothyroidism	55	12	1	<1
Headache	52	11	1	<1
Asthenia	51	11	3	<1
Back pain	50	11	1	<1
Muskuloskeletal pain	39	8	3	<1
Anemia	36	8	14	3

observed in 10% or more of patients

Antonia et al., NEJM, 2017

CheckMate 067 — melanoma

Ipi_3 mg/kg (Total participants 311)	Any grade		Grade ≥3		Niv + Ipi (Total participants 313)	Any grade		Grade ≥3		Niv_3 mg/kg (Total participants 313)	Any grade		Grade ≥3	
AE	n	%	n	%	AE	n	%	n	%	AE	n	%	n	%
Pruritus	110	35	1	<1	Diarrhea	138	44	29	9.3	Fatigue	107	34	4	1
Diarrhea	103	33	19	6	Rash	126	40	15	4.8	Rash	81	26	2	<1
Rash	102	33	6	2	Fatigue	110	35	13	4.2	Diarrhea	60	19	7	2
Fatigue	87	28	3	1	Pruritus	104	33	6	1.9	Pruritus	59	19	0	0
Nausea	50	16	2	<1	Nausea	81	26	7	2.2	Nausea	41	13	0	0
Dec. appetite	39	13	1	<1	Pyrexia	58	19	2	<1	Dec. appetite	34	11	0	0
Colitis	36	12	27	9	Dec. appetite	56	18	4	1.3	Hypothyroidism	27	9	0	0
Headache	24	8	1	<1	ALT inc.	55	18	26	8.3	Arthralgia	24	8	0	0
Vomiting	23	7	1	<1	AST inc.	48	15	19	6.1	Headache	23	7	0	0
Pyrexia	21	7	1	<1	vomiting	48	15	8	2.6	Vomiting	20	6	1	<1
Arthralgia	19	6	0	0	Hypothyroidism	47	15	1	<1	Pyrexia	18	6	0	0
Dyspnea	13	4	0	0	Colitis	37	12	24	7.7	Dyspnea	14	4	1	<1
Hypothyroidism	13	4	0	0	Arthralgia	33	11	1	<1	ALT inc.	12	4	4	1
ALT inc.	12	4	5	2	Dyspnea	32	10	2	<1	AST inc.	12	4	3	1
AST inc.	11	4	2	<1	Headache	32	10	1	<1	Colitis	4	1	2	<1

observed in 10% or more of patients

Larkin et al., NEJM, 2015

* "Any grade" was calculated adding all grades

MDX010-20	melanoma			
Total participants	131			
Ipi_3 mg/kg	Any grade		Grade ≥3*	
AE	n	%	n	%
Fatigue	55	42	9	7
Nausea	46	35	3	2
Diarrhea	43	33	7	5
Dec. appetite	35	27	2	2
Pruritus	32	24	0	0
Vomiting	31	24	3	2
Constipation	27	21	3	2
Rash	25	19	1	<1
Cough	21	16	0	0
Abdominal pain	20	15	2	2
Dyspnea	19	15	5	4
Headache	19	15	3	2
Pyrexia	16	12	0	0
Anemia	15	11	4	3
Colitis	10	8	7	5
Hypopituitarism	3	2	2	2
Vitiligo	3	2	0	0
Adrenal insuf.	2	2	0	0
ALT inc.	2	2	0	0
Hypophysitis	2	2	2	2
Hypothyroidism	2	2	0	0
AST inc.	1	<1	0	0
Hepatitis	1	<1	0	0

observed in 15% or more of patients

Hodi et al., NEJM, 2010

KEYNOTE-087	Hodgkin lymphoma			
Total participants	210			
Pem_200 mg	Any grade *		Grade ≥3	
AE	n	%	n	%
Hypothyroidism	26	12	1	<1
Pyrexia	22	10	1	<1
Fatigue	19	9	1	<1
Rash	16	8	0	0
Diarrhea	15	7	2	1
Headache	13	6	0	0
Cough	12	6	1	<1
Nausea	12	6	0	0
Neutropenia	11	5	5	2
Arthralgia	9	4	1	<1
Muscle spams	8	4	0	0
Pruritus	8	4	0	0
Vomiting	8	4	0	0
Dyspnea	7	3	2	1
Upper resp. infection	7	3	0	0
Constipation	6	3	0	0
Chills	5	2	0	0
Myalgia	5	2	0	0
Back pain	4	2	0	0
Asthenia	3	1	0	0
Nasal congestion	3	1	0	0
Bronchitis	2	1	0	0
Insomnia	2	1	0	0
Nasopharyngitis	2	1	0	0
Anemia	1	0	0	0
Oropharngeal pain	1	0	0	0
Sinusitis	1	0	0	0

observed in 5% or more of patients

Chen et al., JCO, 2017

Javelin Merkel 200	Merkel cell carcinoma			
Total participants	39			
Avelumab 10 mg/kg	Any grade		Grade ≥3	
AE	n	%	n	%
Fatigue	9	23	0	0
Infusion-related reaction	9	23	1	3
Asthenia	3	8	0	0
Inc. Lipase	3	8	1	3
Inc. ALT	3	8	1	3
Arthralgia	2	5	0	0
Inc. CPK	2	5	1	3
Chills	2	5	0	0
Diarrhea	2	5	0	0
Dry mouth	2	5	0	0
Dyspnea	2	5	0	0
Eosinophilia	2	5	0	0
Nausea	2	5	0	0
Pruritus	2	5	0	0
Pyrexia	2	5	0	0
Rash maculo-papular	2	5	0	0
Dec. Weight	2	5	0	0
Autoimmune nephritis	1	3	1	3
Cholangitis	1	3	1	3
Inc. AST	1	3	1	3
Gait disturbance	1	3	1	3
Paraneoplastic encephalomyelit	1	3	1	3
Polyneuropathy	1	3	1	3
Paraneoplastic syndrome	1	3	1	3
Inc. Troponin	1	3	1	3

observed in 5% or more of patients

D'Angelo et al., JAMA Oncology, 2018

KEYNOTE-024	NSCLC			
Total participants	154			
Pem_200 mg	Any grade		Grade ≥3	
AE	n	%	n	%
Diarrhea	22	14	6	4
Fatigue	16	10	2	1
Pyrexia	16	10	0	0
Nausea	15	10	0	0
Dec. appetite	14	9	0	0
Hypothyroidism	14	9	0	0
Hyperthyroidism	12	8	0	0
Pneumonitis	9	6	4	3
Anemia	8	5	3	2
Infusion reaction	7	5	0	0
Constipation	6	4	0	0
Severe skin reactions	6	4	6	4
Stomatitis	4	3	0	0
Thyroiditis	4	3	0	0
Vomiting	4	3	1	<1
Colitis	3	2	2	1
Inc. blood creatinine	3	2	0	0
Myositis	3	2	0	0
Dysgeusia	1	<1	0	0
Hypophysitis	1	<1	1	<1
Leukopenia	1	<1	0	0
Nephritis	1	<1	1	<1
Neutropenia	1	<1	0	0
Pancreatitis	1	<1	1	<1
Type 1 diabetes	1	<1	1	<1
Platelet count dec.	0	0	0	0
Thrombocytopenia	0	0	0	0

observed in 10% or more of patients

Reck et al., NEJM, 2016

Study 1108	urothelial carcinoma			
Total participants	191			
Durva 10 mg/kg	Any grade		Grade ≥3	
AE	n	%	n	%
Fatigue	37	19	0	0
Dec. appetite	18	9	0	0
Diarrhea	16	8	1	<1
Rash	14	7	0	0
Nausea	13	7	0	0
Arthralgia	11	6	0	0
Pyrexia	11	6	0	0
Pruritus	10	5	0	0
Hypothyroidism	9	5	0	0
Inc. ALT	8	4	2	1
Inc. AST	6	3	3	2
Vomiting	6	3	0	0
Inc. GGT	6	3	2	1
Dyspnea	4	2	0	0
Inc. blood ALP	4	2	1	<1
Thrombocytopenia	4	2	0	0
Hypertension	3	2	2	1
Anemia	2	1	1	<1
Ras maculo-papular	2	1	1	<1
Infusion-related reaction	2	1	1	<1
Inc transaminases	2	1	1	<1
Autoimmune hepatitis	2	1	1	<1
Tumor flare	2	1	1	<1
Acute kidnez injury	1	<1	1	<1
Atrial fibrilation	1	<1	1	<1
Hyponatremia	1	<1	0	0
Colitis	1	<1	0	0
Dec.weight	1	<1	0	0
Leukopenia	1	<1	0	0

observed in 5% or more of patients

Powles et al., JAMA Oncology, 2017

KEYNOTE-010					advanced NSCLC				
Total participants	339				343				
Pem_2 mg/kg	Any grade		Grade ≥3		Pem_10 mg/kg	Any grade		Grade ≥3	
AE	n	%	n	%	AE	n	%	n	%
Dec. appetite	46	14	3	1	Dec. appetite	33	10	1	<1
Fatigue	46	14	4	1	Fatigue	49	14	6	2
Nausea	37	11	1	<1	Nausea	31	9	2	1
Rash	29	9	1	<1	Rash	44	13	1	<1
Hypothyroidism	28	8	0	0	Hypothyroidism	28	8	0	0
Diarrhea	24	7	2	1	Diarrhea	22	6	0	0
Asthenia	20	6	1	<1	Hyperthyroidism	20	6	1	<1
Pneumonitis	16	5	7	2	Asthenia	19	6	2	1
Stomatitis	13	4	0	0	Pneumonitis	15	4	7	2
Hyperthyroidism	12	4	0	0	Anemia	14	4	1	<1
Anemia	10	3	3	1	Severe skin reaction	7	2	6	2
Colitis	4	1	3	1	Stomatitis	7	2	1	<1
Severe skin reaction	4	1	3	1	Adrenal insufficiency	3	1	1	<1
Pancreatitis	3	1	2	1	Colitis	2	1	1	<1
Alopecia	3	1	0	0	Tipe 1 diabetes	2	1	1	<1
Adrenal insufficiency	2	1	0	0	Alopecia	2	1	0	0
Myositis	2	1	0	0	Autoimmune hepatitis	2	1	0	0
Thyroiditis	2	1	0	0	Hypophysitis	1	<1	1	<1
Autoimmune hepatitis	1	<1	1	<1	Myositis	1	<1	0	0
Hypophysitis	1	<1	1	<1	Neutropenia	1	<1	0	0
Tipe 1 diabetes	1	<1	1	<1	Pancreatitis	0	0	0	0
Neutropenia	1	<1	0	0	Thyroiditis	0	0	0	0

observed in 10% or more of patients

Herbst et al., Lancet, 2016

KEYNOTE-002					melanoma				
Total participants	178				##				
Pem_2 mg/kg	Any grade *		Grade ≥3		Pem_10 mg/kg	Any grade *		Grade ≥3	
AE	n	%	n	%	AE	n	%	n	%
Fatigue	40	22	2	1	Fatigue	52	29	1	<1
Pruritus	37	21	0	0	Pruritus	42	23	0	0
Rash	21	12	0	0	Rash	18	10	0	0
Diarrhea	15	8	0	0	Diarrhea	19	11	2	1
Arthralgia	13	7	1	<1	Dec. appetite	17	9	2	1
Vitiligo	10	6	0	0	Nausea	16	9	1	<1
Dry skin	9	5	0	0	Hypothyroidism	13	7	0	0
Hypothyroidism	9	5	0	0	Arthralgia	11	6	1	<1
Myalgia	9	5	2	1	Maculopapular rash	10	6	1	<1
Nausea	8	4	0	0	Vomiting	10	6	1	<1
Dec. appetite	8	4	0	0	Constipation	9	5	0	0
Asthenia	6	3	1	<1	Dry skin	9	5	0	0
Alopecia	5	3	0	0	Vitiligo	9	5	0	0
Anemia	5	3	1	<1	Asthenia	8	4	1	<1
Constipation	5	3	0	0	Anemia	7	4	0	0
Maculopapular rash	5	3	1	<1	Myalgia	7	4	0	0
Neurologic/neuropathy	2	1	0	0	Paraesthesia	2	1	0	0
Thrombocytopenia	2	1	0	0	Alopecia	1	<1	0	0
Vomiting	2	1	1	<1	Neutropenia	1	<1	0	0
Neutropenia	1	<1	0	0	Platelet count dec.	1	<1	0	0
Paraesthesia	1	<1	0	0	Thrombocytopenia	1	<1	1	<1
Leukopenia	0	0	0	0	Leukopenia	0	0	0	0
Platelet count dec.	0	0	0	0	Neurologic/neuropathy	0	0	0	0

observed in 5% or more of patients

Ribas et al., Lancet Oncol, 2015

CheckMate 358	Cervical/Vaginal/Vulvar cancer							
Cervical cancer= 19					Vaginal/Vulvar cancers= 5			
Niv 240 mg	ny grade		Grade ≥3		Any grade		Grade ≥3	
AE	n	%	n	%	n	%	n	%
Diarrhea	4	21	1	5	0	0	0	0
Fatigue	3	16	0	0	1	20	0	0
Pneumonitis	2	11	1	5	0	0	0	0
Abdominal pain	2	11	0	0	1	20	0	0
Stomatitis	2	11	0	0	0	0	0	0
Dry eye	2	11	0	0	0	0	0	0
Arthralgia	2	11	0	0	1	20	0	0
Dec. appetite	1	5	0	0	2	40	0	0

observed in 10% or more of patients

Naumann et al., JCO, 2019

Table S9: AEs differentiated into multi-symptom

	AE	Multi-symptom AEs							
Considered partially covered as soon as 2 or more of the symptoms used to define the AE were present in the PRO instrument	**Hyperthyroidism**	weight loss	tachycardia palpitations arrhythmia	increased appetite	fatigue	tremor	sweating	irritability anxiety	changes in menstrual patterns
	**Hypothyroidism**	weight gain	bradycardia	constipation	fatigue	muscle weakness	dry skin	hoarseness	
	**Hypophysitis**	weight changes	headache	decreased appetite	fatigue	muscle weakness	visual trouble	related to hypogonadism (sexual hormones)	
	**Colitis**	diarrhea	abdominal pain	decreased appetite	fatigue	cramps	blood in stool	chills	fever
	**Hepatitis**	nausea/vomiting	abdominal pain	decreased appetite	fatigue	dark urine	itchyness	jaundice	fever
	**Anemia**	weakness	palpitations	headache	fatigue	shortness of breath	difficulty concentrating	dizziness	
	**Pneumonitis**	weight loss	cough	decreased appetite	fatigue	shortness of breath / dyspnea			
	**Stomatitis**	soreness mouth	pain						
	**Hypertension**	headache	palpitations	shortness of breath	fatigue				

		Items related to pain							
Considered partially covered as they are specific to a system or localization	**Headache**								
	**Myalgia**	muscle pain							
	**Arthralgia**	joint pain	stiffness	swelling	redness				
	**Abdominal pain**								

REFERENCES

1. Chen R., Zinzani P.L., Fanale M.A., Armand P., Johnson N.A., Brice P., *et al.* (2017) Phase II Study of the Efficacy and Safety of Pembrolizumab for Relapsed/Refractory Classic Hodgkin Lymphoma. *J Clin Oncol* 35(19):2125-2132.
2. Motzer R.J., Escudier B., McDermott D.F., George S., Hammers H.J., Srinivas S., *et al.* (2015) Nivolumab versus Everolimus in Advanced Renal-Cell Carcinoma. *N Engl J Med* 373(19):1803-1813.
3. Larkin J., Chiarion-Sileni V., Gonzalez R., Grob J.J., Cowey C.L., Lao C.D., *et al.* (2015) Combined Nivolumab and Ipilimumab or Monotherapy in Untreated Melanoma. *N Engl J Med* 373(1):23-34.
4. Ferris R.L., Blumenschein G., Jr., Fayette J., Guigay J., Colevas A.D., Licitra L., *et al.* (2016) Nivolumab for Recurrent Squamous-Cell Carcinoma of the Head and Neck. *N Engl J Med* 375(19):1856-1867.
5. Eggermont A.M., Chiarion-Sileni V., Grob J.J., Dummer R., Wolchok J.D., Schmidt H., *et al.* (2015) Adjuvant ipilimumab versus placebo after complete resection of high-risk stage III melanoma (EORTC 18071): a randomised, double-blind, phase 3 trial. *Lancet Oncol* 16(5):522-530.
6. Ribas A., Puzanov I., Dummer R., Schadendorf D., Hamid O., Robert C., *et al.* (2015) Pembrolizumab versus investigator-choice chemotherapy for ipilimumab-refractory melanoma (KEYNOTE-002): a randomised, controlled, phase 2 trial. *Lancet Oncol* 16(8):908-918.
7. Hodi F.S., O'Day S.J., McDermott D.F., Weber R.W., Sosman J.A., Haanen J.B., *et al.* (2010) Improved survival with ipilimumab in patients with metastatic melanoma. *N Engl J Med* 363(8):711-723.
8. Robert C., Schachter J., Long G.V., Arance A., Grob J.J., Mortier L., *et al.* (2015) Pembrolizumab versus Ipilimumab in Advanced Melanoma. *N Engl J Med* 372(26):2521-2532.
9. Reck M., Rodriguez-Abreu D., Robinson A.G., Hui R., Csoszi T., Fulop A., *et al.* (2016) Pembrolizumab versus Chemotherapy for PD-L1-Positive Non-Small-Cell Lung Cancer. *N Engl J Med* 375(19):1823-1833.
10. Robert C., Long G.V., Brady B., Dutriaux C., Maio M., Mortier L., *et al.* (2015) Nivolumab in previously untreated melanoma without BRAF mutation. *N Engl J Med* 372(4):320-330.
11. Herbst R.S., Baas P., Kim D.W., Felip E., Perez-Gracia J.L., Han J.Y., *et al.* (2016) Pembrolizumab versus docetaxel for previously treated, PD-L1-positive, advanced non-small-cell lung cancer (KEYNOTE-010): a randomised controlled trial. *Lancet* 387(10027):1540-1550.
12. Rittmeyer A., Barlesi F., Waterkamp D., Park K., Ciardiello F., von Pawel J., *et al.* (2017) Atezolizumab versus docetaxel in patients with previously treated non-small-cell lung cancer (OAK): a phase 3, open-label, multicentre randomised controlled trial. *Lancet* 389(10066):255-265.
13. Antonia S.J., Villegas A., Daniel D., Vicente D., Murakami S., Hui R., *et al.* (2017) Durvalumab after Chemoradiotherapy in Stage III Non-Small-Cell Lung Cancer. *N Engl J Med* 377(20):1919-1929.
14. D'Angelo S.P., Russell J., Lebbe C., Chmielowski B., Gambichler T., Grob J.J., *et al.* (2018) Efficacy and Safety of First-line Avelumab Treatment in Patients With Stage IV

Metastatic Merkel Cell Carcinoma: A Preplanned Interim Analysis of a Clinical Trial. *JAMA Oncol* 4(9):e180077.

15. Horn L., Mansfield A.S., Szczesna A., Havel L., Krzakowski M., Hochmair M.J.*, et al.* (2018) First-Line Atezolizumab plus Chemotherapy in Extensive-Stage Small-Cell Lung Cancer. *N Engl J Med* 379(23):2220-2229.
16. Paz-Ares L., Luft A., Vicente D., Tafreshi A., Gumus M., Mazieres J.*, et al.* (2018) Pembrolizumab plus Chemotherapy for Squamous Non-Small-Cell Lung Cancer. *N Engl J Med* 379(21):2040-2051.
17. Naumann R.W., Hollebecque A., Meyer T., Devlin M.J., Oaknin A., Kerger J.*, et al.* (2019) Safety and Efficacy of Nivolumab Monotherapy in Recurrent or Metastatic Cervical, Vaginal, or Vulvar Carcinoma: Results From the Phase I/II CheckMate 358 Trial. *J Clin Oncol* 37(31):2825-2834.
18. Powles T., O'Donnell P.H., Massard C., Arkenau H.T., Friedlander T.W., Hoimes C.J.*, et al.* (2017) Efficacy and Safety of Durvalumab in Locally Advanced or Metastatic Urothelial Carcinoma: Updated Results From a Phase 1/2 Open-label Study. *JAMA Oncol* 3(9):e172411.
19. Brahmer J., Reckamp K.L., Baas P., Crino L., Eberhardt W.E., Poddubskaya E.*, et al.* (2015) Nivolumab versus Docetaxel in Advanced Squamous-Cell Non-Small-Cell Lung Cancer. *N Engl J Med* 373(2):123-135.
20. Hellmann M.D., Paz-Ares L., Bernabe Caro R., Zurawski B., Kim S.W., Carcereny Costa E.*, et al.* (2019) Nivolumab plus Ipilimumab in Advanced Non-Small-Cell Lung Cancer. *N Engl J Med* 381(21):2020-2031.
21. Spigel D.R., McCleod M., Jotte R.M., Einhorn L., Horn L., Waterhouse D.M.*, et al.* (2019) Safety, Efficacy, and Patient-Reported Health-Related Quality of Life and Symptom Burden with Nivolumab in Patients with Advanced Non-Small Cell Lung Cancer, Including Patients Aged 70 Years or Older or with Poor Performance Status (CheckMate 153). *J Thorac Oncol* 14(9):1628-1639.
22. Bellmunt J., de Wit R., Vaughn D.J., Fradet Y., Lee J.L., Fong L.*, et al.* (2017) Pembrolizumab as Second-Line Therapy for Advanced Urothelial Carcinoma. *N Engl J Med* 376(11):1015-1026.
23. McGregor B.A., McKay R.R., Braun D.A., Werner L., Gray K., Flaifel A.*, et al.* (2020) Results of a Multicenter Phase II Study of Atezolizumab and Bevacizumab for Patients With Metastatic Renal Cell Carcinoma With Variant Histology and/or Sarcomatoid Features. *J Clin Oncol* 38(1):63-70.
24. Bauml J.M., Mick R., Ciunci C., Aggarwal C., Davis C., Evans T.*, et al.* (2019) Pembrolizumab After Completion of Locally Ablative Therapy for Oligometastatic Non-Small Cell Lung Cancer: A Phase 2 Trial. *JAMA Oncol*.
25. Motzer R.J., Tannir N.M., McDermott D.F., Aren Frontera O., Melichar B., Choueiri T.K.*, et al.* (2018) Nivolumab plus Ipilimumab versus Sunitinib in Advanced Renal-Cell Carcinoma. *N Engl J Med* 378(14):1277-1290.
26. Borghaei H., Paz-Ares L., Horn L., Spigel D.R., Steins M., Ready N.E.*, et al.* (2015) Nivolumab versus Docetaxel in Advanced Nonsquamous Non-Small-Cell Lung Cancer. *N Engl J Med* 373(17):1627-1639.
