# Supplementary figures and images for: Patient-reported outcome instruments used in immune-checkpoint inhibitor clinical trials in oncology: a systematic review
Source: J Patient Rep Outcomes. 2020 Jul 16;4:58. doi: 10.1186/s41687-020-00210-z (PMC7364679; doi:10.1186/s41687-020-00210-z)

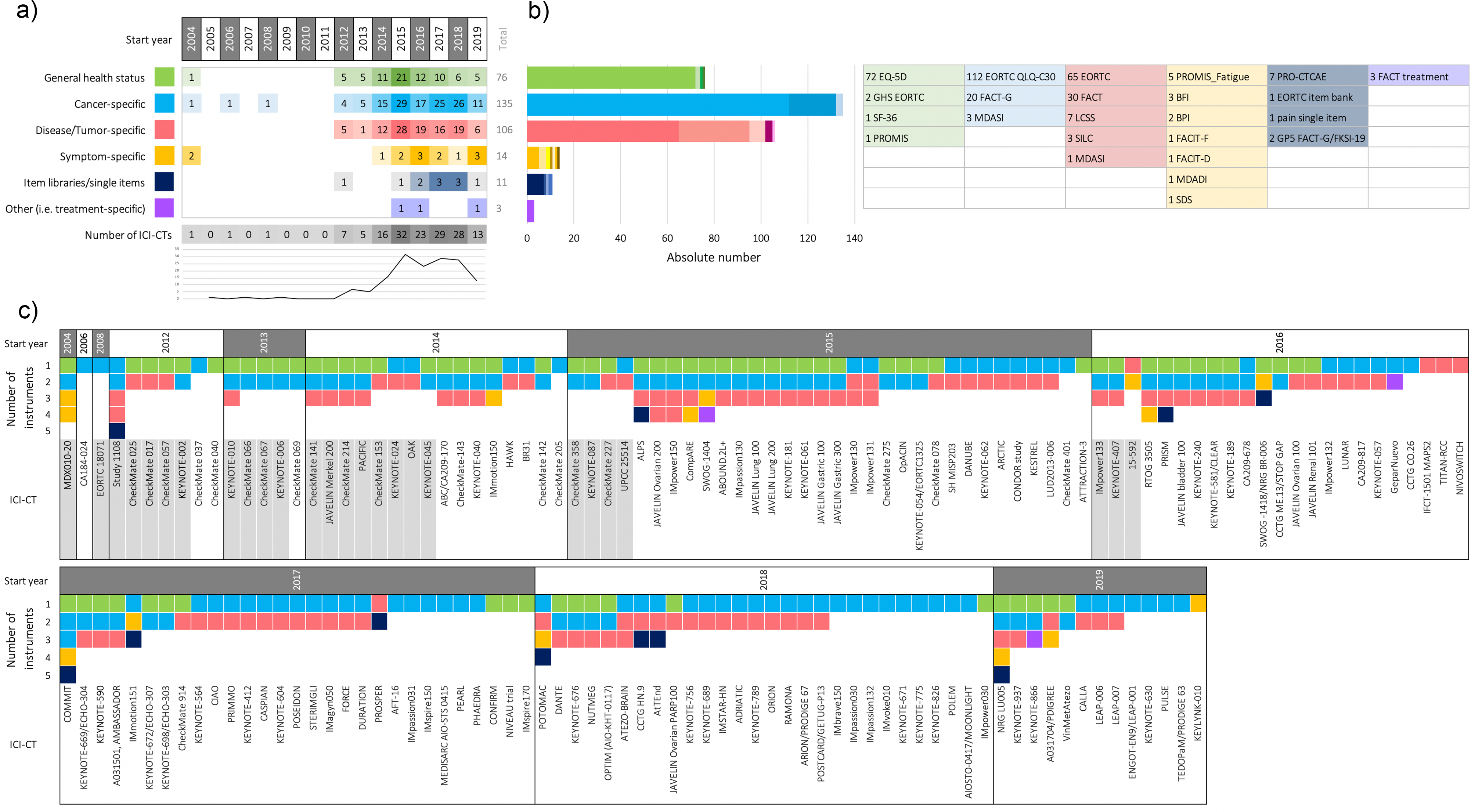

Supplement: Supplementary file 2 — Additional file 2: Figure S1. Frequency of use and combination of PRO instruments in ICI-CTs (n = 156). a Number of PRO instruments divided into categories and start year of the ICI-CTs. Green: general health status; Blue: cancer-specific; Red: disease/tumor-specific; Yellow: symptom-specific; Dark blue: items libraries/single items; Purple: other (e.g. treatment-specific). b Absolute number of instruments across all ICI-CTs divided per category. The table shows the different instruments per category. c Number and combination of PRO instruments per ICI-CT per year of start of the trial. For FACT questionnaires that include core domains of the FACT-G, both cancer-specific and disease specific have been depicted (see Table S4 for more detail). For ICI-CTs colored in light grey PRO results have been published (n = 26) [file 41687_2020_210_MOESM2_ESM.tif]

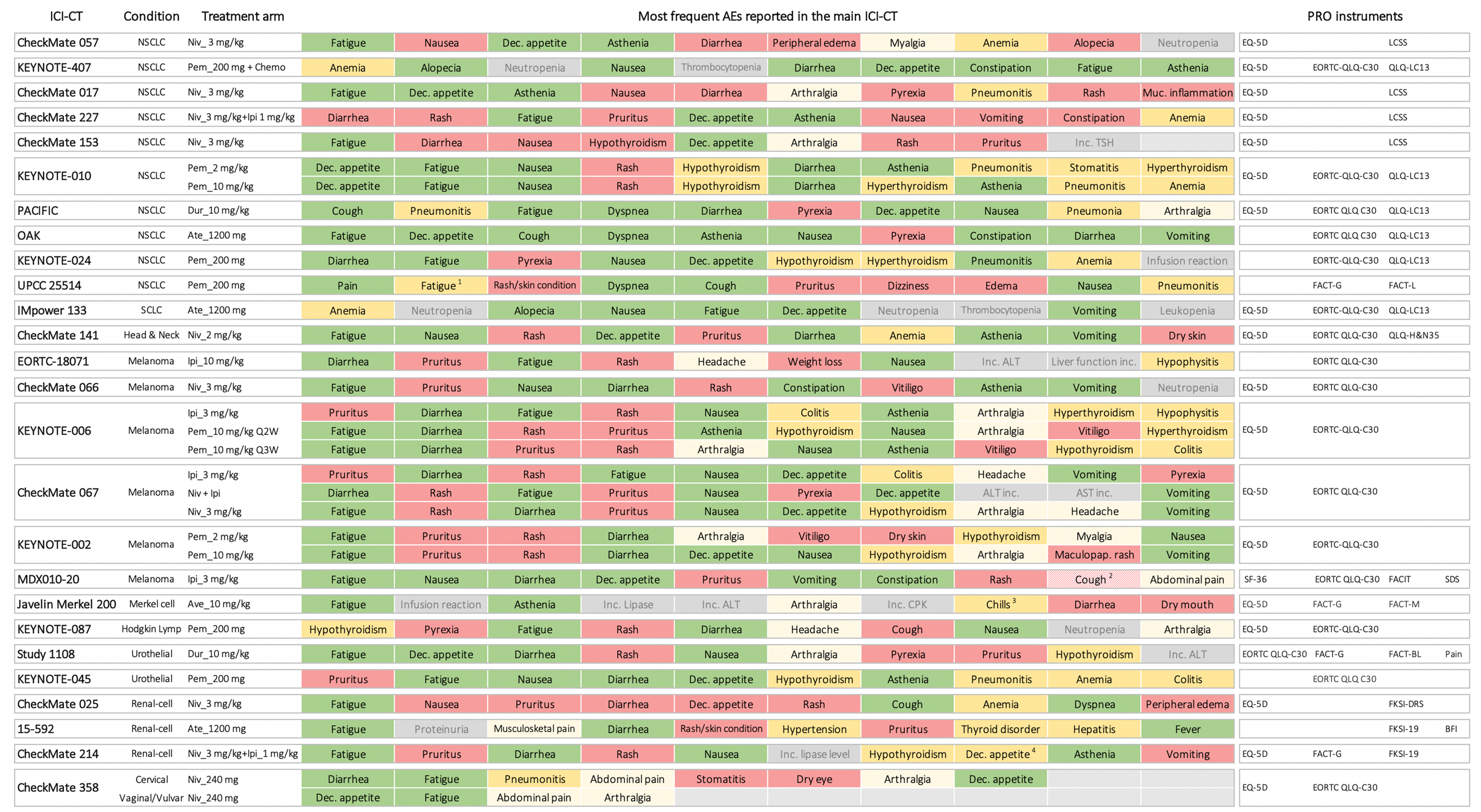

Supplement: Supplementary file 3 — Additional file 3: Figure S2. Comparison of PRO instruments’ symptom-related content and AEs reported in ICI-CTs with published PRO results (n = 26). The most frequently reported AEs (any grade) are shown for the 26 ICI-CTs identified. AE frequency (most common to least common) is depicted from left to right respectively. Symptom-related content from each PRO instrument was compared to the AEs in the corresponding trial arm. The PRO instruments used in each ICI-CT is shown. For a detailed PRO instrument content, see Table S8. Green: AEs covered; Yellow: AEs partially covered; Light yellow: AEs partially covered related to specific types of pain; Red: AEs not covered. Laboratory tests results (grey) were not included in calculations. “Asthenia” was considered covered by “fatigue” following the National Cancer Institute toxicity grading scale version 3 that included asthenia, lethargy, and malaise under the umbrella of “fatigue”. (1) “Fatigue” was considered partially covered because the term included in the FACT-G was “lack of energy”; (2) While the study protocol announced the use of SF-36, EORTC QLQ-C30, FACIT-Fatigue, and SDS questionnaires, only results from the EORTC QLQ-C30 were reported in the full-text publication. “Cough” would be covered by the SDS instrument; (3) “Chills” was partially covered as related to “fever”, included in the FACT-G; (4) “Dec. appetite” was considered as partially covered by the term “good appetite” in the FKSI-19. Ipi, Ipilimumab; Niv, Nivolumab; Pem, Pembrolizumab; Ate, Atezolizumab; Dur, Durvalumab; Inc., increased; Dec, decreased [file 41687_2020_210_MOESM3_ESM.tif]
